# Reductive Cross-Coupling of Olefins via a Radical Pathway

**DOI:** 10.1021/jacs.3c11285

**Published:** 2023-11-10

**Authors:** Wei Zhou, Igor A. Dmitriev, Paolo Melchiorre

**Affiliations:** † ICIQ − Institute of Chemical Research of Catalonia, Avinguda Països Catalans 16, 43007 Tarragona, Spain; ‡ Department of Industrial Chemistry ‘*Toso Montanari*’, University of Bologna, Via Piero Gobetti 85, 40129 Bologna, Italy

## Abstract

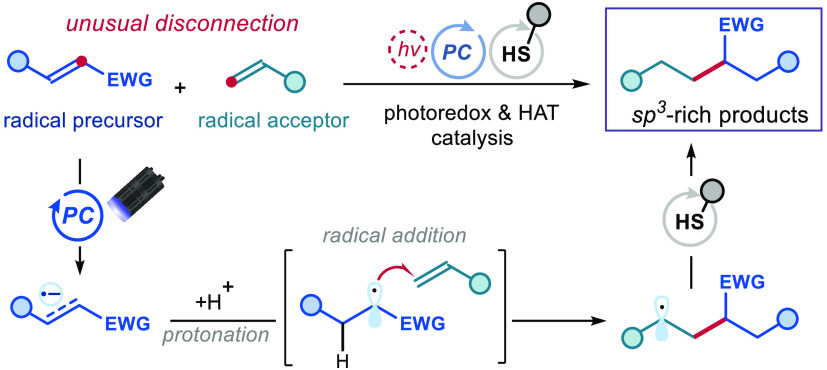

Olefins are widely available at low costs, which explains the usefulness of developing new methods for their functionalization. Here we report a simple protocol that uses a photoredox catalyst and an inexpensive thiol catalyst to stitch together two olefins, forming a new C–C bond. Specifically, an electron-poor olefin is reduced by the photoredox catalyst to generate, upon protonation, a carbon radical, which is then captured by a neutral olefin. This intermolecular cross-coupling process provides a tool for rapidly synthesizing sp^3^-dense molecules from olefins using an unconventional disconnection.

Olefins are ubiquitous motifs in petroleum-derived compounds, natural products, pharmaceuticals, and materials.^1^ Therefore, developing methods for their direct functionalization is of high synthetic significance. One useful transformation is the intermolecular cross-coupling of two olefins, as it enables the rapid formation of new C–C bonds directly from these abundant feedstocks.^2^ In this context, a reductive olefin cross-coupling has been reported^3^ recently based on a metal-hydride hydrogen atom transfer (MHAT)^4^ mechanism (Figure 1a). An iron catalyst promoted the formation of carbon radicals through MHAT activation of electron-rich olefins.^3^ These radicals were subsequently intercepted by electron-poor olefins in a classical Giese-type reaction.^5^ Overall, this process enabled the combination of two olefins to produce valuable sp^3^-hybridized products.^6^

**Figure 1:**
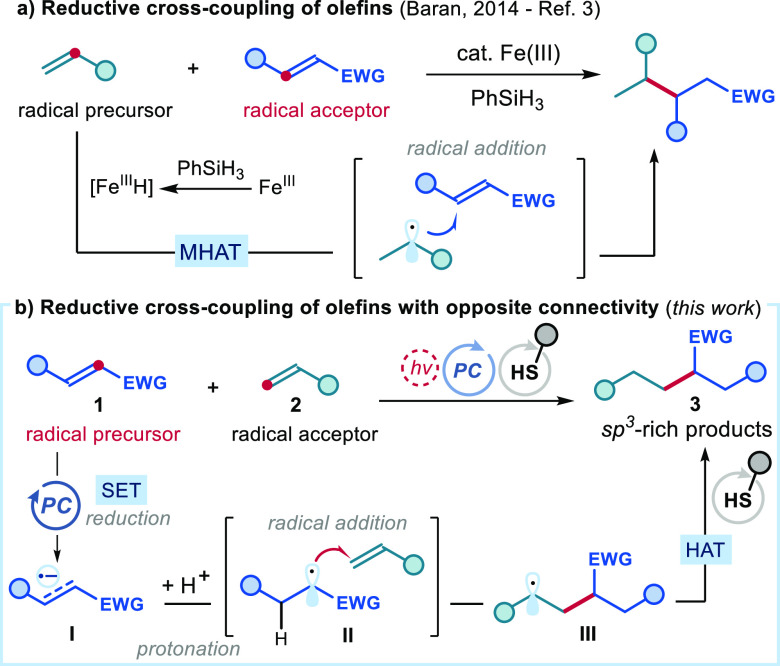
(a) Olefin cross-coupling strategy in which electron-rich olefins served as radical precursors. (b) The present strategy uses electron-poor olefins as radical precursors to afford sp^3^-rich products **3** with a different connectivity compared to the previous method. SET: single-electron transfer; HAT: hydrogen atom transfer; PC: photoredox catalyst; the purple dots indicate the substrates’ reactive sites for C–C bond formation.

Herein, we report a novel method for the intermolecular reductive cross-coupling of two olefins based on a distinct mechanistic pattern (Figure 1b). Specifically, our strategy capitalizes on the generation of radicals from electron-poor olefins **1**. We surmised that **1** could be reduced by an excited photoredox catalyst (PC) to form a radical anion **I**, which upon protonation would deliver a carbon radical **II** adjacent to an electron-withdrawing group (EWG). This electrophilic radical **II** would be favorably poised^7^ to react with an electron-rich or neutral olefin **2** via radical addition. Eventually, the ensuing radical **III** would be reduced by a thiol catalyst via a hydrogen atom transfer (HAT)^8^ path to afford the desired olefin cross-coupling product **3**. This Communication details the realization of this idea. The resulting olefin cross-coupling method complements the previously reported protocol,^3^ as the same two olefins play opposite roles (compare Figure 1a and b). As a result, sp^3^-dense products **3** with different connectivity, arising from opposite disconnections, become available.^9^

Crucial to our reaction design was the ability to reduce electron-poor olefins **1** using a light-activated photoredox catalyst. The literature offers some studies demonstrating the feasibility of selectively reducing **1** to the corresponding radical anions of type **I**.^10^ While lending support to our mechanistic hypothesis, these methods were mainly limited to the reduction of specific olefins **1** or required UV light irradiation,^10^ or the resulting intermediate **I** reacted preferentially at the anion site via a polar pathway, rather than capitalizing on radical reactivity.^11^ A few examples were reported where the radical intermediate **I** was intercepted by an olefin,^12^ but mainly in an intramolecular fashion.^13^

We started our investigations by using dimethyl fumarate **1a** [*E*(**1a**/**1a**^**–•**^) = −1.47 V vs SCE]^14^ as the radical precursor and styrene **2a** as the radical acceptor (Figure 2a). The experiments were performed in 1,2-dichloroethane (DCE) as the solvent in the presence of γ-terpinene **5a** as H donor^15^ (2.0 equiv) and thiophenol (20 mol %) as the HAT catalyst under irradiation by a blue LED (λ_max_ = 450 nm). We initially tested the highly reducing iridium-based photoredox catalysts **A** (E*[(Ir(IV)/Ir(III)*] = −1.88 V vs SCE),^16^ as it had the required redox power to activate **1a**. The cross-coupling reaction of **1a** and **2a** proceeded smoothly, affording the target product **3a** in high yield (entry 1). The use of organic photocatalyst **B** led to a drastically reduced reactivity (entry 2). The screening of other HAT catalysts **4** (entries 3, 4) and H donors **5** (entry 5) confirmed that γ-terpinene **5a** and thiophenol **4a** offered the best results. We also used a stoichiometric amount of thiophenol **4a** to avoid the need of any additional H donor (entry 6), but product **3a** was obtained in low yield because of competitive thiol–ene and sulfa-Michael processes. Control experiments established that the presence of the H donor (γ-terpinene **5a**, entry 6), the HAT catalyst (thiphenol **4a**), light, and photocatalyst **A** was essential for reactivity (entry 7). When the radical scavenger TEMPO was added under the optimal conditions (entry 8), the reactivity was completely inhibited, which was congruent with a radical path being operative.

**Figure 2:**
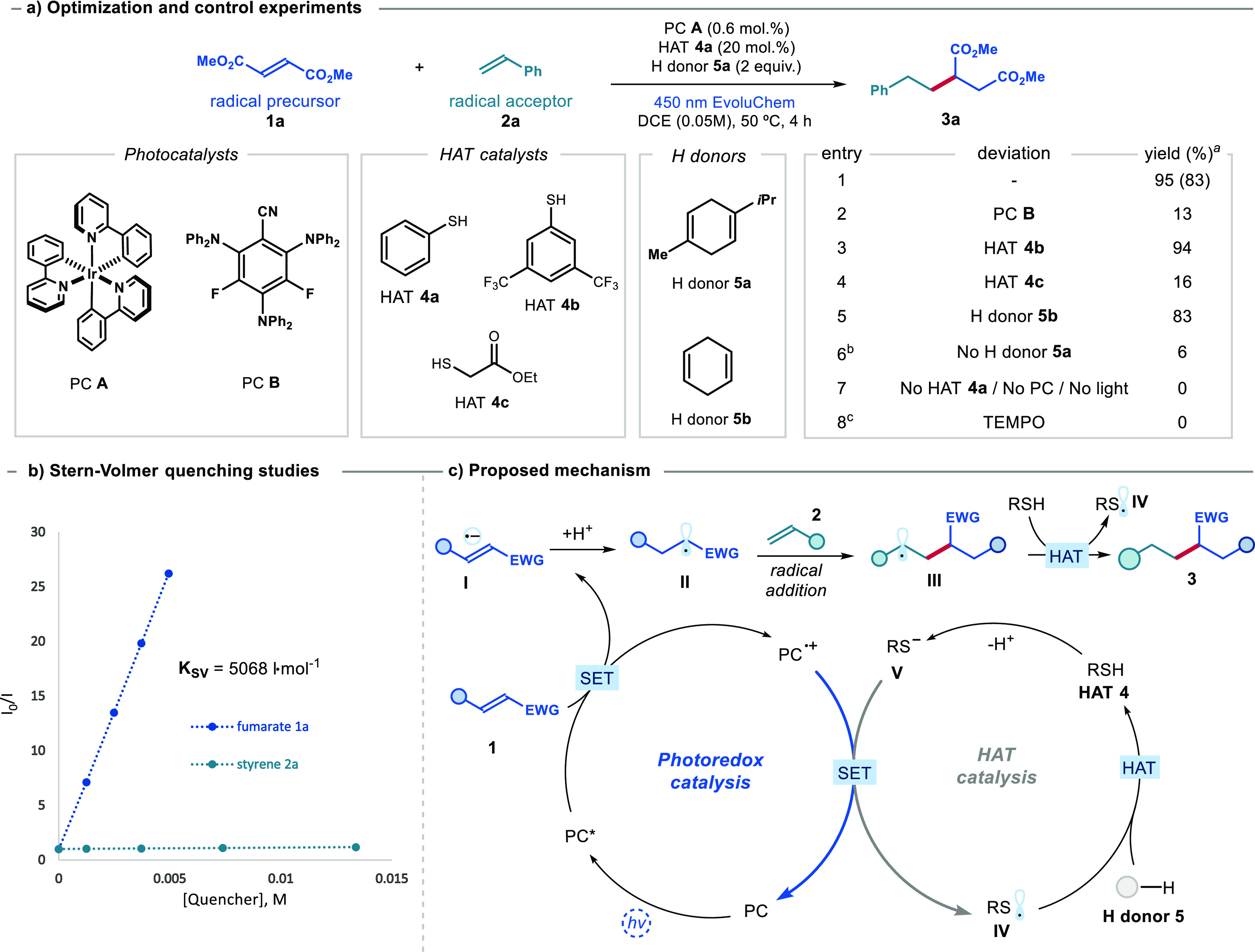
(a) Optimization studies and control experiments. Reactions performed on a 0.1 mmol scale using 1 equiv of styrene **2a** and 1.5 equiv of dimethyl fumarate **1a** under illumination by a blue LED (EvoluChem) at 450 nm. ^a^Yield of **3a** was determined by GC-FID analysis of the crude reaction mixture using 1,3,5-trimethoxybenzene as the internal standard. Yields of isolated **3a** are reported in parentheses (0.2 mmol scale). ^b^Using 2.0 equiv of thiophenol **4a**. ^c^Using 1.5 equiv of TEMPO. (b) Stern–Volmer luminescence quenching studies of Ir(ppy)_3_ (PC **A**, 1.5 μM in DCE) with increasing amounts of dimethyl fumarate **1a** and styrene **2a** (excitation wavelength = 440 nm; emission recorded at 513 nm). (c) Proposed mechanism of the reductive cross-coupling of olefins.

To gain insight into the reaction mechanism, we conducted Stern–Volmer quenching studies. Irradiating a DCE solution of photoredox catalyst **A** at 440 nm revealed emission with a maximum at 513 nm (details in Section H1 of the Supporting Information). Measurements in the presence of increasing amounts of quenchers showed that dimethyl fumarate **1a** is a much more effective quencher than styrene **2a** (Figure 2b). This observation aligns with our mechanistic proposal in which the excited photocatalyst can effectively reduce electron-poor olefin **1** via single-electron transfer (SET), leading to the reactive radical anion of type **I** (Figure 2c). The ensuing radical anion **I** would then undergo protonation to give radical **II**, which can be intercepted by olefin **2**. The resulting radical **III** could then abstract a hydrogen atom from thiol **4**, leading to reductive olefin coupling product **3** and thiyl radical **IV**. Meanwhile, thiolate **V** [*E*(**IV**/**V**) = −0.36 V vs [FC^+^/FC] in DMSO for thiophenolate]^17^ could be oxidized by the oxidized photocatalyst (*E*[Ir(IV)/Ir(III)] = 0.77 V vs SCE)^16^ to turn over the photocatalyst and generate the thiyl radical **IV**. This latter radical can abstract a hydrogen atom from H donor **5** to regenerate thiol catalyst **4**.

Using the conditions described in entry 1 of Figure 2a we then investigated the scope of the acceptor olefins using fumarate **1a** as the radical precursor (Figure 3). First, we demonstrated that the process was equally efficient on a 5 mmol scale, yielding 1.0 g of product **3a** (80% yield). Styrenes with different substitutions at the *ortho-*, *meta*-, and *para*-position afforded the olefin cross-coupling products **3b**–**3h** with good to excellent yields. Nonactivated terminal olefins bearing aliphatic substituents were suitable for this process, affording the corresponding products **3i**–**3n** in good yields. A variety of functional groups were tolerated well, including a free alcohol (adducts **3j** and **3m**), a halogen atom (product **3l**), and an aldehyde (**3n**). Electron-rich and heteroatom-functionalized olefins were also reactive, affording products **3o**–**3q**.

**Figure 3:**
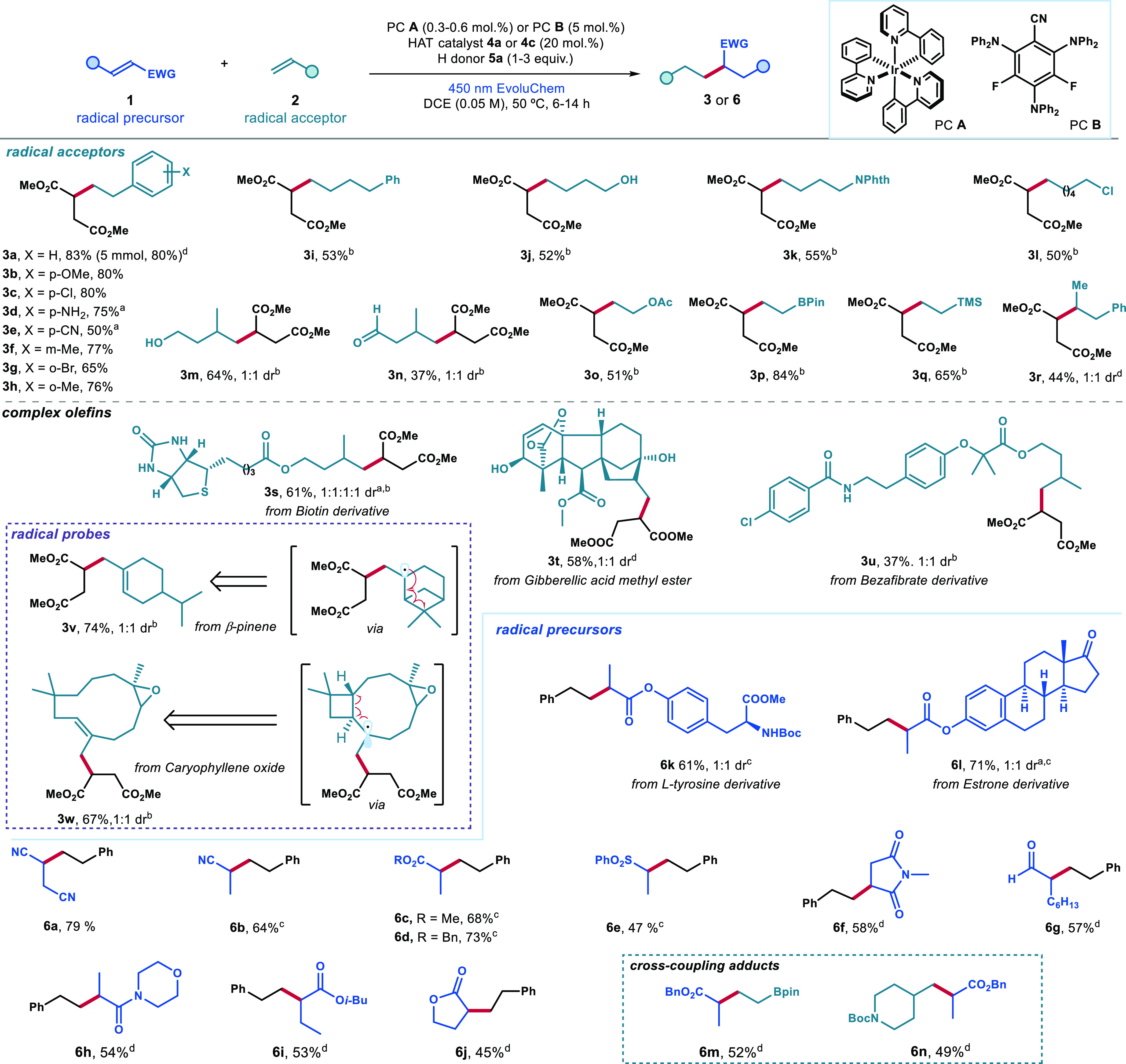
Photochemical reductive cross-coupling of olefins. Reactions performed on a 0.2 mmol scale at 50 °C using PC **A** (0.6 mol %), **4a** (20 mol %), γ-terpinene **5a** (2.0 equiv), **1** (1.5 equiv), and **2** (1.0 equiv). Yields refer to isolated products **3** and **6**. ^a^Yield was determined by ^1^H NMR analysis of the crude mixture using dibromomethane as the internal standard. ^b^Performed using PC **A** (0.3 mol %), ethyl thioglycolate **4c** (20 mol %), γ-terpinene **5a** (1 equiv), **1** (1.0 equiv), and **2** (3.0 equiv). ^c^Performed using PC **B** (5.0 mol %), PhSH **4a** (20 mol %), 2,4,6-collidine (1 equiv), γ-terpinene **5a** (3 equiv), **1** (1.0 equiv), and **2** (3.0 equiv). ^d^Reaction performed under modified conditions; see the Supporting Information for details. Phth: phthalimide; Ac: acetyl; BPin: bis(pinacolato)boron; TMS: trimethylsilyl; Ph: phenyl; Bn: benzyl; *i*-Bu: isobutyl; Boc: *tert*-butoxycarbonyl.

While terminal disubstituted olefins offered good yields (adducts **3m**,**3n**, **3s**–**3w**), internal olefins exhibited reduced reactivity. For example, *trans*-β-methylstyrene afforded product **3r** in 44% yield (see Figure S4 in the Supporting Information for a list of substrates that proved to be unreactive or poorly reactive). The higher reactivity of terminal olefins allowed us to chemoselectively functionalize a derivative of the gibberellic acid (adduct **3t**), leaving the internal double bond unaffected. Our methodology was also suitable for the late-stage functionalization of natural products and their derivatives bearing an olefin moiety. For example, a biotin and a bezafibrate derivative reacted smoothly to provide adducts **3s** and **3u**, respectively. When β-pinene or caryophyllene oxide were reacted, the ring-opening products **3v** and **3w**, respectively, were obtained in good yields. This observation supports the involvement of a radical of the type **III** (Figure 2c) as an intermediate in the process.

Next, we assessed the electron-poor olefins capable of reacting with styrene **2a** in our olefin cross-coupling protocol (Figure 3, lower panel). We found that fumaronitrile could serve as a radical precursor, yielding product **6a** in a high yield. By using photocatalyst **B** in combination with a weak base (2,4,6-collidine, 1 equiv), we achieved enhanced reactivity with less activated electron-poor olefins. Under these slightly modified conditions (refer to Section H4 in the Supporting Information for the proposed mechanism), acrylonitrile, acrylates, and vinylsulfone underwent smooth reactions, yielding the corresponding products **6b**–**6e** in moderate to good yields. Additionally, other electron-poor olefins, such as *N*-methyl maleimide, an enal, and an acrylamide, also generated olefin cross-coupling products **6f**–**6h** in decent yields. The protocol was also effective for internal and cyclic acrylates, affording adducts **6i** and **6j**. Furthermore, acrylates derived from biorelevant molecules successfully participated in our coupling method (products **6k** and **6l**). To demonstrate the versatility of the methodology, we investigated cross-coupling reactions between two less activated substrates, leading to the formation of adducts **6m** and **6n** in moderate yield. In general, the main byproduct of this protocol is the reduction product of electron-poor olefins **1**, the formation mechanism of which is discussed in Section G2 of the Supporting Information.

We then conducted further mechanistic investigations using benzyl acrylate. First, a radical probe experiment was performed with cyclopropyl-containing styrene **7**, resulting in the formation of the ring-opening product **8** in good yield (Figure 4a). This outcome suggested the generation of a radical from the electron-poor olefin, followed by its trapping by styrene **7**.^18^ When D_2_O was introduced into the reaction, the deuterated product **6d-D** was obtained in 52% yield (Figure 4b). NMR analysis revealed 70% deuterium incorporation at the β position of the acrylate moiety and 30% incorporation at the benzylic position. This finding is consonant with the SET reduction of the electron-poor olefin, forming the radical anion of type **I**. Protonation then occurs at the β carbon,^10c^ leading to the formation of the more stabilized radical **II**. Radical addition to styrene then forms radical intermediate **III**, which can be deuterated through HAT with deuterated thiophenol, which can be generated *in situ* via D_2_O exchange.

**Figure 4:**
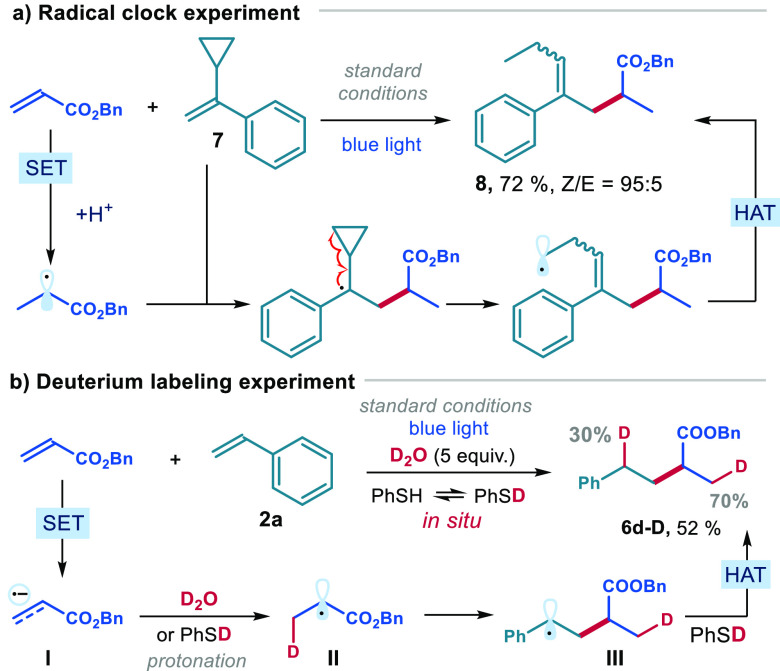
(a) Radical clock experiment. (b) Deuterium labeling experiment. Reactions performed on a 0.2 mmol scale using PC **B** (5.0 mol %), PhSH **4a** (20 mol %), 2,4,6-collidine (1 equiv), γ-terpinene **5a** (3 equiv), and 3 equiv of **7** or **2a** in DCE (0.05 M) for 14 h.

In summary, we have developed a light-driven protocol that uses mild conditions and readily available catalysts for the reductive cross-coupling of olefins, providing sp^3^-rich products with distinct connectivity. Central to this transformation was the photoredox-enabled generation of radicals through the reduction of electron-poor olefins. These substrates were readily coupled with various styrenes and other readily available neutral olefins. A notable feature of this method is its high tolerance for functional groups, which we harnessed for the late-stage modification of biorelevant compounds.

## Supporting Information Available

Details of experimental procedures and full characterization data and copies of NMR spectra (PDF)
