# Supplementary material for: Reductive Cross-Coupling of Olefins via a Radical Pathway
Source: J Am Chem Soc. 2023 Nov 10;145(46):25098–102. doi: 10.1021/jacs.3c11285 (PMC10682986; doi:10.1021/jacs.3c11285)

Supporting Information

Reductive Cross-Coupling of Olefins via a Radical Pathway

Wei Zhou,[†,#] Igor A. Dmitriev,[†,#] and Paolo Melchiorre[‡]*

[†]*ICIQ* – Institute of Chemical Research of Catalonia, Avinguda Països Catalans 16, 43007 Tarragona, Spain

[‡]University of Bologna, Department of Industrial Chemistry '*Toso Montanari*', via Piero Gobetti 85, 40129 Bologna, Italy

[#] These authors contributed equally to this work

*Correspondence to: p.melchiorre@unibo.it

A. General Information 3

B. Synthesis of the Substrates 4

C. Experimental Setup 5

D. General Procedure 6

E. Characterization of Products 7

F. Reaction Scale-Up 23

G. Additional experimental information 24

G1. Unsuccessful substrates 24

G2. Major byproducts 24

G3. Optimization of equivalents 25

H. Mechanistic Experiments 25

H1. Stern-Volmer Quenching Studies 25

H2. Radical clock experiment 27

H3. Deuterium labeling experiment 28

H4. Mechanistic rationalization for less activated radical precursors 32

I. References 33

J. NMR spectra 35

A. General Information

The NMR spectra were recorded at 400 MHz and 500 MHz for ^{1}H, 101 or 126 MHz for ^{13}C. The chemical shift (δ) for ^{1}H and ^{13}C are given in ppm relative to residual signals of the solvents ($CHCl_3$ @ 7.26 ppm ^{1}H NMR, $CDCl_3$ @ 77.16 ppm ^{13}C NMR, and tetramethylsilane @ 0 ppm). Coupling constants are given in Hertz. The following abbreviations are used to indicate the multiplicity: s, singlet; d, doublet; t, triplet; q, quartet; m, multiplet; br. s., broad singlet; app, apparent.

High resolution mass spectra (HRMS) were obtained from the ICIQ HRMS unit on MicroTOF Focus and Maxis Impact (Bruker Daltonics) with electrospray ionization (ESI).

UV-vis measurements were carried out on a Shimadzu UV-2401PC spectrophotometer equipped with photomultiplier detector, double beam optics and D_2 and W light sources or an Agilent Cary60 spectrophotometer. Yields refer to isolated materials of >95% purity as determined by ^{1}H NMR analysis. The team of the Research Support Area at ICIQ, particularly the NMR and the High-Resolution Mass Spectrometry Units, is thanked for their support.

General Procedures. All reactions were set up under an argon atmosphere in oven-dried glassware. Synthesis grade solvents were used as purchased; anhydrous solvents were taken from a commercial SPS solvent dispenser. Chromatographic purification of products was accomplished using forced-flow chromatography (FC) on silica gel (230-400 mesh). For thin layer chromatography (TLC) analysis throughout this work, Merck pre-coated TLC plates (silica gel 60 GF_{254}, 0.25 mm) were employed, using UV light as the visualizing agent and an acidic mixture of vanillin, basic aqueous potassium permanganate ($KMnO_4$) or phosphomolybdic acid (PMA, ethanol solution) as stain solutions, and heat as developing agents. Organic solutions were concentrated under reduced pressure on a Büchi rotatory evaporator at 40°C.

Materials. Most of the starting materials used in this study are commercial and were purchased at the highest purity available from Sigma-Aldrich, Fluka, Alfa Aesar, Fluorochem, TCI, and used as received, without further purifications, unless otherwise stated.

B. Synthesis of the Substrates

Photocatalyst **B** (3DPA2FBN),[1] 4,5-dimethylcyclohexa-1,4-diene-1-carboxylate,[2] 3-methyl-3-butenal,[3] and acrylates **1k**[4] and **1l**[5] were synthesized according to reported procedures.

Below, the procedures for the substrates that we have synthesized are detailed.

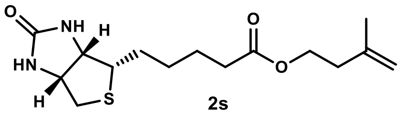

2s

3-Methylbut-3-en-1-yl 5-((3aS,4S,6aR)-2-oxohexahydro-1H-thieno[3,4-d]imidazol-4-yl)pentanoate (2s): Synthesized according to a modified procedure.[6] To an oven-dred 100 mL round-bottom flask containing a magnetic stir-bar, Biotin (1.05 g, 4.3 mmol), DMAP (269 mg, 2.2 mmol) and 1-ethyl-3-(3-dimethylaminopropyl)carbodiimide hydrochloride (1.26 g, 6.6 mmol) were added. The flask was sealed with a septum and filled with argon. Dry DMF (50 ml) and 3-methylbut-3-en-1-ol (0.53 ml, 5.2 mmol) were added subsequently and the stirring was switched on. The flask was placed onto a 40 °C bath and kept overnight. The solvent was evaporated, the material dried on high vacuum and then purified via silica gel column chromatography (DCM:MeOH = 30/1) to afford the product as a white solid (1.05 g, 78% yield).

^{1}H NMR (500 MHz, $CDCl_3$) δ 5.92 (s, 1H), 5.50 (s, 1H), 4.80 – 4.78 (m, 1H), 4.73 – 4.71 (m, 1H), 4.49 (ddt, *J* = 7.8, 5.0, 1.2 Hz, 1H), 4.30 (ddd, *J* = 7.8, 4.4, 1.5 Hz, 1H), 4.18 (t, *J* = 6.9 Hz, 2H), 3.14 (ddd, *J* = 8.5, 6.5, 4.6 Hz, 1H), 2.90 (dd, *J* = 12.8, 5.0 Hz, 1H), 2.73 (d, *J* = 12.8 Hz, 1H), 2.35 – 2.28 (m, 4H), 1.74 (ddt, *J* = 1.4, 0.9, 0.5 Hz, 3H), 1.73 – 1.59 (m, 4H), 1.51 – 1.36 (m, 2H).

^{13}C NMR (126 MHz, $CDCl_3$) δ 173.8, 163.8, 141.8, 112.4, 62.6, 62.1, 60.2, 55.6, 40.7, 36.8, 34.0, 28.5, 28.4, 24.9, 22.6.

HRMS: (ESI$^+$) calculated for $C_{15}H_{24}N_2NaO_3S^+$ [M+Na$^+$]: 335.1400, found 335.1401.

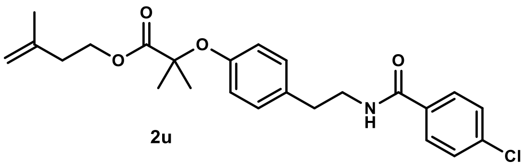

2u

3-Methylbut-3-en-1-yl 5-((3aS,4S,6aR)-2-oxohexahydro-1H-thieno[3,4-d]imidazol-4-yl)pentanoate (2u): Synthesized according to a modified procedure.[6] To an oven-dred 100 mL round-bottom flask containing a magnetic stir-bar, Bezafibrate (778 mg, 2.15 mmol), DMAP (135 mg, 1.1 mmol) and EDCI hydrochloride (633 mg, 3.3 mmol) were added. The flask was sealed with a septum and filled with argon. Dry DMF (25 ml) and 3-methylbut-3-en-1-ol (263 µL, 2.6 mmol) were added subsequently and the stirring was switched on. The flask was placed onto a 40°C bath and kept for 5 hours. The solvent was evaporated, the material dried on high vacuum and then purified via silica gel column chromatography (DCM:MeOH = 30/1) to afford the product as a white solid (352 mg, 38% yield).

^{1}H NMR (500 MHz, $CDCl_3$) δ 7.63 – 7.58 (m, 2H), 7.40 – 7.35 (m, 2H), 7.10 – 7.05 (m, 2H), 6.83 – 6.78 (m, 2H), 6.07 (t, *J* = 5.8 Hz, 1H), 4.79 – 4.75 (m, 1H), 4.71 – 4.67 (m, 1H), 4.28 (t, *J* = 6.8 Hz, 2H), 3.66 (td, *J* = 7.0, 5.8 Hz, 2H), 2.85 (t, *J* = 7.0 Hz, 2H), 2.34 (t, *J* = 6.8 Hz, 2H), 1.73 – 1.71 (m, 3H), 1.57 (s, 6H).

^{13}C NMR (126 MHz, $CDCl_3$) δ 174.3, 166.5, 154.3, 141.4, 137.8, 133.1, 132.5, 129.6, 129.0, 128.4, 119.8, 112.7, 79.3, 63.6, 41.4, 36.7, 34.9, 25.6, 22.5.

HRMS: (ESI^+) calculated for $C_{24}H_{28}ClNNaO_4^+$ [$M+Na^+$]: 452.1599, found 452.1610.

C. Experimental Setup

All reactions were performed using an *EvoluChem*™ P303-30-1 LEDs (18 W, λ_{max} = 450 nm, 1 cm away, Figure S1). A fan was used to cool down the reactor. Using a thermometer, the temperature inside the reaction vessel was measured to be between 45-55 ºC under the given reaction conditions.

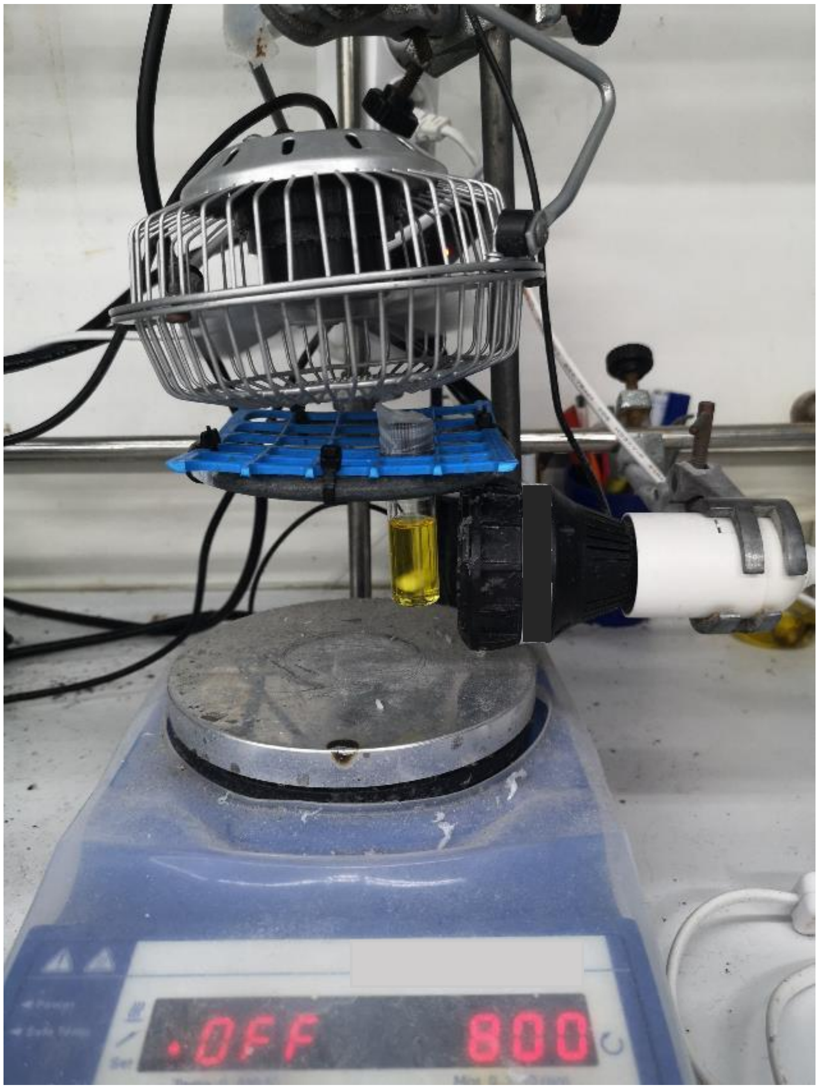

Figure S1. Photoreactor setup.

D. General Procedure

Stock solution preparation of fac-Ir(ppy)$_3$: To an oven-dried 8 mL vial, 4.0 mg of *fac*-Ir(ppy)$_3$ were added. The vial was sealed with a septum, evacuated and backfilled with argon three times, and 4.0 mL of degassed DCE were added. The resulting suspension was sonicated for 10-15 minutes until complete dissolution and <u>*used the same day as prepared*</u>.

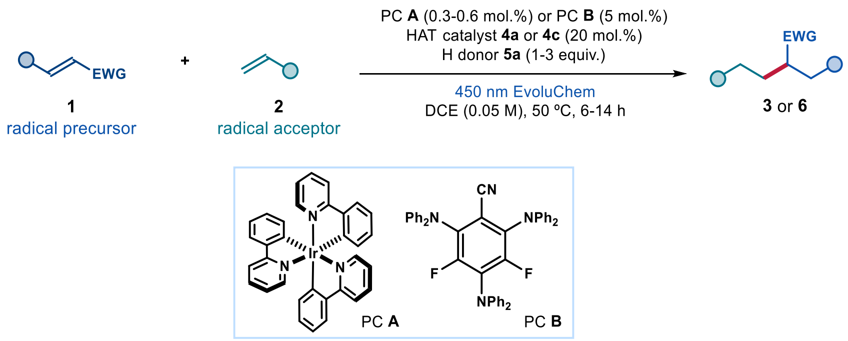

Figure S2. General procedure.

<u>**Procedure.**</u> To an oven dried 8 mL vial containing a dry Teflon stir bar, the photocatalyst (if 3DPA2FBN), the olefin substrates **1** and **2** (if solid) and bases (if solid and needed) were added. The vial was sealed with a Teflon septum screw cap, evacuated and backfilled with argon three times, and the solvent was added. Then the photocatalyst stock solution (if *fac*-Ir(ppy)$_3$), olefin substrates **1** and **2** (if liquid), H donor, HAT catalyst, and bases (if liquid and needed) were added sequentially. The amount of added solvent was such that the total amount of solvent (added + stock solution, if used) was 4.0 mL. The solvent used was DCE sparged with nitrogen for 15 minutes, unless otherwise stated.

The vial was then placed in the photoreactor (Figure S1) and irradiated under stirring for 6 hours (if radical acceptors are styrenes) or 14 hours, unless otherwise specified, at 50 °C. Then the solvent was evaporated and the crude mixture purified by flash column chromatography on silica gel to furnish the target product **3** or **6**.

E. Characterization of Products

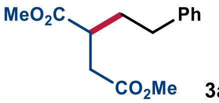
3a

Dimethyl 2-phenethylsuccinate (3a): Synthesized according to the General Procedure using *fac*-Ir(ppy)$_3$ (800 µL stock solution, 0.6 mol%), PhSH (4.0 µL, 0.04 mmol, 20 mol%), γ-terpinene (64.0 µL, 0.4 mmol, 2 equiv.), dimethyl fumarate **1a** (43.0 mg, 0.3 mmol, 1.5 equiv.) and styrene **2a** (23.0 µL, 0.2 mmol, 1 equiv.). Upon 6 hours stirring, the crude mixture was purified by flash column chromatography on silica gel (10% EtOAc in hexanes as eluent) to afford **3a** (41.5 mg, 83% yield) as a light-yellow oil.

^{1}H NMR (500 MHz, CDCl$_3$) δ 7.30 – 7.26 (m, 2H), 7.21 – 7.17 (m, 2H), 7.17 – 7.16 (m, 1H), 3.71 (s, 3H), 3.67 (s, 3H), 2.94 – 2.87 (m, 1H), 2.77 (dd, *J* = 16.5, 9.1 Hz, 1H), 2.69 – 2.58 (m, 2H), 2.49 (dd, *J* = 16.5, 5.3 Hz, 1H), 2.00 (dddd, *J* = 13.7, 9.4, 7.8, 6.5 Hz, 1H), 1.83 (dddd, *J* = 13.7, 9.4, 6.8, 6.0 Hz, 1H). ^{13}C NMR (126 MHz, CDCl$_3$) δ 175.3, 172.3, 141.2, 128.6, 128.5, 126.2, 52.0, 51.9, 40.9, 36.0, 33.7, 33.3.

Matching reported literature data.[7]

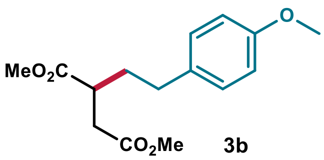
3b

Dimethyl 2-(4-methoxyphenethyl)succinate (3b): Synthesized according to the General Procedure using *fac*-Ir(ppy)$_3$ (800 µL stock solution, 0.6 mol%), PhSH (4.1 µL, 0.04 mmol, 20 mol%), γ-terpinene (64.0 µL, 0.4 mmol, 2 equiv.), dimethyl fumarate **1a** (43.0 mg, 0.3 mmol, 1.5 equiv.) and 4-methoxystyrene **2b** (26.5 µL, 0.2 mmol, 1 equiv.). Upon 6 hours stirring, the crude mixture was purified by flash column chromatography on silica gel (10% EtOAc in hexanes as eluent) to afford **3b** (45.0 mg, 80% yield) as a light-yellow oil.

^{1}H NMR (500 MHz, CDCl$_3$) δ 7.10 – 7.06 (m, 2H), 6.84 – 6.80 (m, 2H), 3.78 (s, 3H), 3.70 (s, 3H), 3.66 (s, 3H), 2.87 (dddd, *J* = 9.2, 7.7, 6.0, 5.2 Hz, 1H), 2.75 (dd, *J* = 16.5, 9.2 Hz, 1H), 2.62 – 2.52 (m, 2H), 2.47 (dd, *J* = 16.5, 5.2 Hz, 1H), 1.95 (dddd, *J* = 13.7, 9.2, 7.8, 6.7 Hz, 1H), 1.78 (dddd, *J* = 13.7, 9.2, 6.9, 6.0 Hz, 1H).

^{13}C NMR (126 MHz, CDCl$_3$) δ 175.3, 172.4, 158.1, 133.2, 129.4, 114.0, 55.4, 52.0, 51.9, 40.8, 36.0, 33.9, 32.4. HRMS: (ESI$^+$) calculated for $C_{15}H_{20}NaO_5^+$ [M+Na$^+$]: 303.1203, found 303.1210.

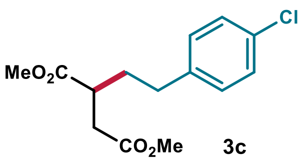
3c

Dimethyl 2-(4-chlorophenethyl)succinate (3c): Synthesized according to General Procedure using *fac*-Ir(ppy)$_3$ (800 µL stock solution, 0.6 mol%), PhSH (4.1 µL, 0.04 mmol, 20 mol%), γ-terpinene (64.0 µL, 0.4 mmol, 2 equiv.), dimethyl fumarate **1a** (43.0 mg, 0.3 mmol, 1.5 equiv.) and 4-chlorostyrene **2c** (25.5 µL, 0.2 mmol, 1 equiv.). Upon 6 hours stirring, the crude mixture was purified by flash column chromatography on silica gel (9% to 11% EtOAc in hexanes as eluent) to afford **3c** (45.5 mg, 80% yield) as a colorless oil.

^{1}H NMR (500 MHz, CDCl$_3$) δ 7.26 – 7.22 (m, 2H), 7.11 – 7.08 (m, 2H), 3.70 (s, 3H), 3.66 (s, 3H), 2.86 (ddt, *J* = 8.9, 7.9, 5.6 Hz, 1H), 2.75 (dd, *J* = 16.5, 8.9 Hz, 1H), 2.66 – 2.53 (m, 2H), 2.47 (dd, *J*

= 16.5, 5.4 Hz, 1H), 1.96 (dddd, J = 13.7, 9.4, 8.0, 6.3 Hz, 1H), 1.79 (dddd, J = 13.7, 9.5, 6.8, 5.8 Hz, 1H).

^{13}C NMR (126 MHz, $CDCl_3$) δ 175.1, 172.2, 139.6, 132.0, 129.9, 128.7, 52.1, 52.0, 40.8, 36.0, 33.5, 32.7. HRMS: (ESI$^+$) calculated for $C_{14}H_{17}ClNaO_4^+$ [M+Na$^+$]: 307.0708, found 307.0711.

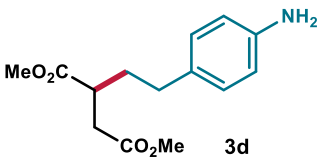
3d

Dimethyl 2-(4-aminophenethyl)succinate (3d): Synthesized according to General Procedure using *fac*-Ir(ppy)$_3$ (800 µL stock solution, 0.6 mol%), PhSH (4.1 µL, 0.04 mmol, 20 mol%), γ-terpinene (64.0 µL, 0.4 mmol, 2 equiv.), dimethyl fumarate **1a** (43.0 mg, 0.3 mmol, 1.5 equiv.) and 4-aminostyrene **2d (**23.5 µL, 0.2 mmol, 1 equiv.). Upon 6 hours stirring, ^{1}H NMR analysis (using CH_2Br_2 as internal standard) revealed 75% yield of **3d**. The crude mixture was purified by flash column chromatography on silica gel (25% to 33% EtOAc in hexanes as eluent) to afford **3d** (35.5 mg, 67%, ~90% purity) as an orange oil. An analytical sample was obtained via preparative HPLC (column: ChiralPak IC 250x4.6 mm, 5µm; mobile phase: Hex/EtOH 85:15; flow: 1 ml/min; wavelength: 254 nm).

^{1}H NMR (500 MHz, $CDCl_3$) δ 6.98 – 6.93 (m, 2H), 6.64 – 6.60 (m, 2H), 3.70 (s, 3H), 3.66 (s, 3H), 3.56 (br. s, 2H), 2.87 (dddd, J = 9.2, 7.6, 6.1, 5.1 Hz, 1H), 2.74 (dd, J = 16.5, 9.2 Hz, 1H), 2.51 (ddd, J = 9.3, 6.7, 2.9 Hz, 2H), 2.47 (dd, J = 16.5, 5.1 Hz, 1H), 1.93 (dddd, J = 13.6, 9.1, 7.6, 6.7 Hz, 1H), 1.81 – 1.71 (m, 1H).

^{13}C NMR (126 MHz, $CDCl_3$) δ 175.4, 172.5, 144.6, 131.2, 129.3, 115.4, 52.0, 51.9, 40.9, 36.0, 34.0, 32.5. HRMS: (ESI$^+$) calculated for $C_{14}H_{19}NNaO_4^+$ [M+Na$^+$]: 288.1206, found 288.1211.

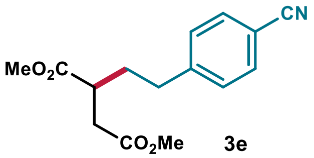
3e

Dimethyl-2-(4-cyanophenethyl)succinate (3e): Synthesized according to the General Procedure using *fac*-Ir(ppy)$_3$ (800 µL stock solution, 0.6 mol%), PhSH (4.1 µL, 0.04 mmol, 20 mol%), γ-terpinene (64.0 µL, 0.4 mmol, 2 equiv.), dimethyl fumarate **1a** (43.0 mg, 0.3 mmol, 1.5 equiv.) and 4-cyanostyrene **2e** (25.1 µL, 0.2 mmol, 1 equiv.). Upon 6 hours stirring, ^{1}H NMR analysis (using CH_2Br_2 as internal standard) revealed 50% yield of **3e**. The crude mixture was purified by flash column chromatography on silica gel (12% EtOAc in hexanes as eluent) to afford **3e** (26.7 mg, 48% yield, ~90% purity) as a light-yellow oil. An analytical sample was obtained via preparative HPLC (column: XSelect CSH Fluoro-Phenyl 150x4.6 mm, 3.5µm; mobile phase: H2O/MeOH 50:50; flow: 1 ml/min; wavelength: 254 nm).

^{1}H NMR (500 MHz, $CDCl_3$) δ 7.60 – 7.56 (m, 2H), 7.31 – 7.27 (m, 2H), 3.72 (s, 3H), 3.68 (s, 3H), 2.87 (tt, J = 8.3, 5.6 Hz, 1H), 2.76 (dd, J = 16.5, 8.6 Hz, 1H), 2.73 – 2.64 (m, 2H), 2.48 (dd, J = 16.5, 5.7 Hz, 1H), 1.99 (dddd, J = 14.1, 9.7, 8.2, 6.1 Hz, 1H), 1.83 (dddd, J = 13.7, 9.8, 6.6, 5.5 Hz, 1H).

^{13}C NMR (126 MHz, $CDCl_3$) δ 174.9, 172.1, 146.8, 132.5, 129.4, 119.1, 110.3, 52.2, 52.1, 40.8, 36.1, 33.5, 33.0. HRMS: (ESI^+) calculated for $C_{15}H_{17}NNaO_4^+$ [M+Na$^+$]: 298.1050, found 298.1059.

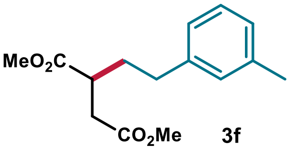

3f

Dimethyl 2-(3-methylphenethyl)succinate (3f): Synthesized according to General Procedure using *fac*-Ir(ppy)$_3$ (800 µL stock solution, 0.6 mol%), PhSH (4.1 µL, 0.04 mmol, 20 mol%), γ-terpinene (64.0 µL, 0.4 mmol, 2 equiv.), dimethyl fumarate **1a** (43.0 mg, 0.3 mmol, 1.5 equiv.) and 3-methylstyrene **2f** (26.5 µL, 0.2 mmol, 1 equiv.). Upon 6 hours stirring, the crude mixture was purified by flash column chromatography on silica gel (6% to 9% EtOAc in hexanes as eluent) to afford **3f** (40.5 mg, 77% yield) as a yellowish oil.

^{1}H NMR (500 MHz, $CDCl_3$) δ 7.19 – 7.15 (m, 1H), 7.03 – 6.95 (m, 3H), 3.72 (s, 3H), 3.67 (s, 3H), 2.93 – 2.87 (m, 1H), 2.77 (dd, J = 16.5, 9.2 Hz, 1H), 2.65 – 2.54 (m, 2H), 2.49 (dd, J = 16.5, 5.2 Hz, 1H), 2.33 (s, 3H), 1.98 (dddd, J = 13.7, 9.5, 7.7, 6.6 Hz, 1H), 1.82 (dddd, J = 13.7, 9.5, 6.8, 6.0 Hz, 1H). ^{13}C NMR (126 MHz, $CDCl_3$) δ 175.3, 172.4, 141.1, 138.1, 129.3, 128.5, 127.0, 125.5, 52.0, 51.9, 41.0, 36.0, 33.8, 33.3, 21.5.

HRMS: (ESI^+) calculated for $C_{15}H_{20}NaO_4^+$ [M+Na$^+$]: 287.1254, found 287.1264.

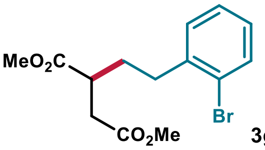

3g

Dimethyl 2-(2-bromophenethyl)succinate (3g): Synthesized according to General Procedure using *fac*-Ir(ppy)$_3$ (800 µL stock solution, 0.6 mol%), PhSH (4.1 µL, 0.04 mmol, 20 mol%), γ-terpinene (64.0 µL, 0.4 mmol, 2 equiv.), dimethyl fumarate **1a** (43.0 mg, 0.3 mmol, 1.5 equiv.) and 2-bromostyrene **2g** (25.0 µL, 0.2 mmol, 1 equiv.). Upon 6 hours stirring, the crude mixture was purified by flash column chromatography on silica gel (8% EtOAc in hexanes as eluent) to afford **3g** (43.0 mg, 65% yield) as a yellowish oil.

^{1}H NMR (500 MHz, $CDCl_3$) δ 7.52 – 7.50 (m, 1H), 7.25 – 7.19 (m, 2H), 7.08 – 7.03 (m, 1H), 3.73 (s, 3H), 3.68 (s, 3H), 2.97 – 2.91 (m, 1H), 2.80 (dd, J = 16.6, 9.2 Hz, 1H), 2.79 – 2.69 (m, 2H), 2.53 (dd, J = 16.6, 5.3 Hz, 1H), 1.95 (dddd, J = 13.6, 10.3, 7.5, 6.1 Hz, 1H), 1.88 – 1.80 (m, 1H).

^{13}C NMR (126 MHz, $CDCl_3$) δ 175.1, 172.4, 140.5, 133.0, 130.5, 128.0, 127.7, 124.4, 52.1, 52.0, 41.0, 35.9, 33.7, 32.1.

HRMS: (ESI^+) calculated for $C_{14}H_{17}BrNaO_4^+$ [M+Na$^+$]: 351.0202, found 351.0214.

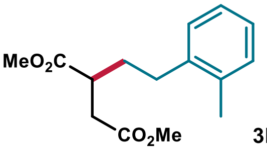

3h

Dimethyl 2-(2-methylphenethyl)succinate (3h): Synthesized according to General Procedure using *fac*-Ir(ppy)$_3$ (800 µL stock solution, 0.6 mol%), PhSH (4.1 µL, 0.04 mmol, 20 mol%), γ-terpinene (64.0 µL, 0.4 mmol, 2 equiv.), dimethyl fumarate **1a** (43.0 mg, 0.3 mmol, 1.5 equiv.) and 2-methylstyrene **2h** (26.0 µL, 0.2 mmol, 1 equiv.). Upon 6 hours stirring, the crude mixture was purified

by flash column chromatography on silica gel (6% to 9% EtOAc in hexanes as eluent) to afford **3h** (40.0 mg, 76% yield) as a colorless oil.

^{1}H NMR (500 MHz, $CDCl_3$) δ 7.15 – 7.09 (m, 4H), 3.73 (s, 3H), 3.68 (s, 3H), 2.95 (ddt, *J* = 8.9, 7.7, 5.6 Hz, 1H), 2.80 (dd, *J* = 16.5, 8.9 Hz, 1H), 2.68 – 2.54 (m, 2H), 2.51 (dd, *J* = 16.6, 5.5 Hz, 1H), 2.29 (s, 3H), 1.92 (dddd, *J* = 13.6, 10.6, 7.7, 6.1 Hz, 1H), 1.84 – 1.73 (m, 1H).

^{13}C NMR (126 MHz, $CDCl_3$) δ 175.2, 172.4, 139.4, 135.9, 130.4, 128.9, 126.4, 126.2, 52.0, 51.9, 41.3, 36.0, 32.5, 30.8, 19.2.

HRMS: (ESI^+) calculated for $C_{15}H_{20}NaO_4^+$ [$M+Na^+$]: 287.1254, found 287.1256.

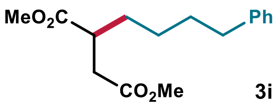

3i

Dimethyl-2-(4-phenylbutyl)succinate (3i): Synthesized according to General Procedure using *fac*-Ir(ppy)$_3$ (400 µL stock solution, 0.3 mol%), HAT **4c** (ethyl thioglycolate, 4.4 µL, 0.04 mmol, 20 mol%), γ-terpinene (32.0 µL, 0.2 mmol, 1 equiv.), dimethyl fumarate **1a** (28.8 mg, 0.2 mmol, 1 equiv.) and 4-phenyl-1-butene **2i** (90.0 µL, 0.6 mmol, 3 equiv.). Upon 14 hours stirring, the crude mixture was purified by flash column chromatography on silica gel (3% EtOAc in hexanes as eluent) to afford **3i** (29.5 mg, 53 % yield) as a colorless oil.

^{1}H NMR (400 MHz, $CDCl_3$) δ 7.33 – 7.26 (m, 2H), 7.22 – 7.15 (m, 3H), 3.72 (s, 3H), 3.69 (s, 3H)2.91 – 2.81 (m, 1H), 2.73 (dd, *J* = 16.5, 9.3 Hz, 1H), 2.66 – 2.58 (m, 2H), 2.44 (dd, *J* = 16.5, 5.1 Hz, 1H), 1.73 – 1.56 (m, 4H), 1.43 – 1.30 (m, 2H).

^{13}C NMR (101 MHz, $CDCl_3$) δ 175.6, 172.6, 142.4, 128.5, 128.4, 125.9, 51.9, 51.9, 41.2, 35.9, 35.7, 31.8, 31.2, 26.6.

HRMS: (ESI^+) calculated for $C_{16}H_{22}NaO_4^+$ [$M+Na^+$]: 301.1410, found 301.1423.

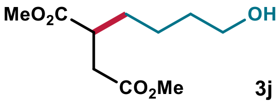

3j

Dimethyl-2-(4-hydroxybutyl)succinate (3j): Synthesized according to General Procedure using *fac*-Ir(ppy)$_3$ (400 µL stock solution, 0.3 mol%), HAT **4c** (ethyl thioglycolate, 4.4 µL, 0.04 mmol, 20 mol%), γ-terpinene (32.0 µL, 0.2 mmol, 1 equiv.), dimethyl fumarate **1a** (28.8 mg, 0.2 mmol, 1 equiv.) and 3-buten-1-ol **2j** (51.5 µL, 0.6 mmol, 3 equiv.). Upon 14 hours stirring, the crude mixture was purified by flash column chromatography on silica gel (50% EtOAc in hexanes as eluent) to afford **3j** (22.5 mg, 52 % yield) as a colorless oil.

^{1}H NMR (400 MHz, $CDCl_3$) δ 3.72 (s, 3H), 3.69 (s, 3H), 3.65 (t, *J* = 6.4 Hz, 2H), 2.93 – 2.84 (m, 1H), 2.74 (dd, *J* = 16.5, 9.1 Hz, 1H), 2.49 (d, *J* = 5.3 Hz, 1H), 1.78 – 1.65 (m, 2H), 1.58 (dtdd, *J* = 14.7, 6.0, 4.7, 2.1 Hz, 3H), 1.46 – 1.41 (m, 1H).

^{13}C NMR (101 MHz, $CDCl_3$) δ 175.4, 172.5, 62.6, 52.0, 51.9, 41.2, 35.9, 32.5, 31.7, 23.3.

HRMS: (ESI^+) calculated for $C_{10}H_{18}NaO_5^+$ [$M+Na^+$]: 241.1046, found 241.1043.

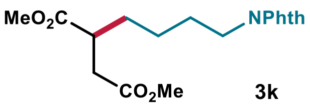

3k

Dimethyl-2-(4-(1,3-dioxoisoindolin-2-yl)butyl)succinate (3k): Synthesized according to General Procedure using *fac*-Ir(ppy)$_3$ (400 µL stock solution, 0.3 mol%), HAT **4c** (ethyl thioglycolate, 4.4 µL, 0.04 mmol, 20 mol%), γ-terpinene (32.0 µL, 0.2 mmol, 1 equiv.), dimethyl fumarate **1a** (28.8 mg, 0.2 mmol, 1 equiv.) and 2-(but-3-en-1-yl)isoindoline-1,3-dione **2k** (120.5 mg, 0.6 mmol, 3 equiv.). Upon 14 hours stirring, the crude mixture was purified by flash column chromatography on silica gel (40% EtOAc in hexanes as eluent) to afford **3k** (38.0 mg, 55 % yield) as a white solid.

^{1}H NMR (500 MHz, $CDCl_3$) δ 7.83 (dd, *J* = 5.4, 3.0 Hz, 2H), 7.71 (dd, *J* = 5.5, 3.0 Hz, 2H), 3.68 – 3.65 (m, 8H), 2.83 (dddd, *J* = 9.2, 7.5, 6.2, 5.2 Hz, 1H), 2.71 (dd, *J* = 16.6, 9.1 Hz, 1H), 2.42 (dd, *J* = 16.6, 5.2 Hz, 1H), 1.73 – 1.65 (m, 3H), 1.60 – 1.51 (m, 1H), 1.35 (p, *J* = 8.5 Hz, 2H).

^{13}C NMR (101 MHz, $CDCl_3$) δ 175.3, 172.4, 168.5, 134.0, 132.2, 123.3, 51.9, 51.9, 41.1, 37.7, 35.9, 31.4, 28.4, 24.3.

HRMS: (ESI$^+$) calculated for $C_{18}H_{21}NNaO_6^+$ [M+Na$^+$]: 370.1261, found 370.1265.

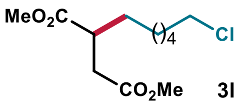

3l

Dimethyl 2-(6-chlorohexyl)succinate (3l): Synthesized according to General Procedure using *fac*-Ir(ppy)$_3$ (400 µL stock solution, 0.3 mol%), HAT **4c** (ethyl thioglycolate, 4.4 µL, 0.04 mmol, 20 mol%), γ-terpinene (32.0 µL, 0.2 mmol, 1 equiv.), dimethyl fumarate **1a** (28.8 mg, 0.2 mmol, 1 equiv.) 6-chloro-1-hexene **2l** (79.5 µL, 0.6 mmol, 3 equiv.). Upon 36 hours stirring, the crude mixture was purified by flash column chromatography on silica gel (20% EtOAc in hexanes as eluent) to afford **3l** (26.5 mg, 50 % yield) as a colorless oil.

^{1}H NMR (400 MHz, $CDCl_3$) δ 3.72 (s, 3H), 3.69 (s, 3H), 3.54 (t, *J* = 6.7 Hz, 2H), 2.86 (dddd, *J* = 9.2, 7.4, 6.2, 5.2 Hz, 1H), 2.73 (dd, *J* = 16.5, 9.2 Hz, 1H), 2.45 (dd, *J* = 16.5, 5.2 Hz, 1H), 1.77 (dq, *J* = 8.8, 6.8 Hz, 2H), 1.70 – 1.61 (m, 1H), 1.58 – 1.49 (m, 1H), 1.48 – 1.40 (m, 2H), 1.38 – 1.26 (m, 4H).
^{13}C NMR (101 MHz, $CDCl_3$) δ 175.51, 172.53, 51.96, 51.90, 45.13, 41.23, 35.97, 32.59, 31.92, 28.75, 26.89, 26.75.

HRMS: (ESI$^+$) calculated for $C_{12}H_{21}ClNaO_4^+$ [M+Na$^+$]: 287.1021, found 287.1029.

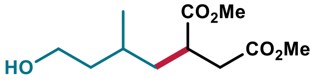

3m

Dimethyl 2-(4-hydroxy-2-methylbutyl)succinate (3m): Synthesized according to General Procedure using *fac*-Ir(ppy)$_3$ (400 µL stock solution, 0.3 mol%), HAT **4c** (Ethyl thioglycolate, 4.4 µL, 0.04 mmol, 20 mol%), γ-terpinene (32.0 µL, 0.2 mmol, 1 equiv.), dimethyl fumarate **1a** (28.8 mg, 0.2 mmol, 1 equiv.) and 3-methyl-3-buten-1-ol **2m** (60.5 µL, 0.6 mmol, 3 equiv.). Upon 14 hours stirring, the crude mixture was purified by flash column chromatography on silica gel (17% to 50% EtOAc in hexanes as eluent) to afford **3m** (30.0 mg, 64% yield, 1:1 dr) as a colorless oil.

^{1}H NMR (500 MHz, $CDCl_3$, mixture of diastereomers) δ 3.68 (s, 6H), 3.72 – 3.59 (m, 4H), 3.65 (s, 3H), 3.65 (s, 3H), 2.96 – 2.87 (m, 2H), 2.69 (dd, *J* = 16.5, 8.7 Hz, 1H), 2.66 (dd, *J* = 16.7, 9.3 Hz,

1H), 2.44 (dd, J = 16.6, 5.0 Hz, 1H), 2.42 (dd, J = 16.5, 5.7 Hz, 1H), 1.71 (ddd, J = 13.9, 9.4, 4.8 Hz, 1H), 1.66 – 1.49 (m, 5H), 1.47 – 1.34 (m, 3H), 1.21 (ddd, J = 13.5, 8.8, 5.4 Hz, 1H), 0.93 (d, J = 6.5 Hz, 3H), 0.90 (d, J = 6.5 Hz, 3H).

^{13}C NMR (126 MHz, $CDCl_3$, mixture of diastereomers) δ 175.8, 175.8, 172.5, 172.4, 60.7, 60.6, 52.0, 52.0, 51.9, 51.9, 39.8, 39.4, 39.4, 39.3, 39.2, 39.2, 36.8, 35.9, 27.6, 27.4, 19.6, 19.6.

HRMS: (ESI^+) calculated for $C_{11}H_{20}NaO_5^+$ [M+Na^+]: 255.1203, found 255.1205.

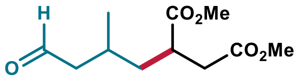

3n

Dimethyl-2-(2-methyl-4-oxobutyl)succinate (3n): Synthesized according to General Procedure using *fac*-Ir(ppy)$_3$ (400 µL stock solution, 0.3 mol%), HAT **4c** (ethyl thioglycolate, 4.4 µL, 0.04 mmol, 20 mol%), γ-terpinene (32.0 µL, 0.2 mmol, 1 equiv.), dimethyl fumarate **1a** (28.8 mg, 0.2 mmol, 1 equiv.) and 3-methylbut-3-enal **2n** as a solution in DCM (50.5 mg, 0.6 mmol, 3 equiv.). Upon 14 hours stirring, the crude mixture was purified by flash column chromatography on silica gel (5% to 20% EtOAc in hexanes as eluent) to afford **3n** (17.1 mg, 37% yield, 1:1 dr) as a colorless oil.

^{1}H NMR (500 MHz, $CDCl_3$, mixture of diastereomers) δ 9.73 (dd, J = 2.3, 1.4 Hz, 1H), 9.71 (dd, J = 2.3, 1.8 Hz, 1H), 3.70 (s, 3H), 3.69 (s, 3H), 3.66 (s, 3H), 3.66 (s, 3H), 2.94 – 2.84 (m, 2H), 2.74 – 2.67 (m, 2H), 2.50 (ddd, J = 16.6, 5.1, 1.5 Hz, 1H), 2.46 (dd, J = 16.7, 5.2 Hz, 1H), 2.42 (dd, J = 16.7, 5.8 Hz, 1H), 2.38 (ddd, J = 16.5, 5.7, 2.2 Hz, 1H), 2.30 – 2.23 (m, 2H), 2.13 – 2.01 (m, 2H), 1.70 (ddd, J = 13.7, 9.5, 5.1 Hz, 1H), 1.62 (dt, J = 13.8, 7.5 Hz, 1H), 1.45 (dt, J = 13.9, 7.0 Hz, 1H), 1.32 (ddd, J = 13.7, 9.2, 5.2 Hz, 1H), 1.00 (d, J = 6.6 Hz, 3H), 0.96 (d, J = 6.6 Hz, 3H).

^{13}C NMR (126 MHz, $CDCl_3$, mixture of diastereomers) δ 202.0, 202.0, 175.4, 175.2, 172.3, 172.2, 52.1, 52.1, 52.0 (2C), 51.1, 50.5, 39.3, 39.1, 39.1, 38.8, 36.7, 35.9, 26.1, 26.0, 20.0, 19.6.

HRMS: (ESI^+) calculated for $C_{11}H_{18}NaO_5^+$ [M+Na^+]: 253.1046, found 255.1044.

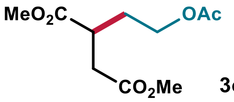

3o

Dimethyl-2-(2-acetoxyethyl)succinate (3o): Synthesized according to General Procedure using *fac*-Ir(ppy)$_3$ (400 µL stock solution, 0.3 mol%), HAT **4c** (ethyl thioglycolate, 4.4 µL, 0.04 mmol, 20 mol%), γ-terpinene (32.0 µL, 0.2 mmol, 1 equiv.), dimethyl fumarate **1a** (28.8 mg, 0.2 mmol, 1 equiv.) and vinyl acetate **2o** (55.0 µL, 0.6 mmol, 3 equiv.). Upon 14 hours stirring, the crude mixture was purified by flash column chromatography on silica gel (10% EtOAc in hexanes as eluent) to afford **3o** (23.5 mg, 51 % yield) as a colorless oil.

^{1}H NMR (400 MHz, $CDCl_3$) δ 4.11 (t, J = 6.3 Hz, 2H), 3.71 (s, 3H), 3.68 (s, 3H), 3.02 – 2.93 (m, 1H), 2.76 (dd, J = 16.6, 8.7 Hz, 1H), 2.51 (dd, J = 16.7, 5.6 Hz, 1H), 2.07 – 1.98(m, 4H), 1.90 – 1.8 (m, 1H).

^{13}C NMR (101 MHz, $CDCl_3$) δ 174.7, 172.1, 171.0, 62.0, 52.2, 52.0, 38.4, 35.7, 30.6, 21.0.

HRMS: (ESI^+) calculated for $C_{10}H_{16}NaO_6^+$ [M+Na^+]: 255.0839, found 255.0838.

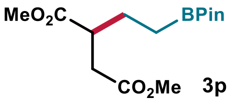

3p

Dimethyl-2-(2-(4,4,5,5-tetramethyl-1,3,2-dioxaborolan-2-yl)ethyl)succinate (3p) : Synthesized according to General Procedure using *fac*-Ir(ppy)$_3$ (400 µL stock solution, 0.3 mol%), HAT **4c** (ethyl thioglycolate, 4.4 µL, 0.04 mmol, 20 mol%), γ-terpinene (32.0 µL, 0.2 mmol, 1 equiv.), dimethyl fumarate **1a** (28.8 mg, 0.2 mmol, 1 equiv.) and vinylboronic acid pinacol ester **2p** (102.0 µL, 0.6 mmol, 3 equiv.). Upon 14 hours stirring, the crude mixture was purified by flash column chromatography on silica gel (20% EtOAc in hexanes as eluent) to afford **3p** (50.5 mg, 84 % yield) as a colorless oil.

^{1}H NMR (500 MHz, CDCl$_3$) δ 3.69 (s, 3H), δ 3.67 (s, 3H), 2.83 (dddd, *J* = 9.6, 7.5, 6.5, 4.7 Hz, 1H), 2.71 (dd, *J* = 16.6, 9.7 Hz, 1H), 2.44 (dd, *J* = 16.6, 4.8 Hz, 1H), 1.79 – 1.69 (m, 1H), 1.69 – 1.62 (m, 1H), 1.24 (s, 12H), 0.77 (d, *J* = 8.0 Hz, 2H).

^{13}C NMR (126 MHz, CDCl$_3$) δ 175.5, 172.6, 83.3, 51.8, 43.1, 35.7, 29.8, 26.6, 24.9.

HRMS: (ESI$^+$) calculated for $C_{14}H_{25}NaO_6B^+$ [M+Na$^+$]: 322.1673, found 322.1664.

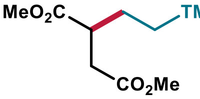

3q

Dimethyl-2-(2-(trimethylsilyl)ethyl)succinate (3q): Synthesized according to General Procedure using *fac*-Ir(ppy)$_3$ (400 µL stock solution, 0.3 mol%), HAT **4c** (ethyl thioglycolate, 4.4 µL, 0.04 mmol, 20 mol%), γ-terpinene (32.0 µL, 0.2 mmol, 1 equiv.), dimethyl fumarate **1a** (28.8 mg, 0.2 mmol, 1 equiv.) and vinyltrimethylsilane **2q** (88.0 µL, 0.6 mmol, 3 equiv.). Upon 14 hours stirring, the crude mixture was purified by flash column chromatography on silica gel (2% EtOAc in hexanes as eluent) to afford **3q** (32.0 mg, 65 % yield) as a colorless oil.

^{1}H NMR (400 MHz, CDCl$_3$) δ 3.70 (s, 3H), 3.67 (s, 3H), 2.87 – 2.78 (m, 1H), 2.71 (dd, *J* = 16.3, 9.3 Hz, 1H), 2.45 (dd, *J* = 16.3, 5.0 Hz, 1H), 1.68 – 1.58 (m, 1H), 1.56 – 1.48 (m, 1H), 0.51 – 0.41 (m, 2H), -0.02 (s, 9H).

^{13}C NMR (126 MHz, CDCl$_3$) δ 175.5, 172.7, 51.9, 51.8, 44.0, 35.4, 26.6, 13.7, -1.7.

HRMS: (ESI$^+$) calculated for $C_{11}H_{22}NaO_4Si^+$ [M+Na$^+$]: 269.1180, found 269.1177.

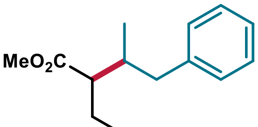

3r

Dimethyl 2-(1-phenylpropan-2-yl)succinate (3r): Synthesized according to General Procedure using *fac*-Ir(ppy)$_3$ (400 µL stock solution, 0.3 mol%), PhSH (4.1 µL, 0.04 mmol, 20 mol%), γ-terpinene (32.0 µL, 0.2 mmol, 1 equiv.), dimethyl fumarate **1a** (28.8 mg, 0.2 mmol, 1.0 equiv.) and *trans*-β-methylstyrene **23** (78.0 µL, 0.6 mmol, 3 equiv.). Upon 14 hours stirring, the crude mixture was purified by flash column chromatography on silica gel (3% to 9% EtOAc in hexanes as eluent) to afford **3r** (23.2 mg, 44% yield, 1:1 dr) as a colorless oil.

^{1}H NMR (500 MHz, CDCl$_3$) δ 7.31 – 7.25 (m, 4H), 7.22 – 7.17 (m, 2H), 7.17 – 7.13 (m, 4H), 3.72 (s, 3H), 3.70 (s, 3H), 3.68 (s, 3H), 3.66 (s, 3H), 2.96 – 2.74 (m, 5H), 2.69 (dd, *J* = 13.5, 5.8 Hz, 1H), 2.50 – 2.35 (m, 4H), 2.29 – 2.20 (m, 1H), 2.14 – 2.05 (m, 1H), 0.88 (d, *J* = 6.8 Hz, 3H), 0.84 (d, *J* = 6.8 Hz, 3H).

^{13}C NMR (126 MHz, $CDCl_3$, mixture of diastereomers) δ 175.0, 174.4, 173.0, 172.8, 140.3, 140.1, 129.3, 129.1, 128.5, 128.4, 126.3, 126.2, 52.0, 52.0, 51.9, 51.8, 45.7, 45.6, 40.9, 40.7, 37.5, 36.8, 33.7, 31.9, 16.6, 16.1.

HRMS: (ESI^+) calculated for $C_{15}H_{20}NaO_4^+$ [$M+Na^+$]: 287.1254, found 287.1261.

Dimethyl 2-(2-methyl-4-((5-((3aS,4S,6aR)-2-oxohexahydro-1H-thieno[3,4-d]imidazol-4-yl)pentanoyl)oxy)butyl)succinate (3s): Synthesized according to General Procedure using *fac*-

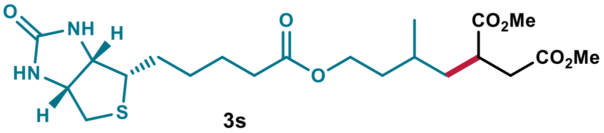

Ir(ppy)$_3$ (400 µL stock solution, 0.3 mol%), HAT **4c** (ethyl thioglycolate, 4.4 µL, 0.04 mmol, 20 mol%), γ-terpinene (32.0 µL, 0.2 mmol, 1 equiv.), dimethyl fumarate **1a** (28.8 mg, 0.2 mmol, 1 equiv.) and 3-methylbut-3-en-1-yl 5-((3aS,4S,6aR)-2-oxohexahydro-1H-thieno[3,4-d]imidazol-4-yl)pentanoate **2s** (187.4 mg, 0.6 mmol, 3 equiv.). Upon 14 hours stirring, ^{1}H NMR analysis (using CH_2Br_2 as internal standard) revealed 62% yield of **3s**. The crude mixture was purified by flash column chromatography on silica gel (3% MeOH in DCM as eluent) to afford a mixture of **3s** (54.2 mg, 59% yield, 1:1:1:1 dr) and **2s** (148.8 mg, 0.47 mmol) as colorless turbid oil. To obtain an analytically pure sample, preparative HPLC has been performed (column: XBridge C18 150x4.6 mm, 5µm; mobile phase: H2O:MeOH 55.45; flow: 1ml/min; wavelength: 200 nm).

^{1}H NMR (500 MHz, $CDCl_3$, mixture of diastereomers) δ 5.23 (br. s, 2H), 4.88 (br. s, 2H), 4.54 – 4.49 (m, 2H), 4.35 – 4.30 (m, 2H), 4.15 – 4.04 (m, 4H), 3.70 (s, 3H), 3.70 (s, 3H), 3.68 (s, 6H), 3.21 – 3.13 (m, 2H), 2.96 – 2.87 (m, 4H), 2.76 – 2.63 (m, 4H), 2.43 (dd, J = 16.6, 7.5 Hz, 1H), 2.42 (dd, J = 16.6, 8.1 Hz, 1H), 2.35 – 2.29 (m, 4H), 1.77 – 1.39 (m, 20H), 1.30 – 1.22 (m, 2H), 0.96 (d, J = 6.5 Hz, 3H), 0.92 (d, J = 6.3 Hz, 3H).

^{13}C NMR (126 MHz, $CDCl_3$, mixture of diastereomers, peak overlapping observed) δ 175.7, 175.6, 175.6, 173.7, 172.5, 172.4, 163.1, 62.6, 62.6 (2C), 62.5, 62.0 (2C), 62.0, 62.0, 60.2, 55.4, 55.4, 52.1, 52.0, 52.0, 52.0, 40.7, 39.5, 39.5, 39.3, 39.2, 39.2, 36.8, 36.1, 35.7, 35.7, 35.2, 34.0, 34.0, 28.5, 28.5, 28.5, 28.4, 28.4 (2C), 28.4, 28.4, 28.1, 28.0, 27.9, 27.9, 24.9, 24.9, 19.5, 19.5, 19.3.

HRMS: (ESI^+) calculated for $C_{21}H_{34}N_2NaO_7S^+$ [$M+Na^+$]: 481.1979, found 481.1979.

Dimethyl 2-(((1S,2S,4aR,4bR,7S,8R,9aS,10S,10aR)-2,7-dihydroxy-10-(methoxycarbonyl)-1-methyl-13-oxo-1,2,4b,5,6,7,8,9,10,10a-decahydro-4a,1-(epoxymethano)-7,9a-methanobenzo[a]azulen-8-yl)methyl)succinate (3t): Synthesized according to General Procedure using *fac*-Ir(ppy)$_3$ (400 µL stock solution, 0.3 mol%), HAT **4c** (Ethyl thioglycolate, 4.4 µL, 0.04 mmol, 20 mol%), γ-terpinene (32.0 µL, 0.2 mmol, 1 equiv.), dimethyl fumarate **1a** (28.8 mg, 0.2 mmol, 1 equiv.) and *Gibberellic acid* methyl ester **2t** (216.0 mg, 0.6 mmol, 3 equiv.) in DCE (4 ml) and acetone (1 ml). Upon

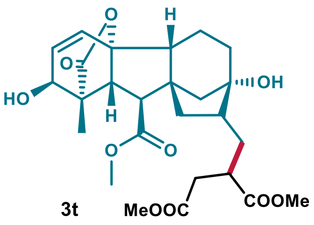

14 hours stirring, the crude mixture was purified by flash column chromatography on silica gel (67 % EtOAc in hexanes as eluent) to afford **3t** (58.5 mg, 58% yield, 1:1 dr) as a light-yellow oil.

^{1}H NMR (400 MHz, $CDCl_3$, mixture of diastereomers) δ 6.29 (d, *J* = 9.2 Hz, 1H), 5.89 (dd, *J* = 9.3, 3.7 Hz, 1H), 4.11 (d, *J* = 3.6 Hz, 1H), 3.73 (d, *J* = 3.1 Hz, 3H), 3.68 (s, 3H), 3.66 (s, 3H), 3.14 (dd, *J* = 10.6, 3.8 Hz, 1H), 2.85 – 2.82 (m, 1H), 2.75 – 2.64 (m, 2H), 2.48 (ddd, *J* = 21.7, 16.7, 5.4 Hz, 1H), 2.16 (s, 1H), 2.14 – 1.22 (m, 12H), 1.20 (d, *J* = 1.5 Hz, 3H).

^{13}C NMR (101 MHz, $CDCl_3$, mixture of diastereomers, peak overlapping observed) δ 178.7, 178.7, 175.6, 175.3, 173.0, 173.0, 172.4, 172.3, 132.8, 132.7, 132.7, 132.7, 90.7, 90.7, 78.5, 78.4, 69.9, 69.9, 53.8, 53.7, 53.6, 52.8, 52.8, 52.4, 52.3, 52.2, 52.1, 52.0, 52.0, 52.0, 51.9, 51.9, 46.3, 45.0, 44.6, 42.3, 42.2, 40.4, 40.1, 36.3, 36.1, 33.7, 33.4, 31.0, 30.3, 30.3, 16.6, 16.6, 14.5.

HRMS: (ESI^+) calculated for $C_{26}H_{34}NaO_{10}^+$ [$M+Na^+$]: 529.2044, found 529.2050.

Dimethyl 2-(4-((2-(4-(2-(4-chlorobenzamido)ethyl)phenoxy)-2-methylpropanoyl)oxy)-2-methylbutyl)succinate (3u): Synthesized according to General Procedure using *fac*-Ir(ppy)$_3$ (400 μL stock solution, 0.3 mol%), HAT **4c** (Ethyl thioglycolate, 4.4 μL, 0.04 mmol, 20 mol%), γ-terpinene (32.0 μL, 0.2 mmol, 1 equiv.), dimethyl fumarate **1a** (28.8 mg, 0.2 mmol, 1 equiv.) and 3-methylbut-3-en-1-yl 2-(4-(2-(4-chlorobenzamido)ethyl)phenoxy)-2-methylpropanoate **2u** (257.9 mg, 0.6 mmol, 3 equiv.). Upon 14 hours stirring, the crude mixture was purified by flash column chromatography on silica gel (15% to 30% EtOAc in hexanes as eluent) to afford **3u** (41.6 mg, 37% yield, 1:1 dr) as a colorless oil and **2u** (196.7 mg, 0.46 mmol) as a white solid.

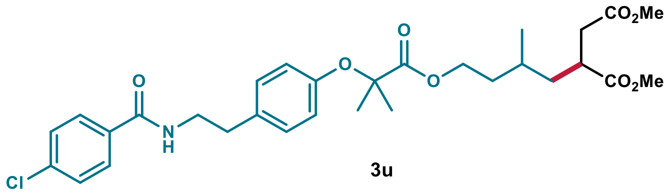

3u

^{1}H NMR (500 MHz, $CDCl_3$, mixture of diastereomers) δ 7.65 – 7.60 (m, 4H), 7.38 – 7.34 (m, 4H), 7.09 – 7.06 (m, 4H), 6.79 – 6.75 (m, 4H), 6.28 (t, *J* = 5.2 Hz, 2H), 4.22 – 4.15 (m, 4H), 3.67 – 3.62 (m, 16H), 2.91 – 2.79 (m, 6H), 2.65 (dd, *J* = 16.5, 9.1 Hz, 1H), 2.57 (dd, *J* = 16.6, 9.5 Hz, 1H), 2.35 (dd, *J* = 16.5, 5.5 Hz, 1H), 2.33 (dd, *J* = 16.7, 4.9 Hz, 1H), 1.77 – 1.32 (m, 9H), 1.57 (s, 6H), 1.56 (s, 3H), 1.56 (s, 3H), 1.21 (ddd, *J* = 14.3, 9.0, 5.4 Hz, 1H), 0.90 (d, *J* = 6.4 Hz, 3H), 0.84 (d, *J* = 6.2 Hz, 3H). ^{13}C NMR (126 MHz, $CDCl_3$, mixture of diastereomers, peak overlapping observed) δ 175.6, 175.4, 174.4, 174.4, 172.4, 172.3, 166.5, 166.5, 154.3, 154.2, 137.7, 133.2, 133.2, 132.6, 132.5, 129.6, 128.9, 128.4, 119.5, 119.3, 79.2, 79.2, 63.5, 63.4, 52.0, 52.0, 51.9, 51.9, 41.4, 41.4, 39.4, 39.2, 39.1, 39.1, 36.7, 35.8, 35.6, 35.0, 34.8, 34.8, 27.9, 27.7, 25.6, 25.6, 25.3, 25.3, 19.2, 19.0.

HRMS: (ESI^+) calculated for $C_{30}H_{38}ClNNaO_8^+$ [$M+Na^+$]: 598.2178, found 598.2184.

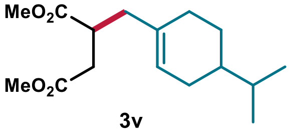

3v

Dimethyl 2-((4-isopropylcyclohex-1-en-1-yl)methyl)succinate (3v): Synthesized according to General Procedure using *fac*-Ir(ppy)$_3$ (400 μL stock solution, 0.3 mol%), HAT **4c** (ethyl thioglycolate, 4.4 μL, 0.04 mmol, 20 mol%), γ-terpinene (32.0 μL, 0.2 mmol, 1 equiv.), dimethyl

fumarate **1a** (28.8 mg, 0.2 mmol, 1 equiv.) and (-)-β-pinene **2v** (93.5 µL, 0.6 mmol, 3 equiv.). Upon 14 hours stirring, the crude mixture was purified by flash column chromatography on silica gel (6% EtOAc in hexanes as eluent) to afford **3v** (42.0 mg, 74% yield, 1:1 dr) as a colorless oil.

^{1}H NMR (500 MHz, $CDCl_3$, mixture of diastereomers) δ 5.43 – 5.39 (m, 2H), 3.67 (s, 3H), 3.66 (s, 3H), 3.65 (s, 6H), 3.02 – 2.95 (m, 2H), 2.64 (dd, *J* = 16.9, 9.5 Hz, 1H), 2.61 (dd, *J* = 16.9, 9.8 Hz, 1H), 2.41 (dd, *J* = 16.9, 4.8 Hz, 1H), 2.40 (dd, *J* = 16.9, 5.0 Hz, 1H), 2.36 – 2.28 (m, 2H), 2.11 – 2.03 (m, 2H), 2.03 – 1.88 (m, 6H), 1.79 – 1.64 (m, 4H), 1.48 – 1.39 (m, 2H), 1.23 – 1.12 (m, 4H), 0.88 – 0.84 (m, 12H).

^{13}C NMR (126 MHz, $CDCl_3$, mixture of diastereomers) δ 175.6, 175.5, 172.8, 172.7, 134.0, 133.9, 124.8, 124.7, 51.9, 51.9, 51.8, 51.8, 40.5, 40.2, 40.1, 40.0, 39.8, 39.6, 35.4, 35.1, 32.3, 32.3, 29.1, 29.1, 28.6, 28.5, 26.5, 26.4, 20.1, 20.0, 19.8, 19.8.

HRMS: (ESI^+) calculated for $C_{16}H_{26}NaO_4^+$ [$M+Na^+$]: 305.1723, found 305.1728.

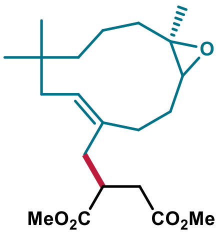

3w

Dimethyl 2-(((11R,Z)-7,7,11-trimethyl-12-oxabicyclo[9.1.0]dodec-4-en-4-yl)methyl)succinate (3w): Synthesized according to General Procedure using *fac*-Ir(ppy)$_3$ (400 µL stock solution, 0.3 mol%), HAT **4c** (ethyl thioglycolate, 4.4 µL, 0.04 mmol, 20 mol%), γ-terpinene (32.0 µL, 0.2 mmol, 1 equiv.), dimethyl fumarate **1a** (28.8 mg, 0.2 mmol, 1 equiv.) and (−)-*Caryophyllene oxide* **2w** (132.0 mg, 0.6 mmol, 3 equiv.). Upon 14 hours stirring, the crude mixture was purified by flash column chromatography on silica gel (6% to 20% EtOAc in hexanes as eluent) to afford **3w** (48.5 mg, 67% yield, 1:1 dr) as a colorless oil and (−)-Caryophyllene oxide **2w** (88.5 mg, 0.4 mmol) as white solid.

^{1}H NMR (500 MHz, $CDCl_3$, mixture of diastereomers) δ 5.35 (dd, *J* = 11.3, 2.4 Hz, 1H), 5.30 (dd, *J* = 11.3, 2.5 Hz, 1H), 3.70 (s, 3H), 3.65 (s, 3H), 3.64 (s, 3H), 3.64 (s, 3H), 2.93 (dddd, *J* = 8.9, 8.2, 6.6, 5.7 Hz, 1H), 2.89 – 2.83 (m, 1H), 2.74 (dd, *J* = 6.8, 1.8 Hz, 1H), 2.72 (dd, *J* = 6.8, 2.1 Hz, 1H), 2.70 (dd, *J* = 16.8, 8.9 Hz, 1H), 2.59 (dd, *J* = 16.8, 9.5 Hz, 1H), 2.56 (dd, *J* = 13.8, 8.5 Hz, 1H), 2.43 (dd, *J* = 16.7, 5.7 Hz, 1H), 2.43 (dd, *J* = 16.9, 4.6 Hz, 1H), 2.37 – 2.27 (m, 3H), 2.23– 2.14 (m, 3H), 2.13 – 2.07 (m, 2H), 2.05 – 1.91 (m, 5H), 1.67 – 1.57 (m, 2H), 1.51 – 1.34 (m, 4H), 1.23 – 1.12 (m, 4H), 1.15 (s, 6H), 0.92 (d, *J* = 0.6 Hz, 3H), 0.91 (d, *J* = 0.6 Hz, 3H), 0.84 (s, 3H), 0.83 (s, 3H), 0.80 – 0.73 (m, 4H).

^{13}C NMR (126 MHz, $CDCl_3$, mixture of diastereomers, peak overlapping observed) δ 175.3, 174.8, 172.5, 172.3, 134.1, 134.0, 127.9, 127.4, 62.8, 62.8, 62.7, 62.7, 52.2, 52.0, 51.9, 51.8, 40.8, 40.8, 40.3 (2C), 38.5, 38.5, 37.4, 37.2, 35.8, 35.5, 34.8, 34.7, 33.2, 33.2, 31.0, 30.6, 29.6, 29.6, 28.3 (2C), 24.6, 24.5, 18.5, 18.4, 18.0 (2C).

HRMS: (ESI^+) calculated for $C_{21}H_{34}NaO_5^+$ [$M+Na^+$]: 389.2298, found 389.2308.

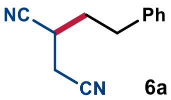

6a

2-phenethylsuccinonitrile (6a): Synthesized according to the General Procedure using *fac*-Ir(ppy)$_3$ (800 µL stock solution, 0.6 mol%), PhSH (4.1µL, 0.04 mmol, 20 mol%), γ-terpinene (64.1µL, 0.4 mmol, 2 equiv.), fumaronitrile (23.5 mg, 0.3 mmol, 1.5 equiv.) and styrene **2a** (23.0 µL, 0.2 mmol, 1 equiv.). Upon 14 hours stirring, the crude mixture was purified by flash column chromatography on silica gel (10% EtOAc in hexanes as eluent) to afford **6a** (29.0 mg, 79% yield) as a light-yellow oil.

^{1}H NMR (500 MHz, CDCl$_3$) δ 7.36 – 7.31 (m, 2H), 7.28 – 7.24 (m, 1H), 7.24 – 7.19 (m, 2H), 2.97 (ddd, J = 13.7, 8.2, 5.2 Hz, 1H), 2.87 – 2.76 (m, 2H), 2.75 – 2.65 (m, 2H), 2.20 – 2.10 (m, 1H), 2.06 (dtd, J = 13.5, 8.2, 5.2 Hz, 1H).

^{13}C NMR (126 MHz, CDCl$_3$) δ 138.8, 129.0, 128.5, 127.0, 118.7, 115.4, 33.2, 32.8, 27.7, 21.2.

Matching reported literature data.[8]

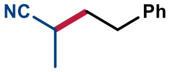

6b

2-methyl-4-phenylbutanenitrile (6b): Synthesized according to the General Procedure using 3DPA2FBN (6.4 mg, 5.0 mol%), PhSH (4.1 µL, 0.04 mmol, 20 mol%), 2,4,6-collidine (26.5µL, 0.2 mmol, 1 equiv.), γ-terpinene (96.0 µL, 0.6 mmol, 3 equiv.), acrylonitrile (13.0µL, 0.2 mmol, 1 equiv.) and styrene **2a** (68.5 µL, 0.6 mmol, 3 equiv.). Upon 14 hours stirring, the crude mixture was purified by flash column chromatography on silica gel (2% EtOAc in hexanes as eluent) to afford **6b** (20.5 mg, 64% yield) as a colorless oil.

^{1}H NMR (500 MHz, CDCl$_3$) δ 7.33 – 7.28 (m, 2H), 7.24 – 7.18 (m, 3H), 2.88 (ddd, J = 14.3, 9.1, 5.4 Hz, 1H), 2.80 – 2.71 (m, 1H), 2.64 – 2.53 (m, 1H), 1.99 – 1.92 (m, 1H), 1.84 (dddd, J = 13.6, 9.1, 7.5, 5.5 Hz, 1H), 1.34 (d, J = 7.1 Hz, 3H).

^{13}C NMR (126 MHz, CDCl$_3$) δ 140.2, 128.7, 128.5, 126.5, 122.1, 35.8, 33.3, 24.9, 18.1.

Matching reported literature data.[9]

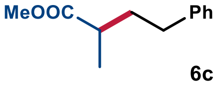

6c

methyl-2-methyl-4-phenylbutanoate (6c): Synthesized according to the General Procedure using 3DPA2FBN (6.4 mg, 5.0 mol%), PhSH (4.1 µL, 0.04 mmol, 20 mol%), 2,4,6-collidine (26.5µL, 0.2 mmol, 1 equiv.), γ-terpinene (96.0 µL, 0.6 mmol, 3 equiv.), methyl acrylate (18.0µL, 0.2 mmol, 1 equiv.) and styrene **2a** (68.5 µL, 0.6 mmol, 3 equiv.). Upon 14 hours stirring, the crude mixture was purified by flash column chromatography on silica gel (2% EtOAc in hexanes as eluent) to afford **6c** (26.0 mg, 68% yield) as a colorless oil.

^{1}H NMR (400 MHz, CDCl$_3$) δ 7.31 – 7.27 (m, 2H), 7.22 – 7.16 (m, 3H), 3.68 (s, 3H), 2.62 (t, J = 8.0 Hz, 2H), 2.54 – 2.44 (m, 1H), 2.09 – 1.96 (m, 1H), 1.79 – 1.68 (m, 1H), 1.20 (d, J = 7.0 Hz, 3H).

^{13}C NMR (101 MHz, CDCl$_3$) δ 177.1, 141.7, 128.5, 128.5, 126.0, 51.6, 39.0, 35.5, 33.6, 17.2.

Matching reported literature data.[3]

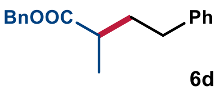

Benzyl-2-methyl-4-phenylbutanoate (6d): Synthesized according to the General Procedure using 3DPA2FBN (6.4 mg, 5.0 mol%), PhSH (4.1 µL, 0.04 mmol, 20 mol%), 2,4,6-collidine (26.5µL, 0.2 mmol, 1 equiv.), γ-terpinene (96.0 µL, 0.6 mmol, 3 equiv.), benzyl acrylate (30.5µL, 0.2 mmol, 1 equiv.) and styrene **2a** (68.5 µL, 0.6 mmol, 3 equiv.). Upon 14 hours stirring, the crude mixture was purified by flash column chromatography on silica gel (2% EtOAc in hexanes as eluent) to afford **6d** (39.5 mg, 73% yield) as a colorless oil.

^{1}H NMR (500 MHz, $CDCl_3$) δ 7.38 – 7.35 (m, 4H), 7.35 – 7.31 (m, 1H), 7.28 – 7.25 (m, 2H), 7.20 – 7.16 (m, 1H), 7.15 – 7.11 (m, 2H), 5.13 (s, 2H), 2.60 (t, *J* = 8.0 Hz, 2H), 2.56 – 2.52 (m, 1H), 2.08 – 1.99 (m, 1H), 1.78 – 1.71 (m, 1H), 1.22 (d, *J* = 7.0 Hz, 3H).

^{13}C NMR (126 MHz, $CDCl_3$) δ 176.4, 141.7, 136.3, 128.7, 128.5, 128.5, 128.3, 128.2, 126.0, 66.2, 39.2, 35.5, 33.5, 17.2.

Matching reported literature data.[10]

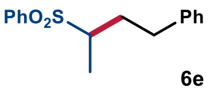

((4-Phenylbutan-2-yl)sulfonyl)benzene (6e): Synthesized according to the General Procedure using 3DPA2FBN (6.4 mg, 5.0 mol%), PhSH (4.1 µL, 0.04 mmol, 20 mol%), 2,4,6-collidine (26.5µL, 0.2 mmol, 1 equiv.), γ-terpinene (96.0 µL, 0.6 mmol, 3 equiv.), phenyl vinyl sulfone (33.5 mg, 0.2 mmol, 1 equiv.) and styrene **2a** (68.5 µL, 0.6 mmol, 3 equiv.). Upon 14 hours stirring, the crude mixture was purified by flash column chromatography on silica gel (5% EtOAc in hexanes as eluent) to afford **6e** (26.0 mg, 47% yield) as a white solid.

^{1}H NMR (400 MHz, $CDCl_3$) δ 7.90 – 7.84 (m, 2H), 7.69 – 7.63 (m, 1H), 7.59 – 7.53 (m, 2H), 7.32 – 7.26 (m, 2H), 7.25 – 7.19 (m, 1H), 7.15 – 7.10 (m, 2H), 3.05 (dtd, *J* = 13.7, 6.8, 3.8 Hz, 1H), 2.84 (ddd, *J* = 14.3, 9.4, 5.3 Hz, 1H), 2.62 (ddd, *J* = 13.9, 9.0, 7.4 Hz, 1H), 2.34 (dddd, *J* = 13.4, 9.4, 7.4, 3.8 Hz, 1H), 1.82 – 1.68 (m, 1H), 1.34 (d, *J* = 6.9 Hz, 3H).

^{13}C NMR (101 MHz, $CDCl_3$) δ 140.1, 137.2, 133.6, 129.1, 128.9, 128.5, 128.3, 126.3, 59.1, 32.4, 30.7, 13.2. Matching reported literature data.[11]

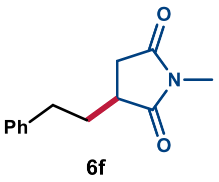

1-Methyl-3-phenethylpyrrolidine-2,5-dione (6f): Synthesized according to General Procedure using *fac*-Ir(ppy)$_3$ (800 µL stock solution, 0.6 mol%), HAT **4b** (3,5-bis(trifluoromethyl)benzenethiol, 6.7 µL, 0.04 mmol, 20 mol%), methyl 4,5-dimethylcyclohexa-1,4-diene-1-carboxylate as H donor (64.0 µL, 0.4 mmol, 2 equiv.), *N*-methylmaleimide (22.0 mg, 0.2 mmol, 1 equiv.) and styrene **2a** (68.5 µL, 0.6 mmol, 3 equiv.). Upon 14 hours stirring, the crude mixture was purified by flash column chromatography on silica gel (20% EtOAc in hexanes as eluent) to afford **6f** (25.0 mg, 58% yield) as a white solid.

^{1}H NMR (300 MHz, $CDCl_3$) δ 7.32 – 7.26 (m, 2H), 7.24 – 7.15 (m, 3H), 2.97 (s, 3H), 2.88 – 2.65 (m, 4H), 2.44 – 2.35 (m, 1H), 2.27 (dtt, J = 11.8, 7.0, 2.3 Hz, 1H), 1.82 (dtd, J = 13.6, 8.7, 6.3 Hz, 1H).

^{13}C NMR (101 MHz, $CDCl_3$) δ 180.0, 176.7, 140.4, 128.7, 128.6, 126.5, 39.3, 34.6, 33.2, 33.1, 24.9. Matching reported literature data.[12]

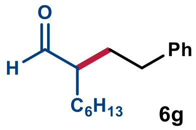

6g

2-Phenethyloctanal (6g): Synthesized according to the General Procedure using 3DPA2FBN (6.4 mg, 5.0 mol%), HAT **4b** (3,5-bis(trifluoromethyl)benzenethiol, 6.7 µL, 0.04 mmol, 20 mol%), methyl 4,5-dimethylcyclohexa-1,4-diene-1-carboxylate as H donor (96.0 µL, 0.6 mmol, 3 equiv.), trans-2-Octenal (89.5 µL, 0.6 mmol, 3 equiv.) and styrene **2a** (23.0 µL, 0.2 mmol, 1 equiv.). Upon 14 hours stirring, the crude mixture was purified by flash column chromatography on silica gel (25% DCM in hexanes as eluent) to afford **6g** (26.5 mg, 57% yield) as a colorless oil.

^{1}H NMR (500 MHz, $CDCl_3$) δ 9.60 (d, J = 2.9 Hz, 1H), 7.31 – 7.27 (m, 2H), 7.22 – 7.15 (m, 3H), 2.68 – 2.55 (m, 2H), 2.29 (ttd, J = 8.1, 5.4, 2.8 Hz, 1H), 2.01 – 1.93 (m, 1H), 1.79 – 1.71 (m, 1H), 1.69 – 1.63 (m, 1H), 1.53 – 1.45 (m, 1H), 1.31 – 1.25 (m, 9H), 0.89 – 0.86 (m, 3H).

^{13}C NMR (126 MHz, $CDCl_3$) δ 205.3, 141.6, 128.6, 128.5, 126.2, 51.4, 33.4, 31.7, 30.6, 29.4, 29.0, 27.0, 22.7, 14.1. Matching reported literature data.[13]

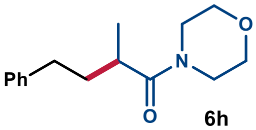

6h

2-Methyl-1-morpholino-4-phenylbutan-1-one (6h): Synthesized according to the General Procedure using *fac*-Ir(ppy)$_3$ (2.0 ml stock solution, 1.5 mol%), HAT **4b** (3,5-bis(trifluoromethyl)benzenethiol, 6.7 µL, 0.04 mmol, 20 mol%), 2,4,6-collidine (26.5 µL, 0.2 mmol, 1 equiv.), methyl 4,5-dimethylcyclohexa-1,4-diene-1-carboxylate as H donor (96.0 µL, 0.6 mmol, 3 equiv.), 4-Acryloylmorpholine (25.0 µL, 0.2 mmol, 1 equiv.) and styrene **2a** (68.5 µL, 0.6 mmol, 3 equiv.). Upon 14 hours stirring, the crude mixture was purified by flash column chromatography on silica gel (25% EtOAc in hexanes as eluent) to afford **6h** (26.5 mg, 58% yield) as a colorless oil.

^{1}H NMR (500 MHz, $CDCl_3$) δ 7.30 – 7.26 (m, 2H), 7.20 – 7.14 (m, 3H), 3.69 – 3.61 (m, 4H), 3.58 (t, J = 4.8 Hz, 2H), 3.37 – 3.28 (m, 2H), 2.69 – 2.56 (m, 3H), 2.09 – 2.01 (m, 1H), 1.74 – 1.67 (m, 1H), 1.14 (d, J = 6.8 Hz, 3H).

^{13}C NMR (126 MHz, $CDCl_3$) δ 174.9, 141.8, 128.6, 128.5, 126.1, 67.2, 66.8, 45.9, 42.1, 35.5, 34.1, 33.5, 17.6. Matching reported literature data.[14]

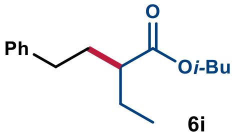

6i

Isobutyl-2-ethyl-4-phenylbutanoate (6i): Synthesized according to the General Procedure using 3DPA2FBN (6.4 mg, 5.0 mol%), PhSH (4.1 µL, 0.04 mmol, 20 mol%), Cs_2CO_3 (32.5 mg, 0.2 mmol, 1 equiv.), γ-terpinene (96.0 µL, 0.6 mmol, 3 equiv), isobutyl trans-2-butenoate (32.0 µL, 0.2 mmol, 1 equiv.), styrene **2a** (68.5 µL, 0.6 mmol, 3 equiv.) and acetonitrile (4 ml) as solvent. Upon 14 hours stirring,

the crude mixture was purified by flash column chromatography on silica gel (1% EtOAc in hexanes as eluent) to afford **6i** (26.5 mg, 53% yield) as a light-yellow oil.

^{1}H NMR (400 MHz, $CDCl_3$) δ 7.31 – 7.26 (m, 2H), 7.20 – 7.16 (m, 3H), 3.89 (dd, *J* = 6.6, 0.9 Hz, 2H), 2.68 – 2.51 (m, 2H), 2.34 (ddd, *J* = 10.5, 9.0, 5.2 Hz, 1H), 2.05 – 1.89 (m, 2H), 1.77 (ddd, *J* = 6.7, 5.0, 2.5 Hz, 1H), 1.68 – 1.55 (m, 2H), 0.96 (d, *J* = 6.7 Hz, 6H), 0.91 (d, *J* = 7.4 Hz, 3H).

^{13}C NMR (101 MHz, $CDCl_3$) δ 176.2, 141.9, 128.5, 128.5, 126.0, 70.5, 47.1, 33.9, 33.9, 27.9, 25.6, 19.3, 11.8. HRMS: (ESI$^+$) calculated for $C_{16}H_{24}NaO_2^+$ [M+Na$^+$]: 271.1669, found 271.1667.

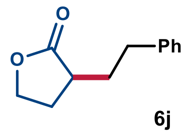

6j

3-Phenethyldihydrofuran-2(3H)-one (6j): Synthesized according to the General Procedure using 3DPA2FBN (6.4 mg, 5.0 mol%), PhSH (4.1 µL, 0.04 mmol, 20 mol%), Cs_2CO_3 (32.5 mg, 0.2 mmol, 1 equiv.), γ-terpinene (96.0 µL, 0.6 mmol, 3 equiv), 2-furanone (14.5 µL, 0.2 mmol, 1 equiv.), styrene **2a** (68.5 µL, 0.6 mmol, 3 equiv.) and acetonitrile (4 mL) as solvent. Upon 14 hours stirring, the crude mixture was purified by flash column chromatography on silica gel (1% EtOAc in hexanes as eluent) to afford **6j** (17.0 mg, 45% yield) as a light-yellow oil.

^{1}H NMR (500 MHz, $CDCl_3$) δ 7.32 – 7.28 (m, 2H), 7.24 – 7.19 (m, 3H), 4.35 (td, *J* = 8.8, 2.8 Hz, 1H), 4.17 (ddd, *J* = 9.8, 9.1, 6.7 Hz, 1H), 2.81 (ddd, *J* = 14.7, 9.1, 6.0 Hz, 1H), 2.72 (ddd, *J* = 13.8, 8.8, 7.1 Hz, 1H), 2.49 (ddt, *J* = 10.2, 8.9, 4.4 Hz, 1H), 2.38 (dddd, *J* = 12.5, 8.7, 6.7, 2.8 Hz, 1H), 2.28 – 2.20 (m, 1H), 1.96 (dtd, *J* = 12.5, 10.0, 8.5 Hz, 1H), 1.77 (dtd, *J* = 13.9, 8.9, 5.9 Hz, 1H).

^{13}C NMR (126 MHz, $CDCl_3$) δ 179.4, 140.8, 128.6, 128.5, 126.3, 66.5, 38.5, 33.4, 32.1, 28.9.

Matching reported literature data.[15]

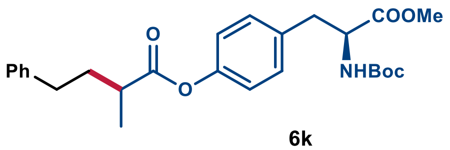

6k

4-((S)-2-((tert-butoxycarbonyl)amino)-3-methoxy-3-oxopropyl)phenyl (S)-2-methyl-4-phenylbutanoate (6k): Synthesized according to the General Procedure using 3DPA2FBN (6.4 mg, 5.0 mol%), PhSH (4.1 µL, 0.04 mmol, 20 mol%), 2,4,6-collidine (26.5µL, 0.2 mmol, 1 equiv.), γ-terpinene (96.0 µL, 0.6 mmol, 3 equiv.), (*S*)-4-(2-((*tert*-butoxycarbonyl)amino)-3-methoxy-3-oxopropyl)phenyl acrylate **1k** (30.5mg, 0.2 mmol, 1 equiv.) and styrene **2a** (68.5 µL, 0.6 mmol, 3 equiv.). Upon 14 hours stirring, the crude mixture was purified by flash column chromatography on silica gel (5% to 10% EtOAc in hexanes as eluent) to afford **6k** (55.5 mg, 61% yield, 1:1 dr) as a colorless oil.

^{1}H NMR (500 MHz, $CDCl_3$) δ 7.33 – 7.28 (m, 2H), 7.24 – 7.19 (m, 3H), 7.17 – 7.12 (m, 2H), 7.02 – 6.99 (m, 2H), 4.99 (d, *J* = 8.3 Hz, 1H), 4.62 – 4.54 (m, 1H), 3.71 (s, 3H), 3.15 – 3.02 (m, 2H), 2.77 – 2.67 (m, 3H), 2.19 – 2.11 (m, 1H), 1.89 – 1.82 (m, 1H), 1.43 (s, 9H), 1.34 (d, *J* = 7.0 Hz, 3H).

^{13}C NMR (126 MHz, $CDCl_3$) δ 175.1, 172.3, 155.2, 150.0, 141.6, 133.7, 130.4, 128.6, 128.6, 126.2, 121.7, 80.2, 54.5, 52.4, 39.3, 37.8, 35.5, 33.6, 28.4, 17.2.

HRMS: (ESI$^+$) calculated for $C_{26}H_{33}NNaO_6{}^+$ [M+Na$^+$]: 478.2200, found 478.2214.

(8R,9S,13S,14S)-13-methyl-17-oxo-7,8,9,11,12,13,14,15,16,17-decahydro-6H-cyclopenta[a]phenanthren-3-yl 2-methyl-4-phenylbutanoate (6l): Synthesized according to the General Procedure using 3DPA2FBN (6.4 mg, 5.0 mol%), PhSH (4.1 µL, 0.04 mmol, 20 mol%), 2,4,6-collidine (26.5µL, 0.2 mmol, 1 equiv.), γ-terpinene (96.0 µL, 0.6 mmol, 3 equiv.), (8R,9S,13S,14S)-13-methyl-17-oxo-7,8,9,11,12,13,14,15,16,17-decahydro-6H-cyclopenta[a]phenanthren-3-yl acrylate **3l** (65.0 mg, 0.2 mmol, 1 equiv.) and styrene **2a** (68.5 µL, 0.6 mmol, 3 equiv.). Upon 14 hours stirring, ^{1}H NMR analysis (CH_2Br_2 as internal standard) revealed 70% yield of **1l**. The crude mixture was purified by flash column chromatography on silica gel (9% EtOAc in hexanes as eluent) to afford **6l** (54.5 mg, 90% purity, 57% adjusted yield, 1:1 dr) as a colorless oil (the impurity was the reduced acrylate, purity calculated from ^{1}H NMR data). To obtain an analytically pure sample, preparative HPLC has been performed (column: ChiralPak IC 250x4.6 mm, 5µm; mobile phase: Hex/EtOH 80:20; flow: 1ml/min; wavelength: 254 nm).

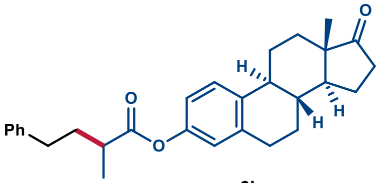
6l

^{1}H NMR (500 MHz, $CDCl_3$) δ 7.32 – 7.27 (m, 3H), 7.24 – 7.19 (m, 3H), 6.85 (dd, *J* = 8.4, 2.6 Hz, 1H), 6.81 – 6.78 (m, 1H), 2.94 – 2.89 (m, 2H), 2.78 – 2.67 (m, 3H), 2.51 (ddd, *J* = 18.9, 8.8, 0.9 Hz, 1H), 2.45 – 2.38 (m, 1H), 2.29 (td, *J* = 10.6, 3.8 Hz, 1H), 2.20 – 2.11 (m, 2H), 2.09 – 1.95 (m, 3H), 1.86 (dddd, *J* = 13.7, 8.6, 7.3, 6.1 Hz, 1H), 1.69 – 1.39 (m, 6H), 1.34 (d, *J* = 7.0 Hz, 3H), 0.92 (s, 3H).

^{13}C NMR (126 MHz, $CDCl_3$) δ 220.9, 175.4, 148.8, 141.6, 138.1, 137.4, 128.6, 126.5, 126.1, 121.6, 118.8, 50.5, 48.0, 44.3, 39.2, 38.1, 36.0, 35.6, 33.6, 31.6, 29.5, 26.4, 25.9, 21.7, 17.2, 13.9.

HRMS: (ESI^+) calculated for $C_{29}H_{34}NaO_3^+$ [M+Na$^+$]: 453.2400, found 453.2411.

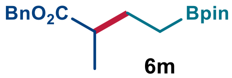
6m

Benzyl-2-methyl-4-(4,4,5,5-tetramethyl-1,3,2-dioxaborolan-2-yl)butanoate (6m): Synthesized according to the General Procedure using 3DPA2FBN (6.4 mg, 5.0 mol%), HAT **4c** (Ethyl thioglycolate, 4.4 µL, 0.04 mmol, 20 mol%), 2,4,6-collidine (26.5µL, 0.2 mmol, 1 equiv.), γ-terpinene (32.0 µL, 0.2 mmol, 1 equiv.), benzyl acrylate (30.5µL, 0.2 mmol, 1 equiv.) and vinylboronic acid pinacol ester (102.0 µL, 0.6 mmol, 3 equiv.). Upon 14 hours stirring, the crude mixture was purified by flash column chromatography on silica gel (2% acetone in hexanes as eluent) to afford **6m** (33.0 mg, 52% yield) as a yellow oil.

^{1}H NMR (400 MHz, $CDCl_3$) δ 7.39 – 7.27 (m, 5H), 5.10 (s, 2H), 2.51 – 2.43 (m, 1H), 1.79 (dq, *J* = 13.6, 7.8 Hz, 1H), 1.58 – 1.52 (m, 1H), 1.23 (s, 12H), 1.16 (d, *J* = 7.0 Hz, 3H), 0.78 (t, *J* = 8.3 Hz, 2H).

^{13}C NMR (101 MHz, $CDCl_3$) δ 176.7, 136.4, 128.6, 128.1, 128.1, 83.2, 66.0, 41.6, 28.2, 24.9, 24.9, 16.8.
HRMS: (ESI^+) calculated for $C_{18}H_{27}NaO_4B^+$ [M+Na$^+$]: 340.1931, found 340.1931.

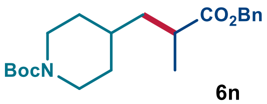

6n

***Tert*-butyl-4-(3-(benzyloxy)-2-methyl-3-oxopropyl)piperidine-1-carboxylate (6n)**: Synthesized according to the General Procedure using 3DPA2FBN (6.4 mg, 5.0 mol%), HAT **4c** (ethyl thioglycolate, 4.4 µL, 0.04 mmol, 20 mol%), 2,4,6-collidine (26.5 µL, 0.2 mmol, 1 equiv.), γ-terpinene (32.0 µL, 0.2 mmol, 1 equiv.), benzyl acrylate (30.5µL, 0.2 mmol, 1 equiv.) and *tert*-butyl 4-methylenepiperidine-1-carboxylate (118.5 µL, 0.6 mmol, 3 equiv.). Upon 14 hours stirring, the crude mixture was purified by flash column chromatography on silica gel (10% EtOAc in hexanes as eluent) to afford **6n** (35.5 mg, 49% yield) as a colorless oil.

^{1}H NMR (500 MHz, $CDCl_3$) δ 7.40 – 7.30 (m, 5H), 5.18 – 5.06 (m, 2H), 4.02 (s, 2H), 2.66 – 2.38 (m, 3H), 1.69 – 1.52 (m, 3H), 1.44 (s, 9H), 1.35 – 1.28 (m, 2H), 1.16 (d, J = 6.9 Hz, 3H), 1.06 – 0.99 (m, 2H).

^{13}C NMR (126 MHz, $CDCl_3$) δ 176.7, 154.9, 136.2, 128.7, 128.3, 79.4, 66.2, 40.8, 36.9, 33.9, 32.0, 29.8, 28.6, 17.8.

HRMS: (ESI^+) calculated for $C_{21}H_{31}NNaO_4^+$ [$M+Na^+$]: 384.2145, found 384.2140.

F. Reaction Scale-Up

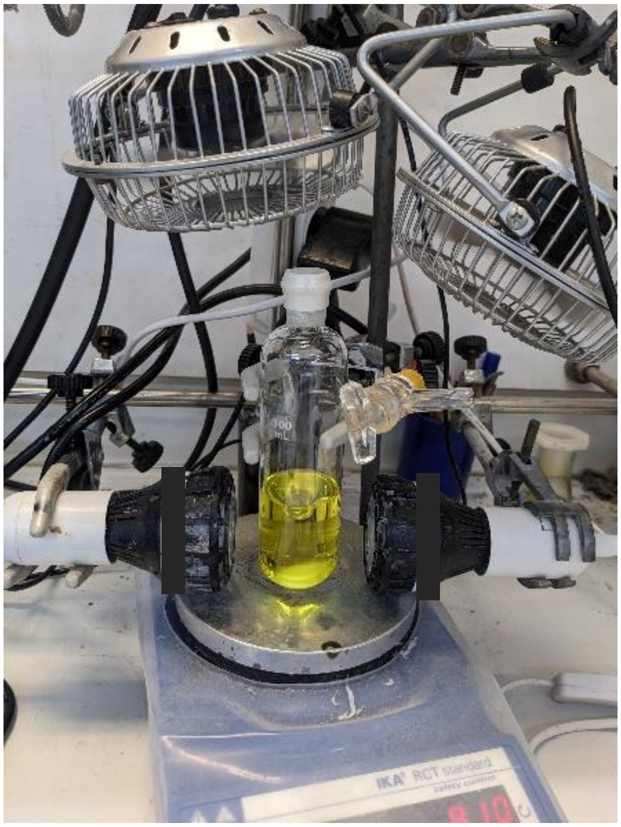

Figure S3. 5 mmol scale reaction between **1a** and **2a** using 2 EvoluChem 350 nm lamps.

An oven-dried 100 mL Schlenk flask containing a Teflon stir bar and dimethyl fumarate **1a** (1.08 g, 7.5 mmol, 1.5 equiv.) was sealed with a rubber septum and purged with argon. 1,2-Dichloroethane (45 ml, HPLC grade) was added followed by 5 ml of *fac*-Ir(ppy)$_3$ stock solution (prepared according to the General Procedure; 1.0 mg/mL in DCE, 5 mg, 0.15 mol%), styrene (572 µL, 5.0 mmol, 1 equiv.), γ-terpinene (1.60 ml, 10 mmol, 2 equiv.), and thiophenol (102 µL, 1.0 mmol, 20 mol%). The reaction vessel was placed into a photoreactor setup as depicted on the Figure S3 with both lamps and fans running. Under these conditions, the internal temperature of the reaction reached 55-60 °C due to the heat generated from light absorption (with an ambient temperature of 22 °C). The consumption of dimethyl fumarate was monitored by taking an aliquot (50 µL) with a syringe and performing GC-MS analysis. After 84 hours, upon full consumption of dimethyl fumarate **1a**, the reaction mixture was allowed to cool down, washed with 2x20 mL saturated Na_2CO_3 solution and 20 mL of saturated NaCl solution and dried over Na_2SO_4. This solution was filtered through a silica plug, washing with DCM (ca. 300 mL). The filtrate was evaporated and purified by silica gel chromatography (10% EtOAc in hexane). Fractions were evaporated and the material dried on high vacuum (pressure 2.5*10^-1 mbar) at 40 °C to remove dimethyl succinate impurity yielding 1005 mg of **3a** (80% yield) as a colorless oil.

G. Additional experimental information

G1. Unsuccessful substrates

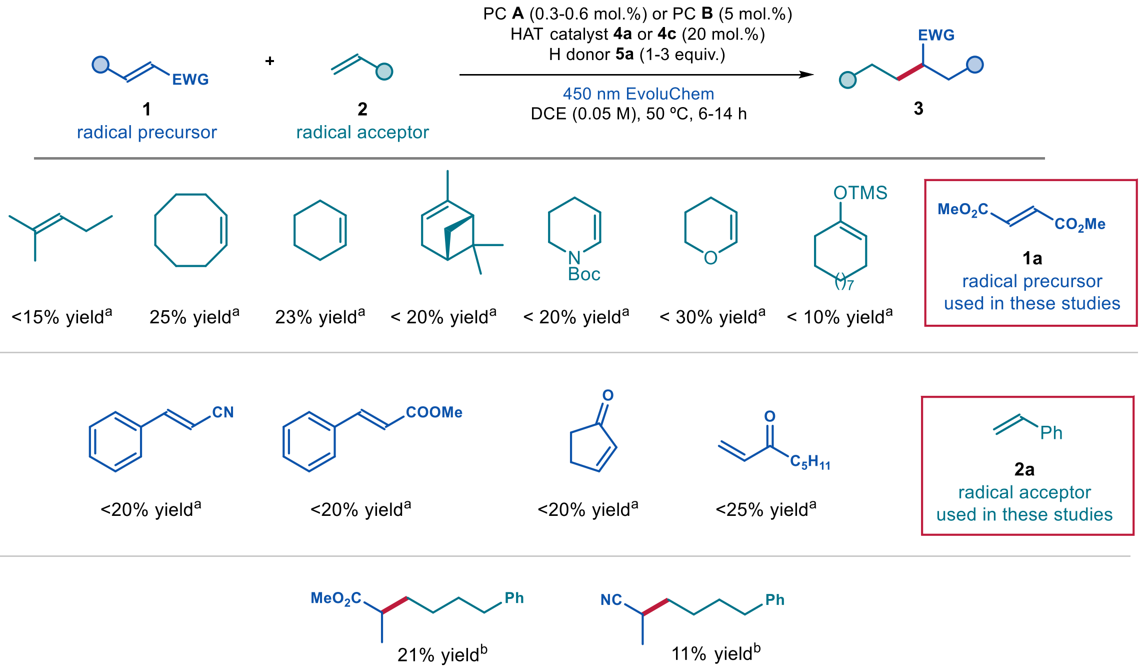

[a]Yield determined by 1H NMR analysis of the crude mixture using dibromomethane as the internal standard.
[b]Yield determined by GC-FID analysis of the crude reaction mixture using 1,3,5-trimethoxybenzene as the internal standard.

Figure S4. Unsuccessful and poorly reactive substrates.

Among the tested substrates, internal olefins showed poor reactivity, typically resulting in the formation of products but with unsatisfactory yields. Enones, whether cyclic or terminal, also exhibited poor reactivity under all tested conditions. When attempting cross-coupling between poorly activated electron-poor olefins and monosubstituted terminal olefins, the yields were low.

G2. Major byproducts

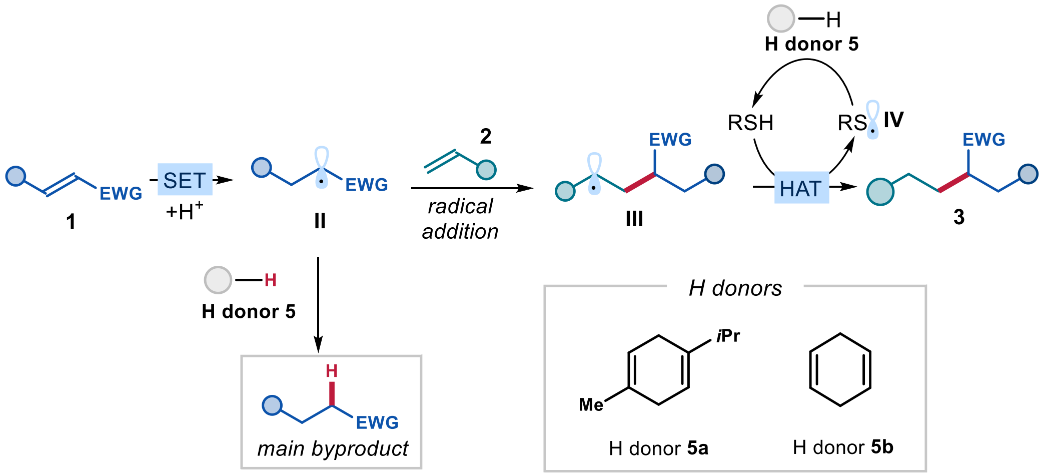

Figure S5. Proposed mechanism of byproduct formation.

In this protocol, we have only observed one major byproduct, which is the product resulting from the reduction of electron-poor olefins **1**, arising from a competitive HAT process involving radical **II** and H donor **5**.

G3. Optimization of equivalents

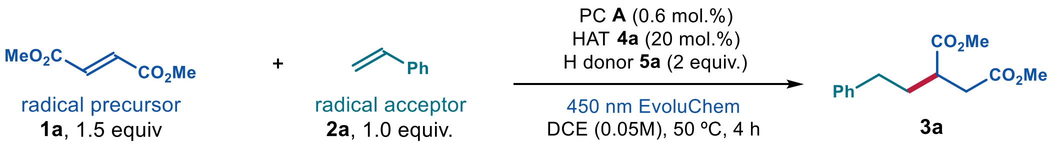

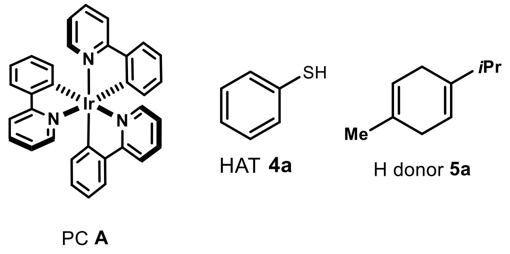

entry	deviation	yield (%)[a]
1	-	95 (83)
2	2.0 equiv. **1a**	89
3	1.2 equiv. **1a**	83
4	1.5 equiv. **5a**	91
5	1.2 equiv. **5a**	88

Table S1. Optimization of equivalents of **1a** and **5a** for model reaction (0.1 mmol scale). [a]Yield of **3a** determined by GC-FID analysis of the crude reaction mixture using 1,3,5-trimethoxybenzene as the internal standard. Yields of isolated **3a** are reported in parentheses (0.2 mmol scale).

H. Mechanistic Experiments

H1. Stern-Volmer Quenching Studies

The absorption spectra of *fac*-Ir(ppy)$_3$ (PC **A**) was recorded (Figure S6) in order to establish appropriate concentration and excitation wavelength. The spectra was recorded using a Shimadzu 2401PC UV−vis spectrophotometer and 10 mm path quartz cuvette. The concentration of 1.5 µM in DCE, and excitation wavelength of 440 nm were chosen for Stern-Volmer studies based on these results (absorbance value of 0.1 at 440 nm in these conditions).

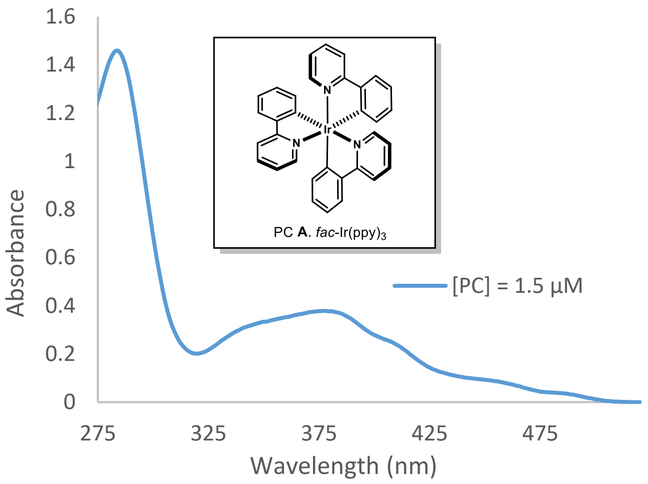

Figure S6. Absorption spectrum of PC **A** (1.5 µM in DCE, 10 mm light path).

The emission spectra was recorded in a Fluorolog Horiba Jobin Yvon spectrofluorimeter equipped with a photomultiplier detector, a double monochromator, and a 350W xenon light source. 2 mL of a 1.5 µM solution of PC **A** in thoroughly degassed DCE was placed in a 10x10 mm light path quartz fluorescence cuvette equipped with Silicone/PTFE 3.2 mm septum under an argon atmosphere. The excitation wavelength was fixed at 440 nm (incident light slit regulated to 1.5 nm), while the emission signal was acquired from 455 nm to 650 nm (emission light slit regulated to 1.5 nm).

A 0.5 M solution of dimethyl fumarate **1a** in DCE was prepared, and 5 µL of this stock solution were added to the solution of the catalyst. The addition of this solution was repeated four consecutive times. After each addition, an absorption spectrum and an emission spectrum of the solution were recorded. The results shown in Figure S7 indicate that dimethyl fumarate **1a** quenches the excited state of the catalyst **PC**. No change in the absorption spectra of the solution was observed during the addition of **1a**.

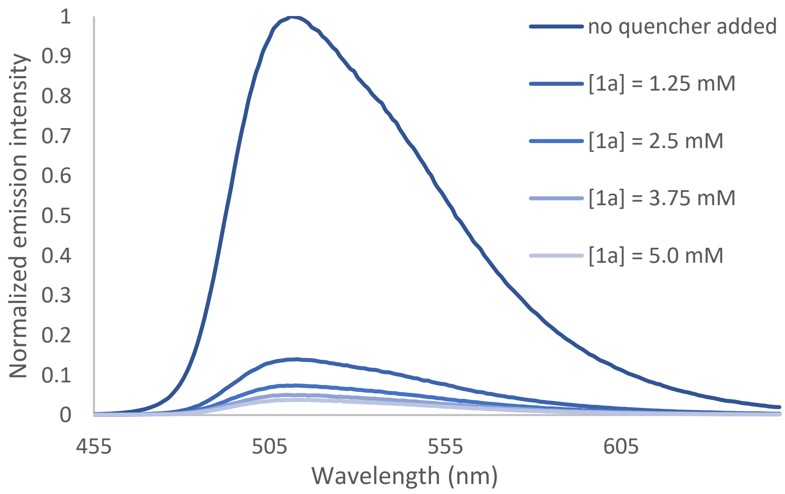

Figure S7. Quenching of the emission of **PC** (1.5 µM in DCE) in the presence of increasing amounts of **1a**.

The Stern-Volmer plot, reported in Figure S8, shows a linear correlation between the concentration of **1a** and the ratio I_0/I. On the basis of the Stern-Volmer equation $I_0/I = 1 + K_{SV}[Q]$, we calculated a Stern-Volmer quenching constant $\mathbf{K_{SV} = 5068\ M^{-1}}$. A 0.5 M solution of styrene **2a** in DCE was prepared, and portions of this stock solution were added to the solution of the catalyst. The addition of this solution was repeated five consecutive times. After each addition, an absorption spectrum and an emission spectrum of the solution were recorded.

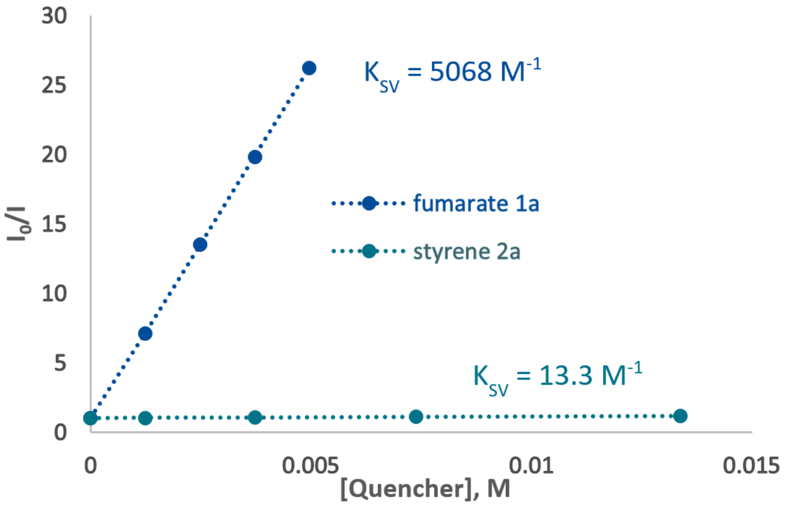

Figure S8. Stern-Volmer quenching plot using **1a** and **2a** as a quencher.

No change in the absorption spectra of the solution was observed during the addition of **2a**. The Stern-Volmer plot is reported in Figure S8. A much smaller Stern-Volmer constant was calculated in this case ($\mathbf{K_{SV} = 13.3\ M^{-1}}$).

H2. Radical clock experiment

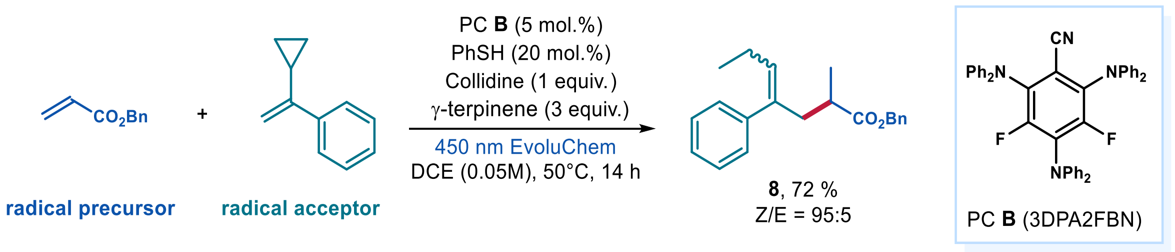

Figure S9. Radical clock experiment.

The radical clock experiment was performed according to the General Procedure using 3DPA2FBN (6.4 mg, 5.0 mol%), PhSH (4.1 µL, 0.04 mmol, 20 mol%), 2,4,6-collidine (26.4 µL, 0.2 mmol, 1 equiv.), γ-terpinene (96 µL, 0.6 mmol, 3 equiv.), benzyl acrylate (30.6 µL, 0.2 mmol, 1 equiv.) and α-cyclopropylstyrene (90.0 µL, 0.6 mmol, 3 equiv.). Upon 14 hours stirring, the crude mixture was

purified by flash column chromatography on silica gel (2% EtOAc in hexanes as eluent) to afford **8** (44.5 mg, 72% yield, 95:5 Z/E) as a colorless oil.

^{1}H NMR (500 MHz, $CDCl_3$, major) δ 7.39 – 7.28 (m, 7H), 7.25 – 7.21 (m, 1H), 7.14 – 7.10 (m, 2H), 5.46 (t, *J* = 7.4, 1H), 5.04 (s, 2H), 2.81 (ddq, *J* = 13.4, 6.9, 1.1 Hz, 1H), 2.49 – 2.40 (m, 1H), 2.39 (ddq, *J* = 13.4, 7.4, 0.8 Hz, 1H), 1.97 – 1.84 (m, 2H), 1.13 (d, *J* = 6.9 Hz, 3H), 0.90 (t, *J* = 7.5 Hz, 3H). ^{13}C NMR (126 MHz, $CDCl_3$, major) δ 176.3, 140.3, 137.4, 136.3, 131.9, 128.7, 128.6, 128.2, 128.2, 128.2, 126.8, 66.1, 43.4, 38.2, 22.4, 16.8, 14.7.

HRMS: (ESI$^+$) calculated for $C_{21}H_{24}NaO_2^+$ [M+Na$^+$]: 331.1669, found 331.1670.

H3. Deuterium labeling experiment

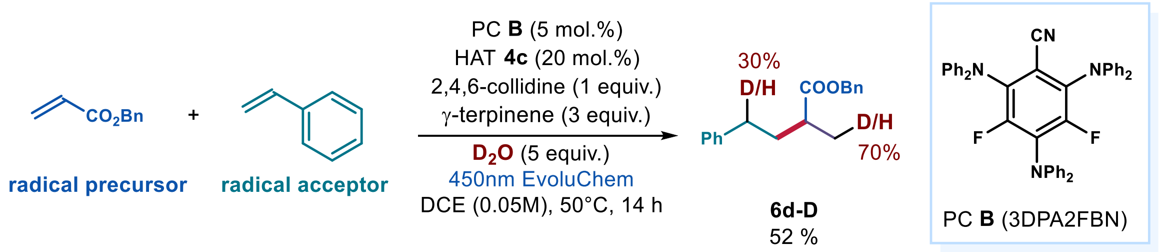

Figure S9. Deuterium labeling experiment.

To an 8 mL oven dried vial with a Teflon septum screw cap containing a dry Teflon stir bar, 3DPA2FBN (6.4 mg, 5.0 mol%) was added. The vial was sealed, evacuated and backfilled with argon three times, and the solvent was added. Then PhSH (4.1 µL, 0.04 mmol, 20 mol%), 2,4,6-collidine (26.4 µL, 0.2 mmol, 1 equiv.), γ-terpinene (96 µL, 0.6 mmol, 3 equiv.), benzyl acrylate **(**30.6µL, 0.2 mmol, 1 equiv.), styrene **2a** (68.7 µL, 0.6 mmol, 3 equiv.) and **D_2O** (18.1 µL, 1.0 mmol, 5 equiv.) were added sequentially. The vial was sonicated for 1 minute and was then placed in the photoreactor (Figure S1) and irradiated with vigorous stirring for 14 hours. The solvent was evaporated and the crude mixture purified by flash column chromatography on silica gel (2% EtOAc in hexanes as eluent) to furnish the target product **6d-D (**27.9 mg, 52% yield) as a colorless oil. Deuteration ratio was reported based on integration of peaks in ^{13}C IGD (inverse-gated-decoupling) experiment. ^{13}C signals were assigned by analyzing HSQC (heteronuclear single quantum coherence) spectrum.

^{1}H NMR (500 MHz, $CDCl_3$) δ 7.41 – 7.33 (m, 5H), 7.30 – 7.25 (m, 2H), 7.22 – 7.17 (m, 1H), 7.15 (ddt, *J* = 7.7, 1.4, 0.7 Hz, 2H), 5.15 (s, 2H), 2.62 – 2.60 (m, 1.6H), 2.58 – 2.50 (m, 1H), 2.09 – 2.00 (m, 1H), 1.79 – 1.72 (m, 6.1 Hz, 1H), 1.25 – 1.19 (m, 2.3H).

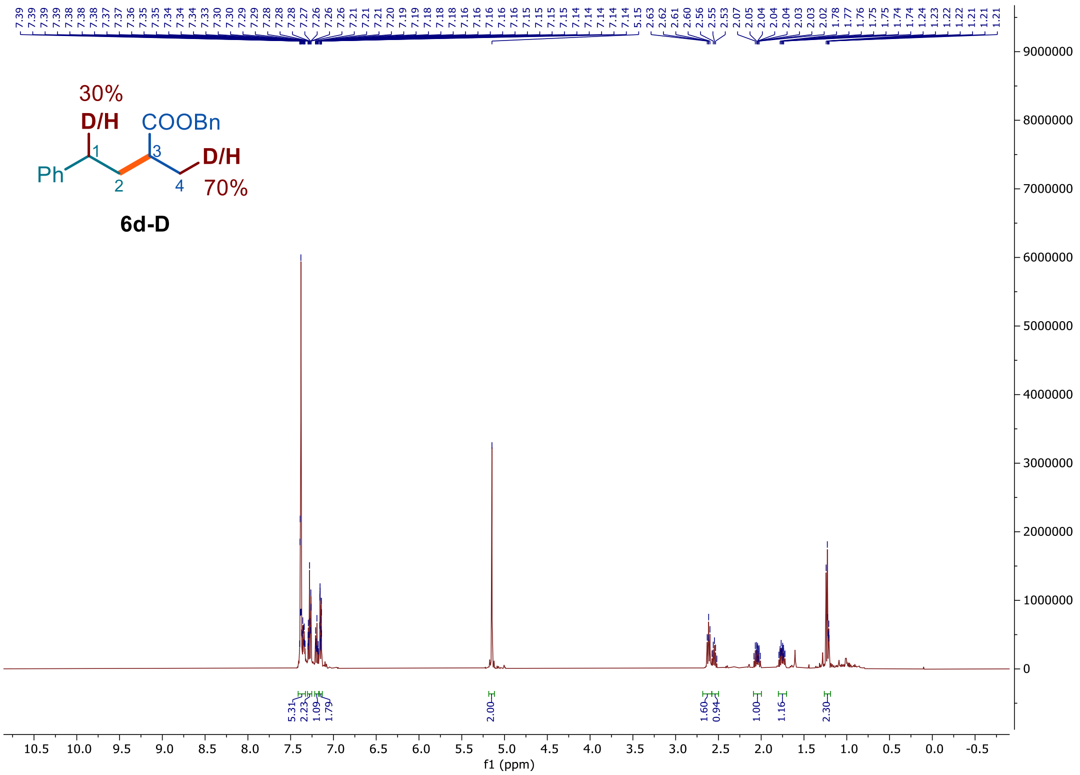

^{13}C NMR (126 MHz, $CDCl_3$) δ 176.43, 141.75, 136.31, 128.68, 128.54, 128.48, 128.30, 128.28, 126.02, 66.21, (39.20; 39.18; 39.13; 39.11: 1C), (35.57; 35.54; 35.49; 35.46: 1C), (33.55; 33.19 (t, J = 19.5 Hz): 1C), (17.27; 16.99 (t, J = 19.5 Hz): 1C).

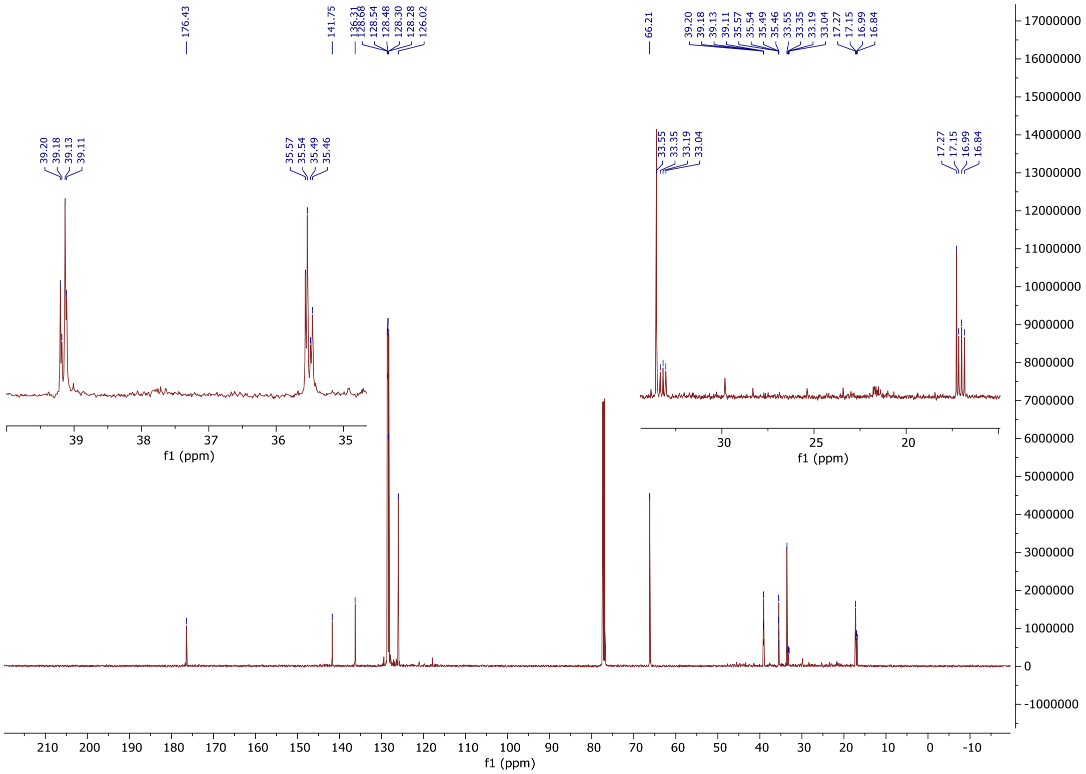

^{13}C NMR (126 MHz, $CDCl_3$, IGD, 10s delay) δ 33.55 (0.7C); 33.19 (t, *J* = 19.5 Hz, 0.3C), 17.27 (0.3C); 16.99 (t, *J* = 19.5 Hz, 0.7C).

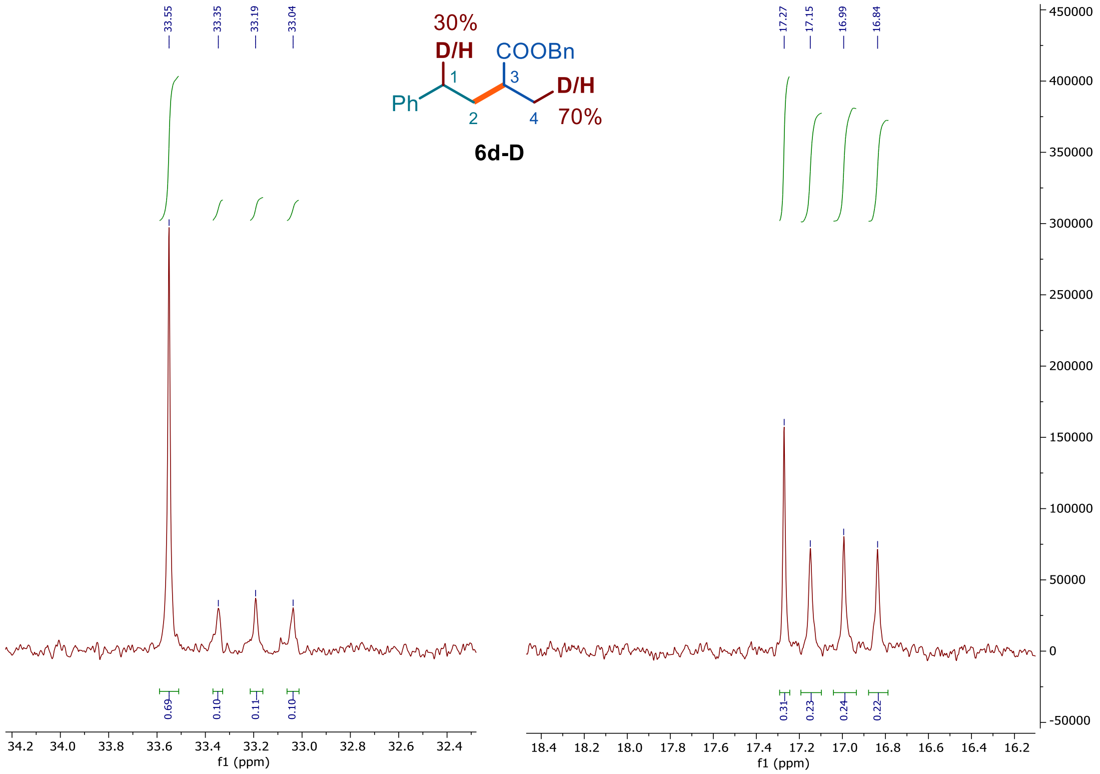

^{2}H NMR (77 MHz, $CHCl_3$; $CDCl_3$ added as internal reference) δ 2.61 (s, 1D), 1.24 (s, 2.47D).

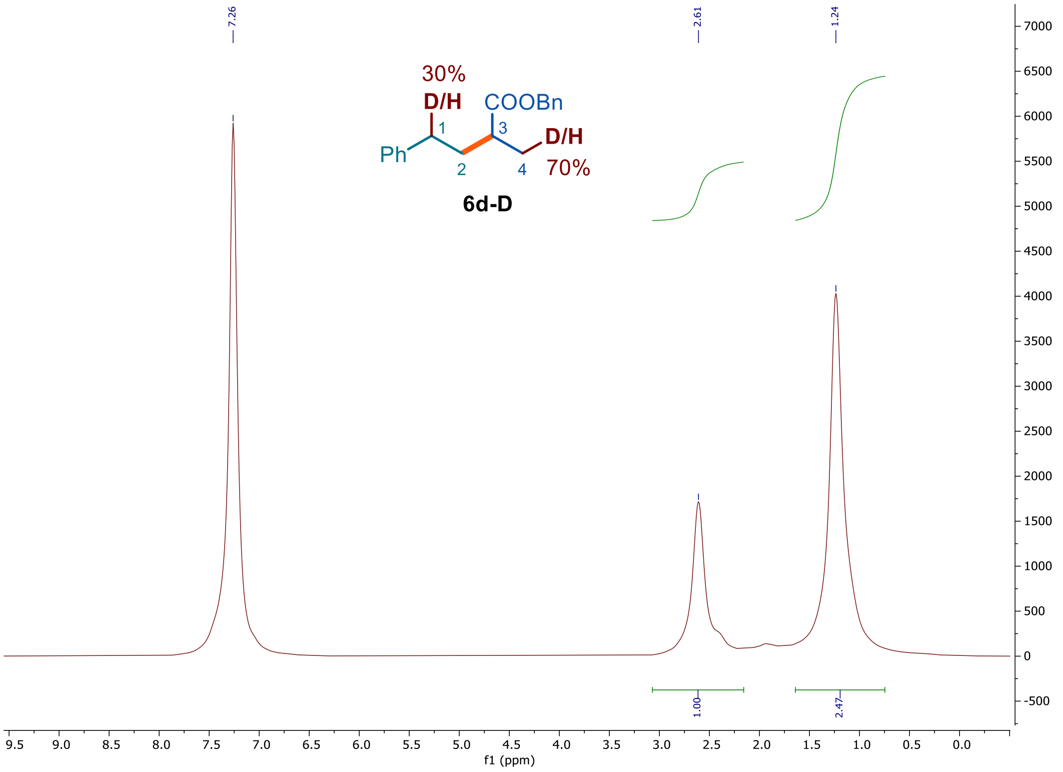

HSQC

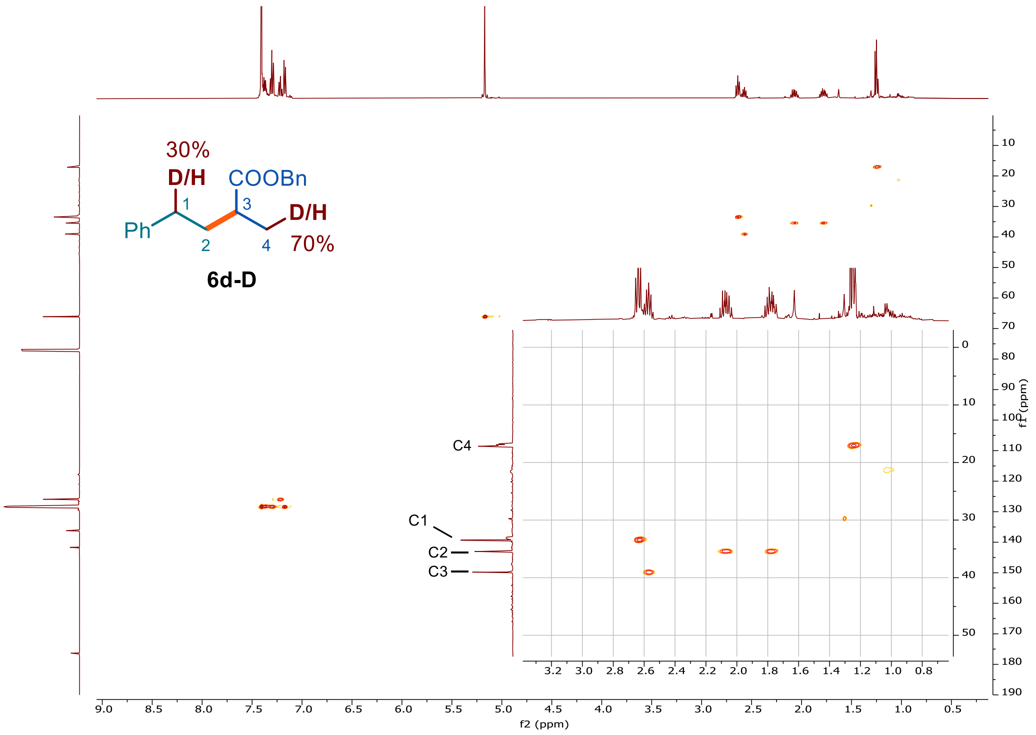

H4. Mechanistic rationalization for less activated radical precursors

For the more activated substrates, such as dimethyl fumarate (E (**1a**/**1a•−**) = -1.47 V vs SCE), the model reaction conditions using photocatalyst **A** (*fac*-Ir(ppy)$_3$, *E**[(Ir(IV)/Ir(III)*] = -1.88 V vs SCE) proved to be effective. Our mechanistic studies, as detailed in the main manuscript (Figure 2) and Section H1 of the SI, support this *oxidative quenching pathway*, where the excited photocatalyst **A** reduces **1a** via SET.

When dealing with less activated substrates, including benzyl acrylate (E = -2.2 V vs SCE)[16], the reaction did not progress with Photocatalyst **A** and required modified conditions (Photocatalyst **B** + collidine). Our mechanistic studies, performed with benzyl acrylate, are consistent with a pathway that proceeds through the formation of the same intermediary radical-anion of type **I**, which is then protonated to form the radical of type **II**. Specifically, the radical clock experiment and the deuterium-labeling studies presented in Figure 4 of the main manuscript and Sections H2 and H3 of the SI support this mechanism.

We propose that the new conditions (PC **B** + collidine) are suitable for reactivity for the following reason: due to the mild basicity of collidine (pK_a = 7.5), a dynamic equilibrium between thiophenol (pK_a = 6.6) and thiophenolate is established in the solution. Photocatalyst **B** ($E_{1/2}$(PC*/PC$^{•−}$) = +0.92V) is capable of oxidizing thiophenolate (E_{ox} = +0.07 V vs SCE). In this process, the radical-anion PC **B**$^{•−}$ ($E_{1/2}$(PC **B**/PC **B**$^{•−}$) = -1.92 V) is generated. This species is more poised to reduce the less activated electron-poor olefins, including benzyl acrylate, providing the radical-anion of type **I**, which starts the productive pathway. Overall, according to this mechanistic picture, the reaction would proceed via a *reductive quenching pathway* as depicted in the Figure S10 below.

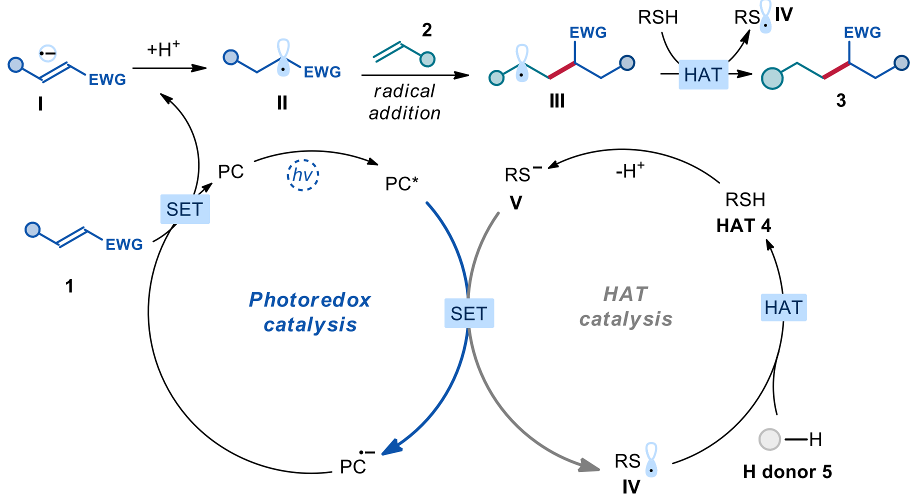

Figure S10. Reductive quenching manifold *with less activated electron-poor olefins* (benzyl acrylate is shown as an example).

I. References

1) Askey, H. E.; Grayson, J. D.; Tibbetts, J. D.; Turner-Dore, J. C.; Holmes, J. M.; Kociok-Kohn, G.; Wrigley, G. L.; Cresswell, A. J., Photocatalytic Hydroaminoalkylation of Styrenes with Unprotected Primary Alkylamines. *J. Am. Chem. Soc.* **2021**, *143*, 15936−15945.

2) Agudo, R.; Roiban, G.-D.; Reetz, M. T. Achieving Regio- and Enantioselectivity of P450-Catalyzed Oxidative CH Activation of Small Functionalized Molecules by Structure-Guided Directed Evolution. *ChemBioChem* **2012**, *13*, 1465−1473.

3) Henßen B., Kasparyan E., Marten G., and Pietruszka J. Synthesis of Psymberic Acid. *Heterocycles* **2007**, *74*, 245−249.

4) Choudhary, S.; Cannas, D. M.; Wheatley, M.; Larrosa, I. A manganese(i)tricarbonyl-catalyst for near room temperature alkene and alkyne hydroarylation. *Chem. Sci.* **2022**, *13*, 13225−13230.

5) Hell, S. M.; Meyer, C. F.; Laudadio, G.; Misale, A.; Willis, M. C.; Noël, T.; Trabanco, A. A.; Gouverneur, V., Silyl Radical-Mediated Activation of Sulfamoyl Chlorides Enables Direct Access to Aliphatic Sulfonamides from Alkenes. *J. Am. Chem. Soc.* **2020**, *142*, 720−725.

6) Huang J., Zhao R., Qin S., Yang S., Li W., Mo J., Du Y., Weng X., Zhou X., 4-Thiouridine-Enhanced Peroxidase-Generated Biotinylation of RNA. *ChemBioChem* **2020**, *22*, 212−216

7) Fini, F.; Beltrani, M.; Mancuso, R.; Gabriele, B.; Carfagna, C., Selective Aryl α-Diimine/Palladium-Catalyzed Bis-Alkoxy- carbonylation of Olefins for the Synthesis of Substituted Succinic Diesters. *Adv. Synth. Catal.* **2015**, *357*, 177-184.

8) Zhang, Y.; Han, Y.; Zhu, S.; Qing, F.-L.; Xue, X.-S.; Chu, L., Light-Induced Divergent Cyanation of Alkynes Enabled by Phosphorus Radicals. *Angew. Chem., Int. Ed.* **2022**, *61*, e202210838.

9) Gaspar, B.; Carreira, E. M., Mild Cobalt-Catalyzed Hydrocyanation of Olefins with Tosyl Cyanide. *Angew. Chem., Int. Ed.* **2007**, *46*, 4519−4522.

10) Ren, W.; Wang, M.; Guo, J.; Zhou, J.; Chu, J.; Shi, Y.; Shi, Y., Pd-Catalyzed Regioselective Branched Hydrocarboxylation of Terminal Olefins with Formic Acid. *Org. Lett.* **2022**, *24*, 886−891.

11) Schoenebeck, F.; Murphy, J. A.; Zhou, S.-z.; Uenoyama, Y.; Miclo, Y.; Tuttle, T., Reductive Cleavage of Sulfones and Sulfonamides by a Neutral Organic Super-Electron-Donor (S.E.D.) Reagent. *J. Am. Chem. Soc.* **2007**, *129*, 13368−13369.

12) Sharma, S.; Han, S. H.; Jo, H.; Han, S.; Mishra, N. K.; Choi, M.; Jeong, T.; Park, J.; Kim, I. S., Rhodium-Catalyzed Vinylic C–H Functionalization of Enol Carbamates with Maleimides. *Eur. J. Org. Chem.* **2016**, 3611−3618.

13) Capacci, A. G.; Malinowski, J. T.; McAlpine, N. J.; Kuhne, J.; MacMillan, D. W. C., Direct, enantioselective α-alkylation of aldehydes using simple olefins. *Nat. Chem.* **2017**, *9*, 1073−1077.

14) Ling, J.; Bruneau-Voisine, A.; Journot, G.; Evano, G., Copper-Catalyzed Carbonylative Cross-Coupling of Alkyl Iodides and Amines. *Chem. Eur. J.* **2022**, *28*, e202201356.

15) Miao, P.; Li, R.; Lin, X.; Rao, L.; Sun, Z., Visible-light induced metal-free cascade Wittig/hydroalkylation reactions. *Green Chemistry* **2021**, *23*, 1638−1641.

16) Matsunami, M.; Sakai, N.; Morimoto, T.; Maekawa, H.; Nishiguchi, I., Magnesium-Promoted One-Pot Double C-Acylation and Cycloaddition of Anthracene and Double C-Acylation of Benzyl Acrylates. *Synlett* **2007**, 5, 769−774.

J. NMR spectra

^{1}H NMR (500 MHz, $CDCl_3$) of **2s**

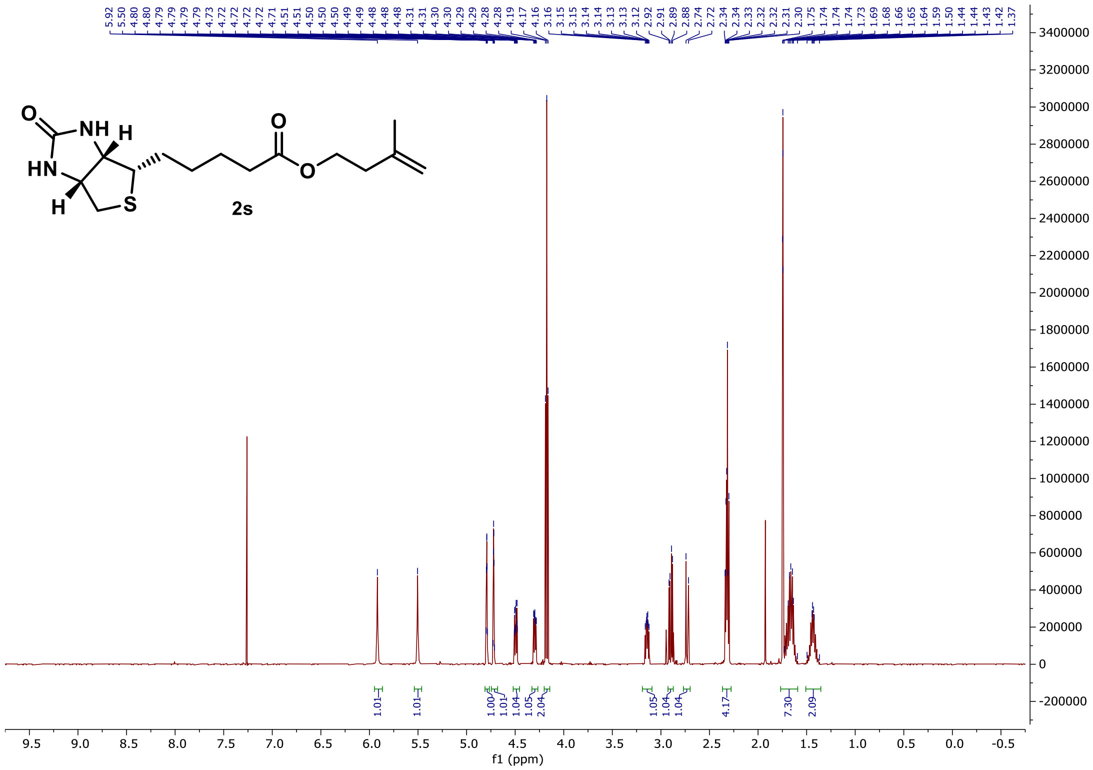

^{13}C NMR (126 MHz, $CDCl_3$) of **2s**

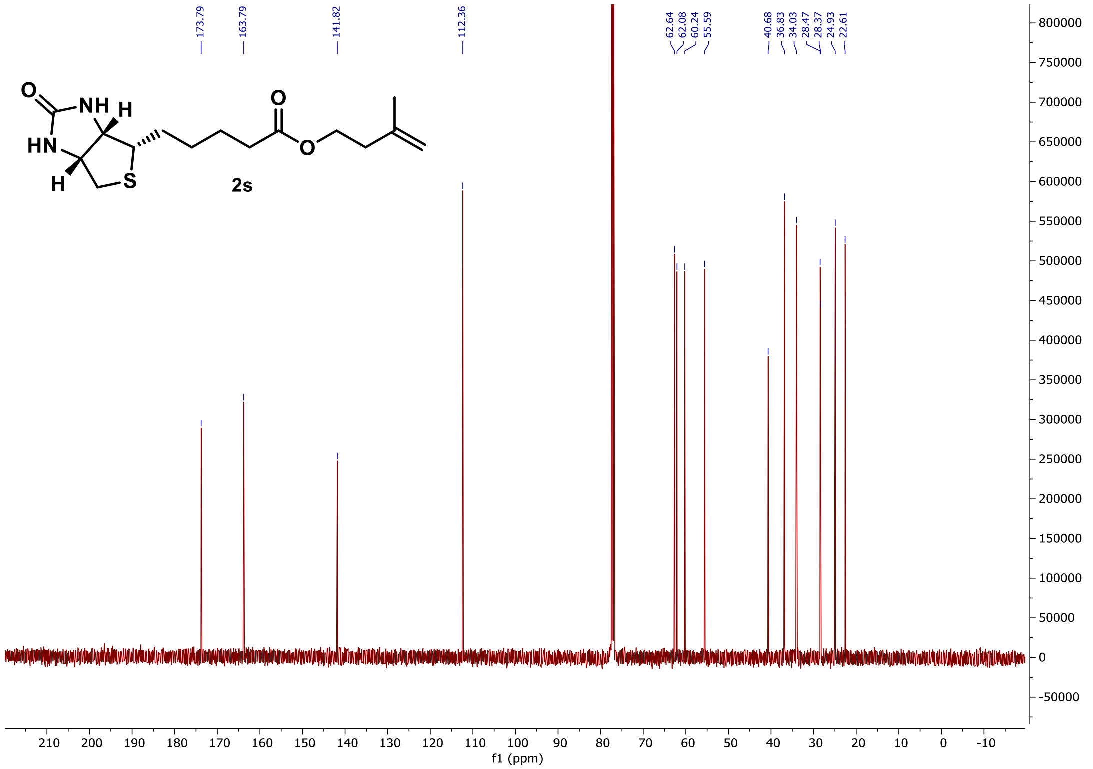

^{1}H NMR (500 MHz, CDCl$_3$) of **2u**

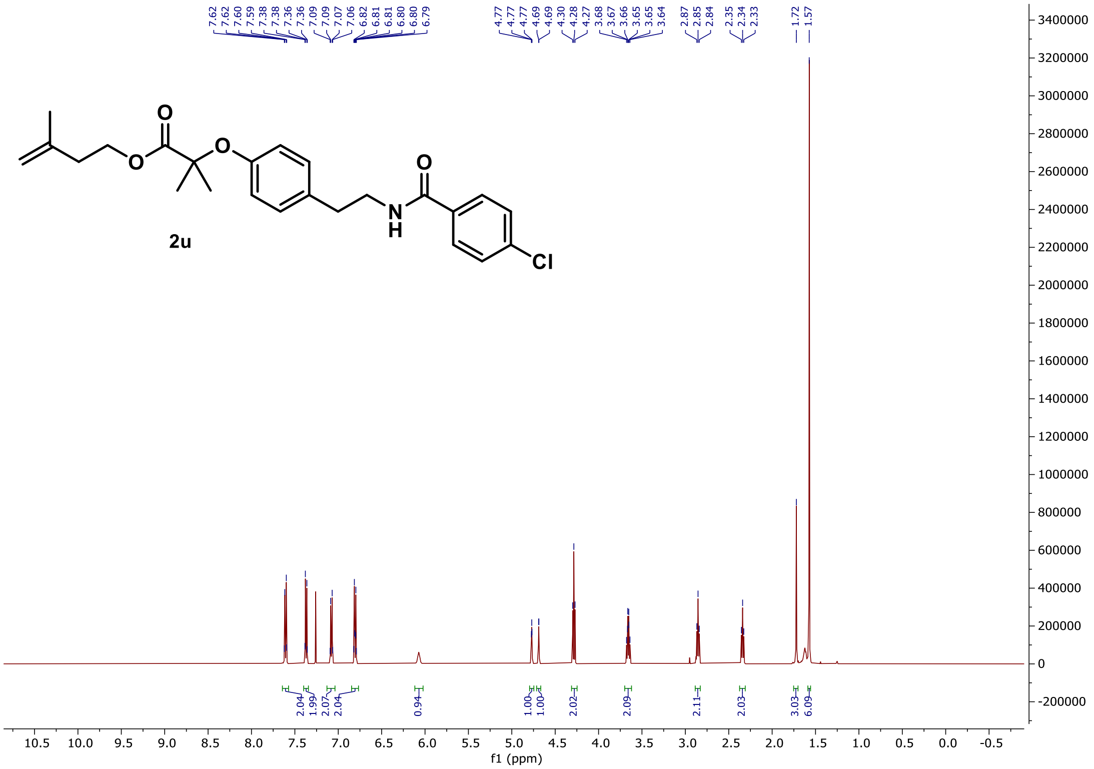

^{13}C NMR (126 MHz, CDCl$_3$) of **2u**

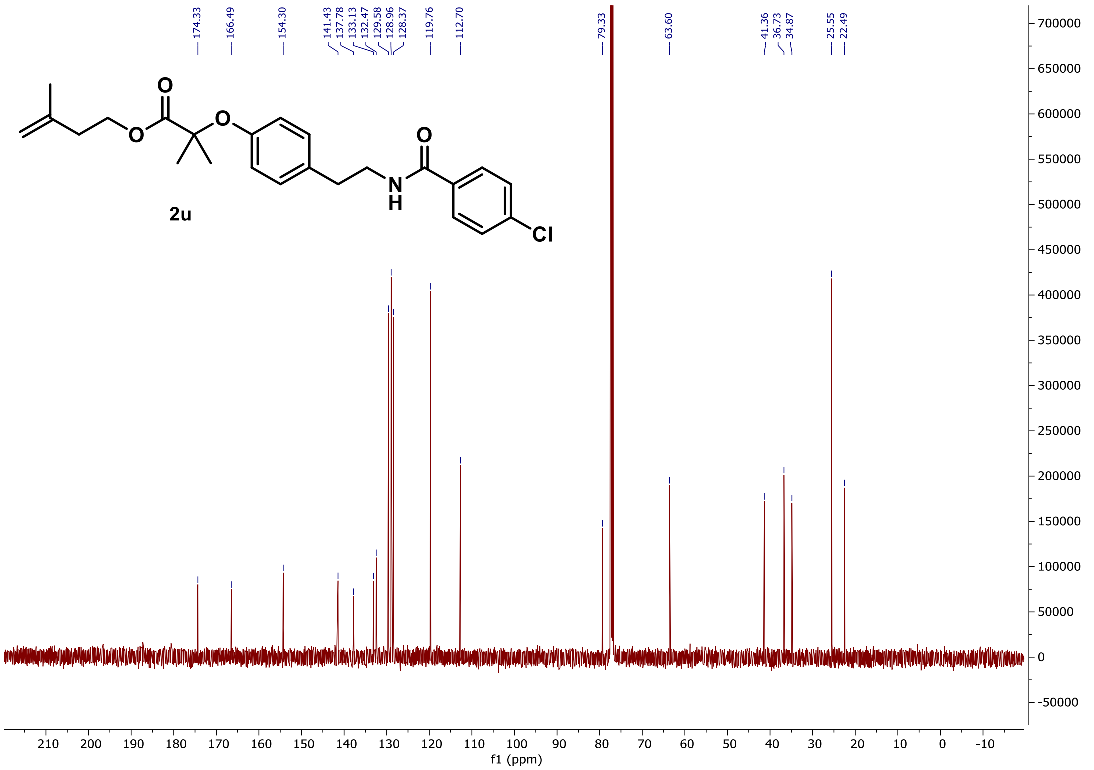

^{1}H NMR (500 MHz, $CDCl_3$) of **3a**

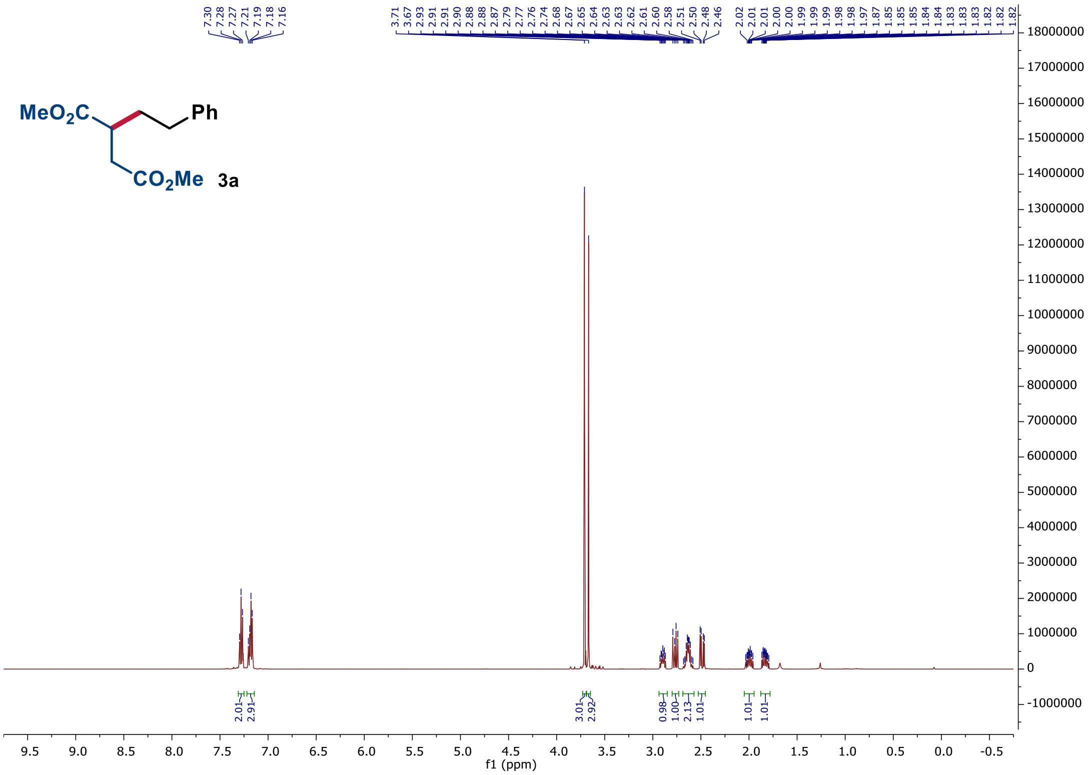

^{13}C NMR (126 MHz, $CDCl_3$) of **3a**

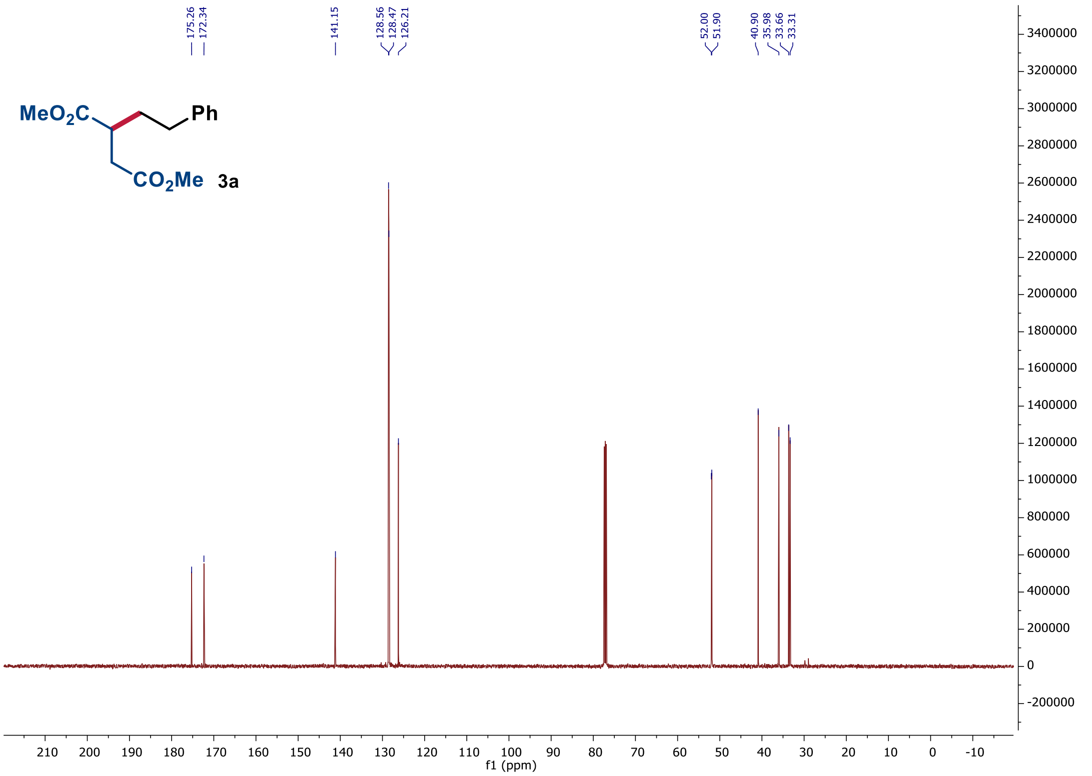

^{1}H NMR (500 MHz, $CDCl_3$) of **3b**

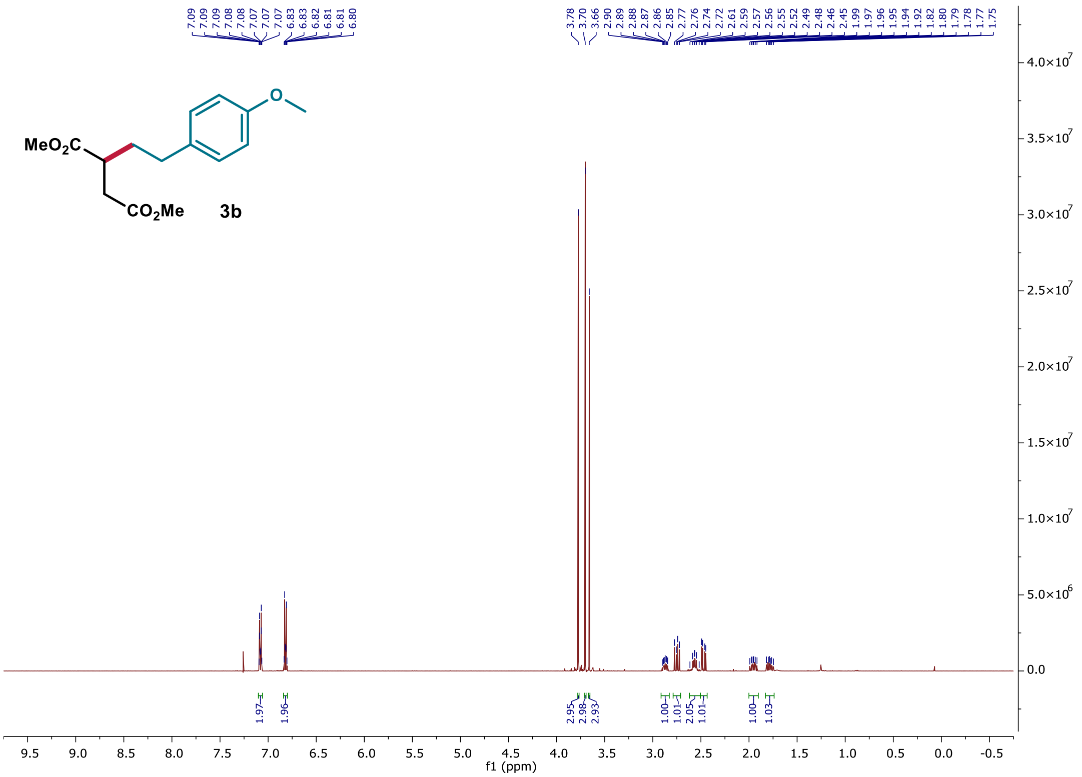

^{13}C NMR (126 MHz, $CDCl_3$) of **3b**

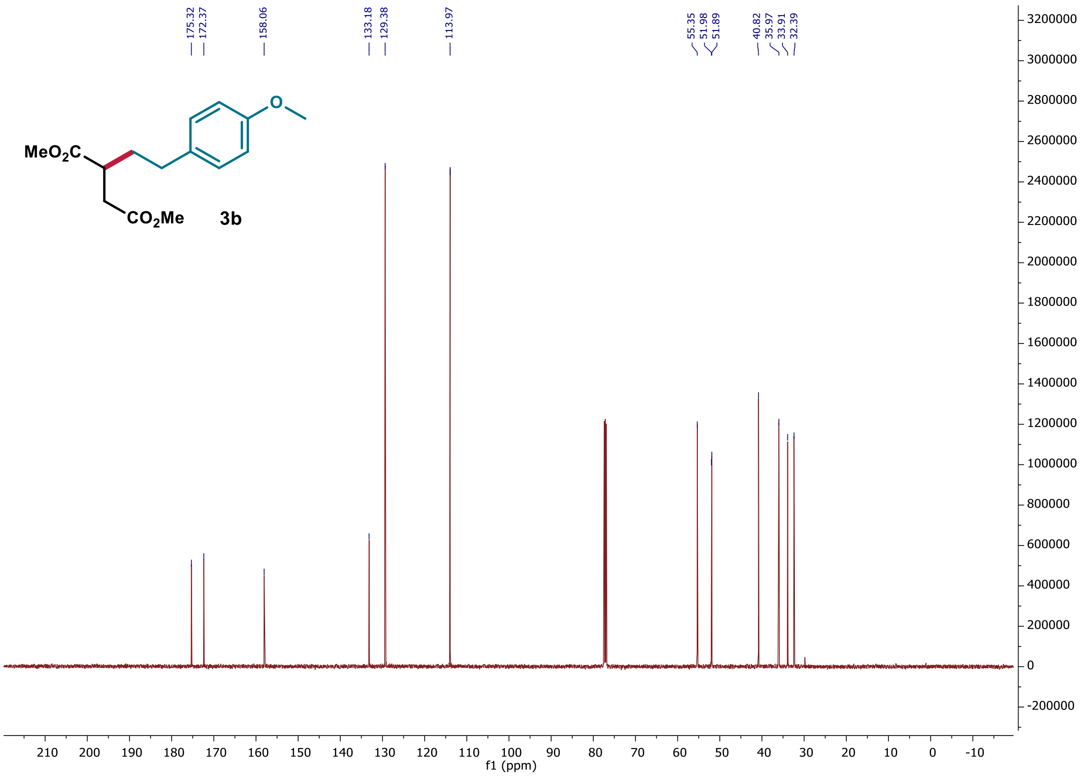

^{1}H NMR (500 MHz, $CDCl_3$) of **3c**

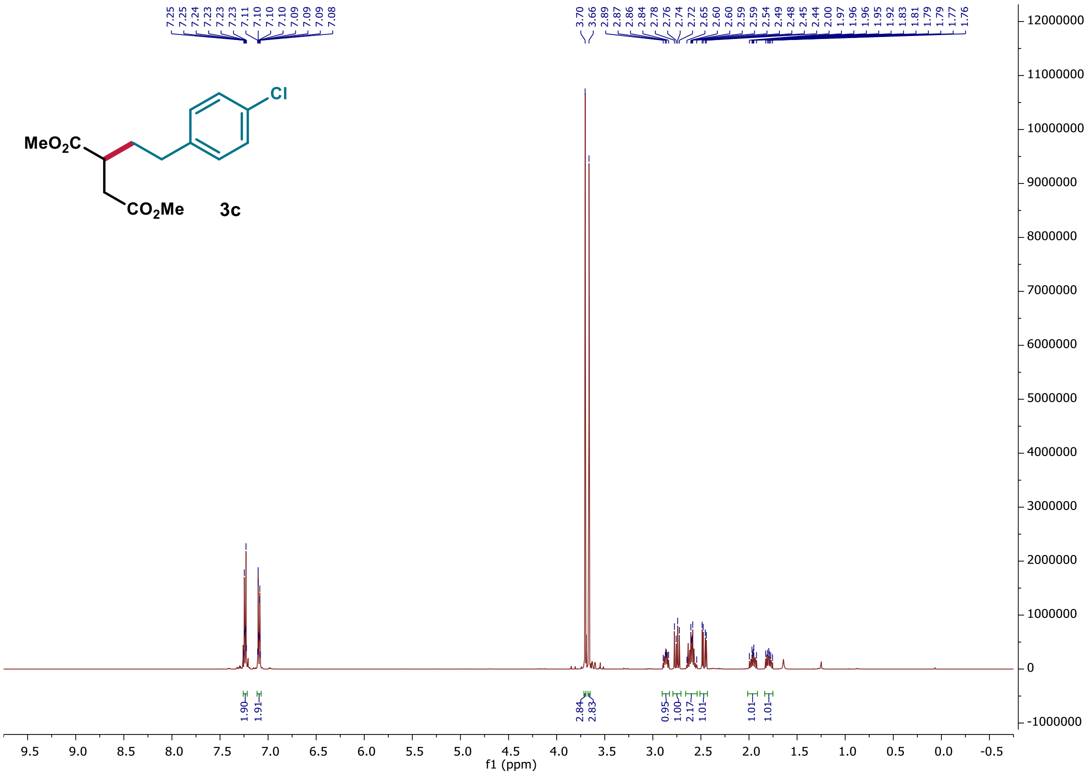

^{13}C NMR (126 MHz, $CDCl_3$) of **3c**

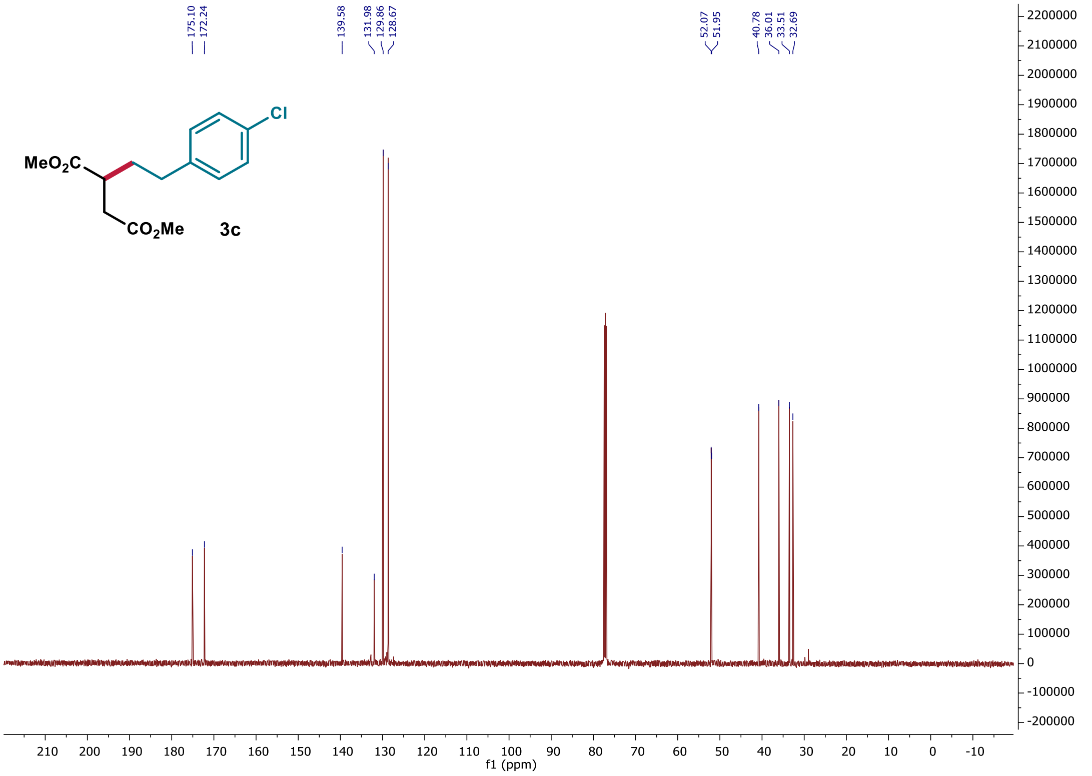

^{1}H NMR (500 MHz, $CDCl_3$) of **3d**

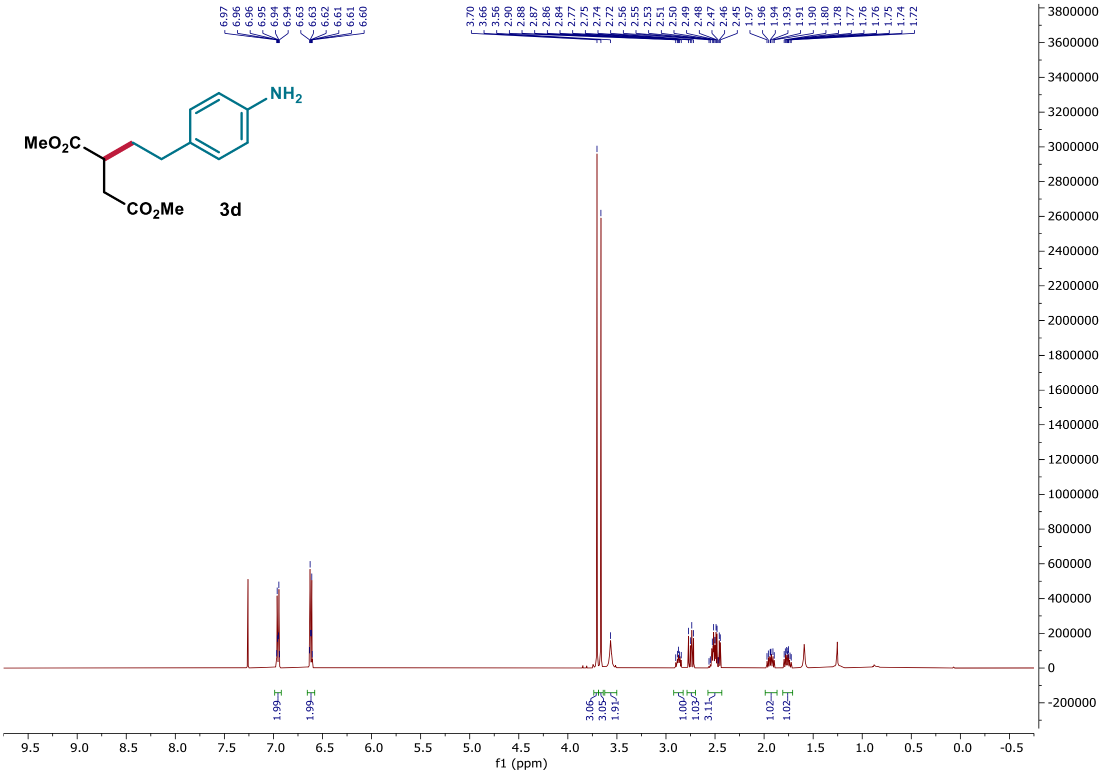

^{13}C NMR (126 MHz, $CDCl_3$) of **3d**

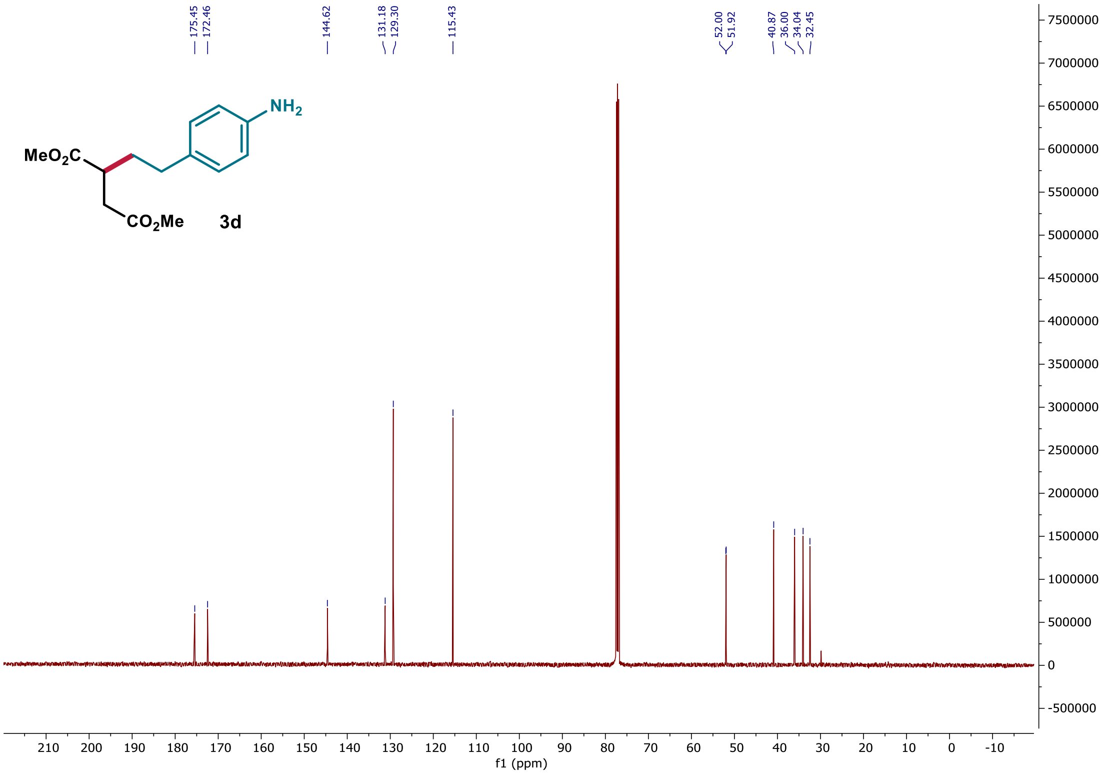

^{1}H NMR (500 MHz, $CDCl_3$) of **3e**

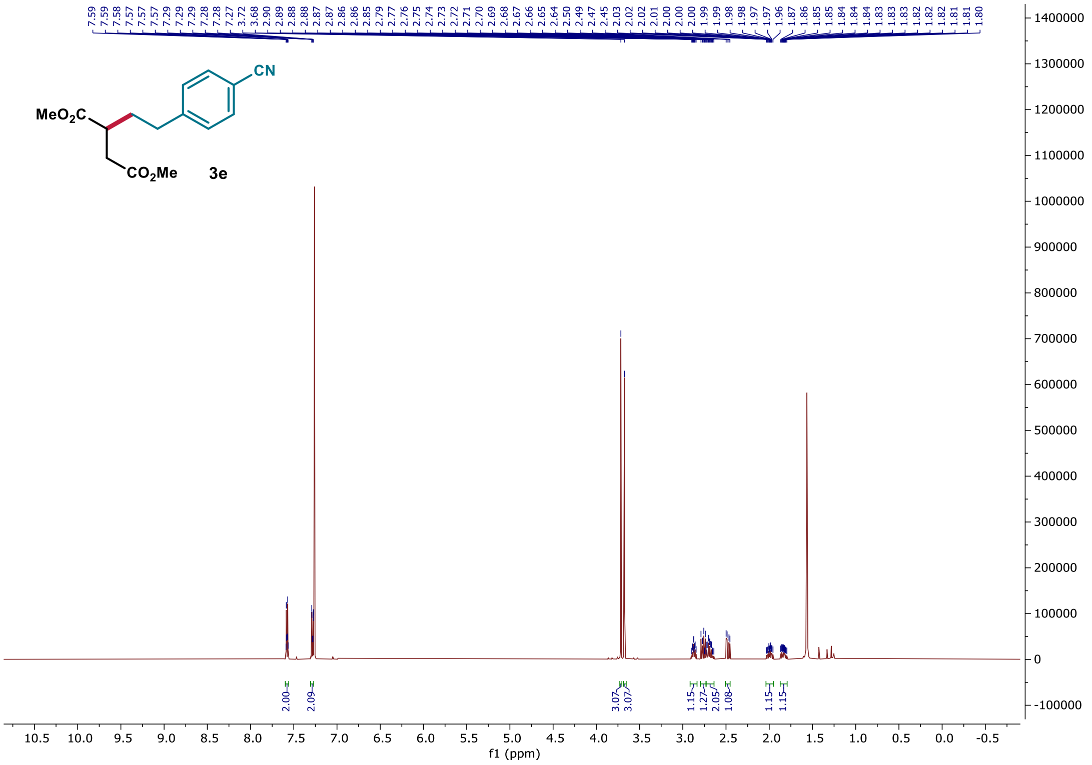

^{13}C NMR (126 MHz, $CDCl_3$) of **3e**

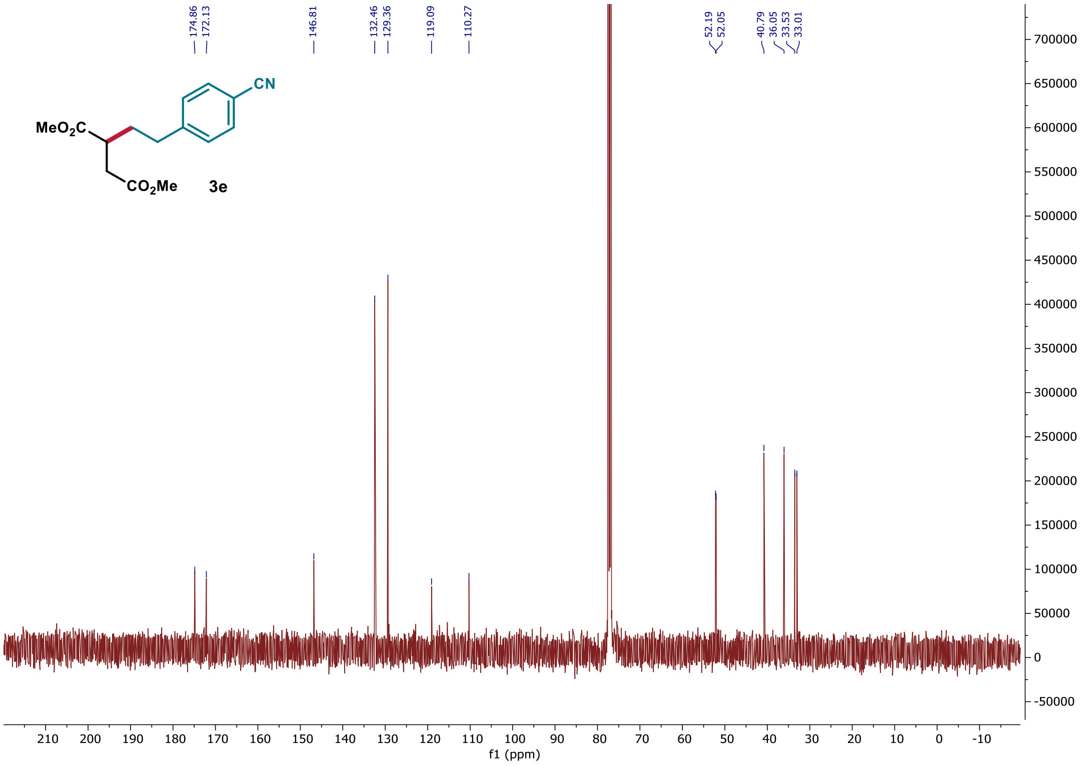

^{1}H NMR (500 MHz, $CDCl_3$) of **3f**

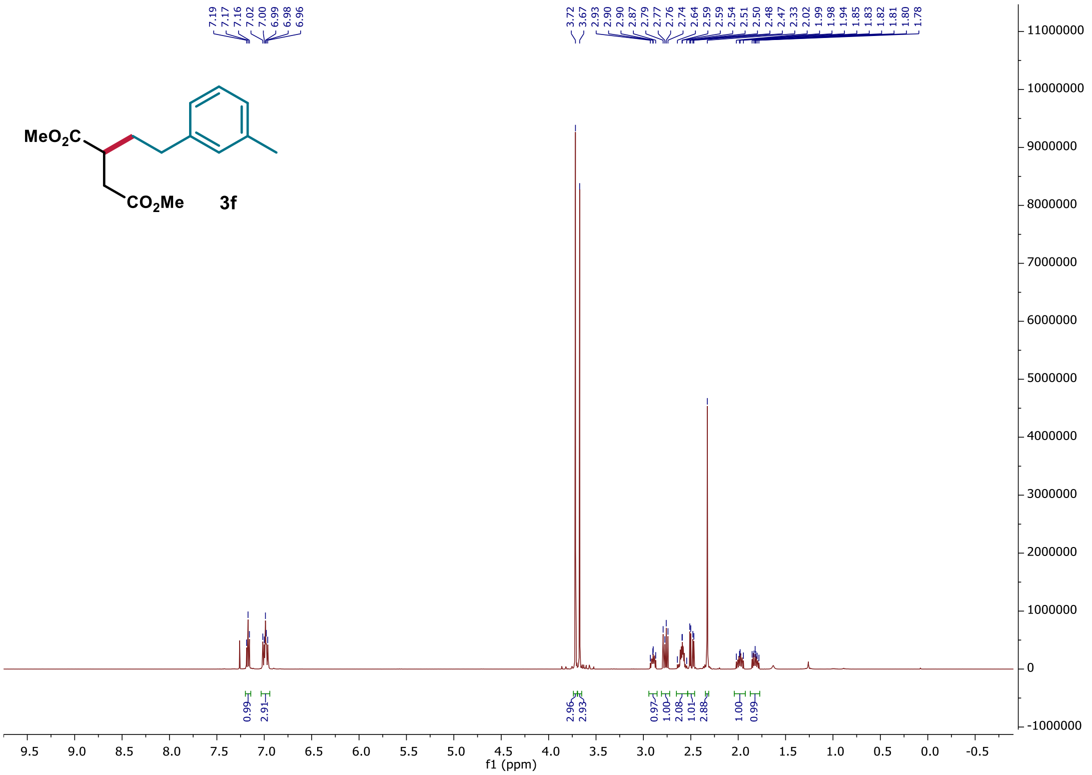

^{13}C NMR (126 MHz, $CDCl_3$) of **3f**

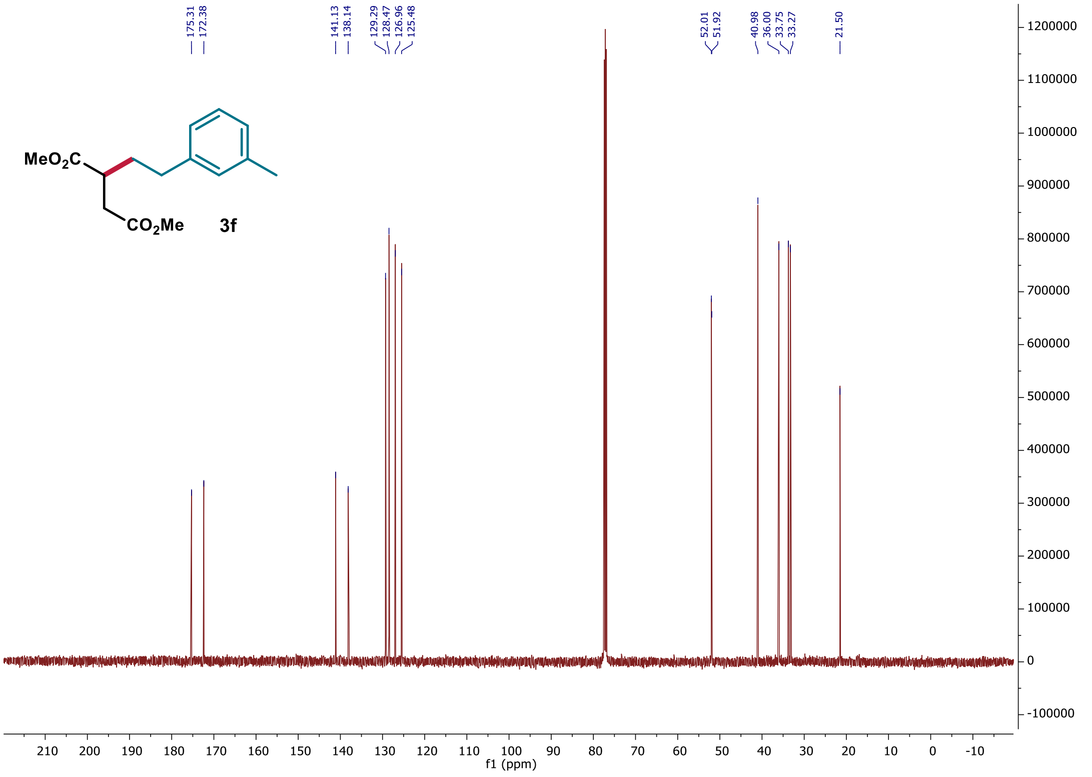

^{1}H NMR (500 MHz, $CDCl_3$) of **3g**

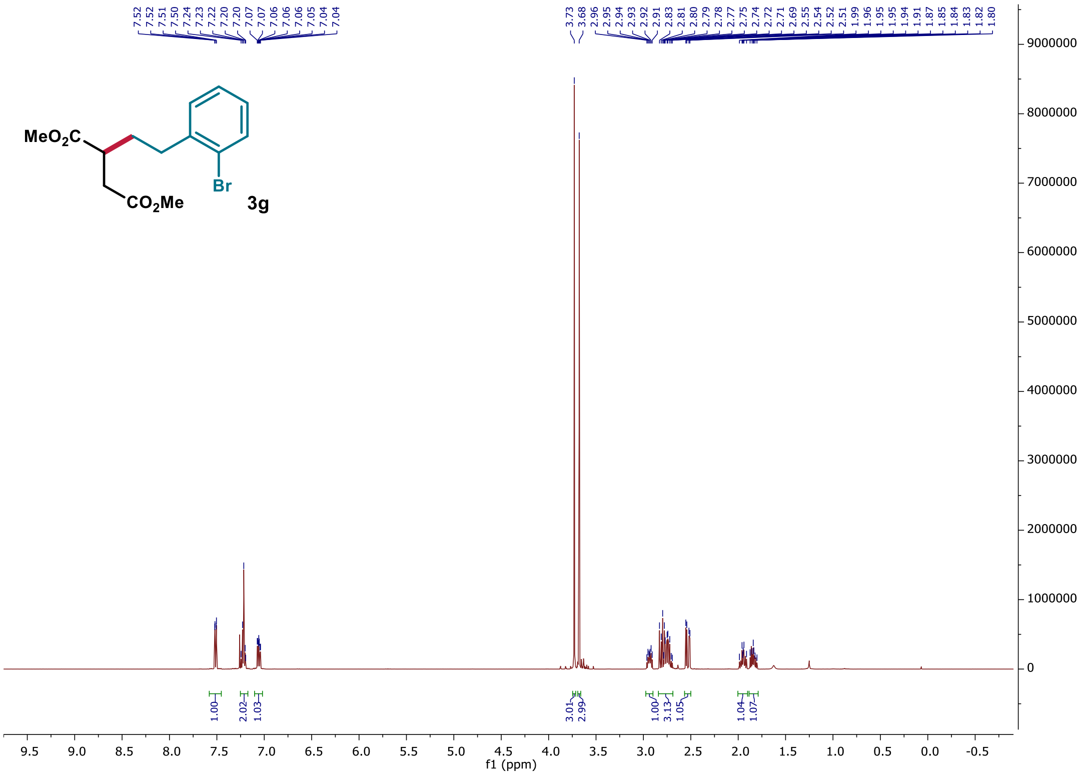

^{13}C NMR (126 MHz, $CDCl_3$) of **3g**

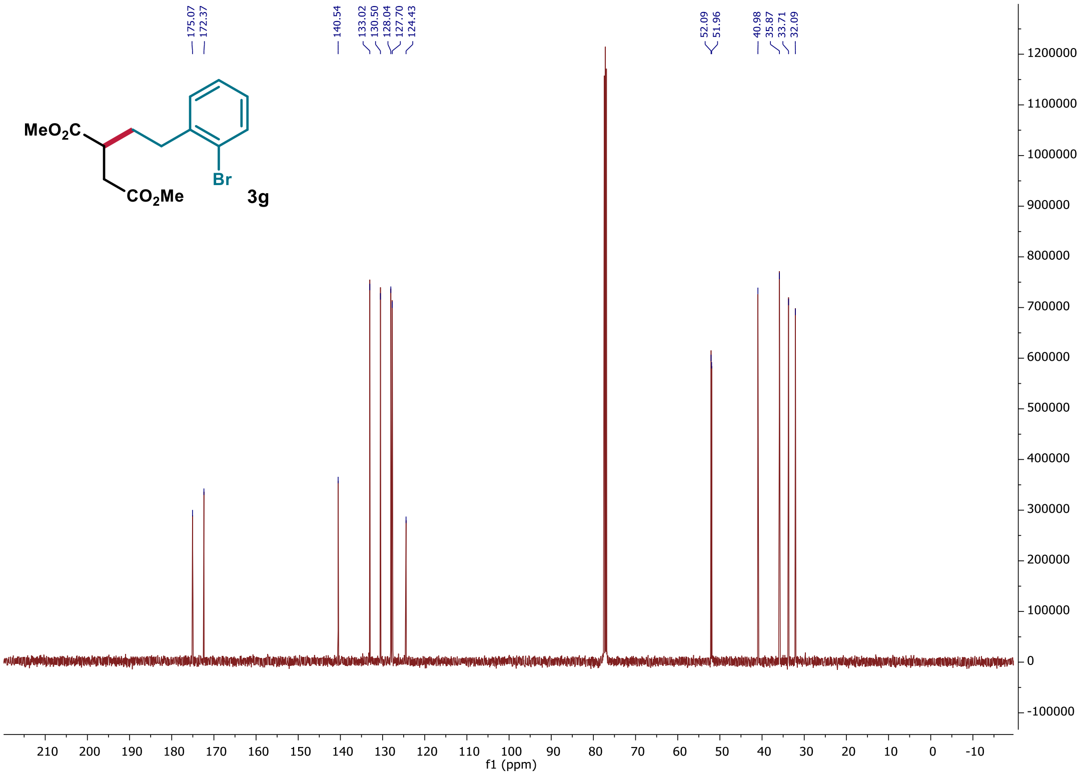

^{1}H NMR (500 MHz, $CDCl_3$) of **3h**

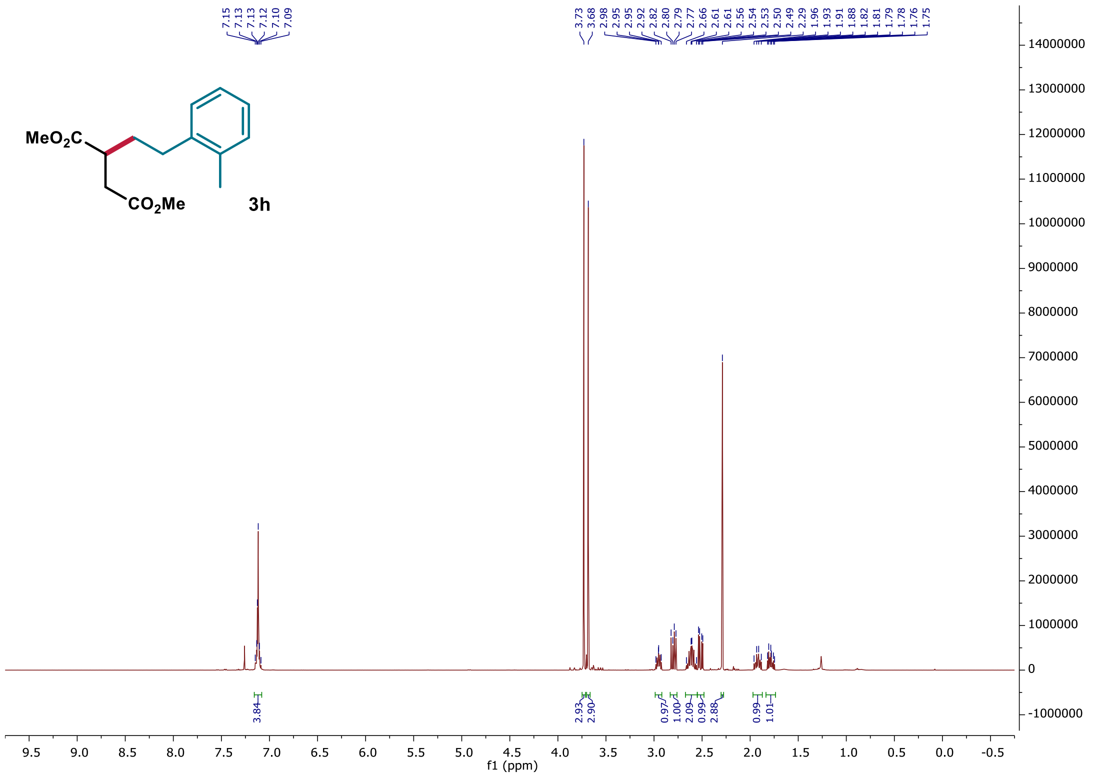

^{13}C NMR (126 MHz, $CDCl_3$) of **3h**

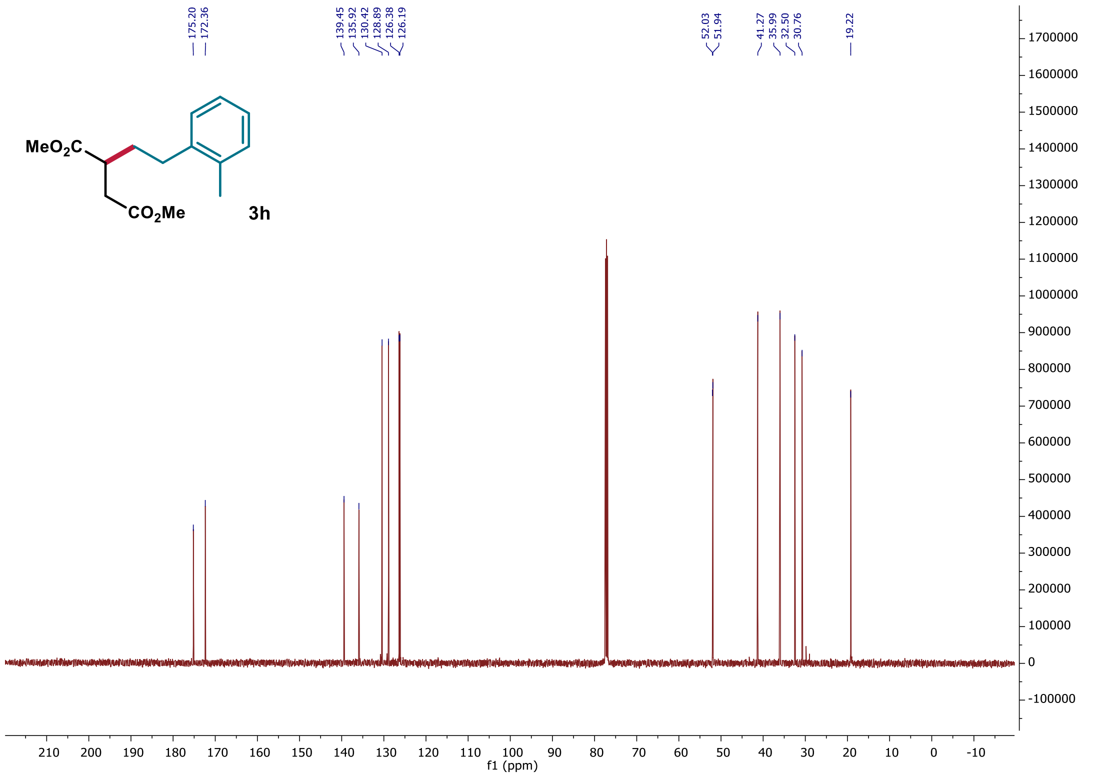

^{1}H NMR (500 MHz, $CDCl_3$) of **3i**

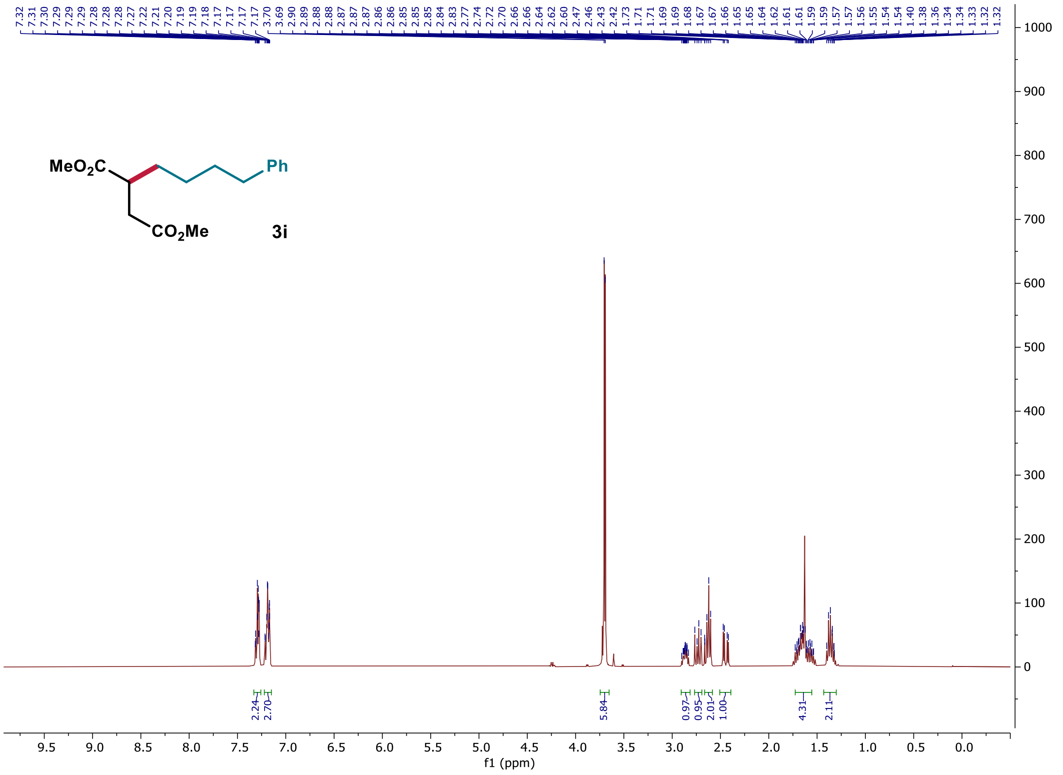

^{13}C NMR (101 MHz, $CDCl_3$) of **3i**

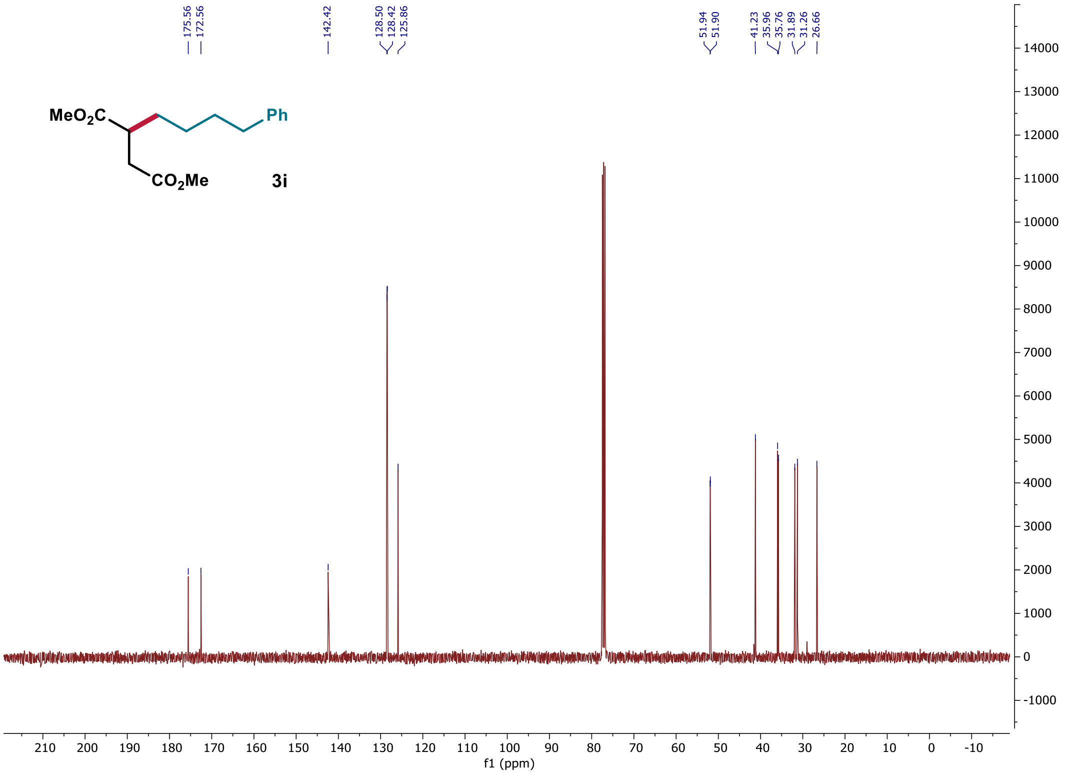

^{1}H NMR (400 MHz, $CDCl_3$) of **3j**

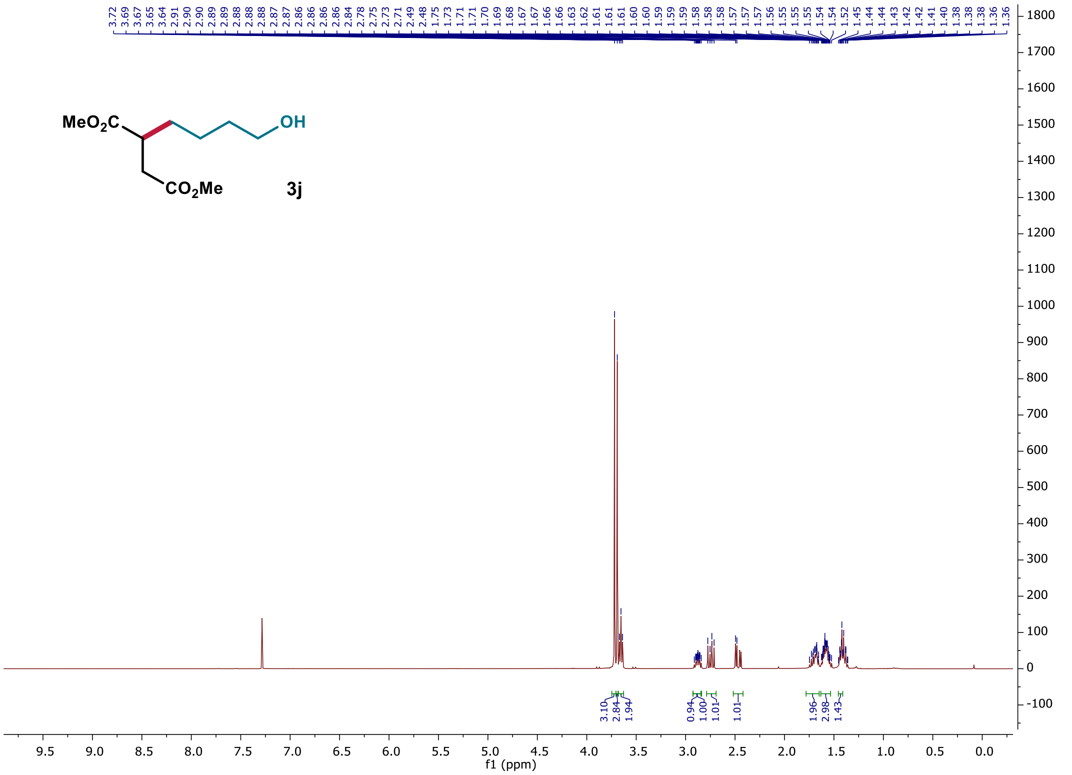

^{13}C NMR (101 MHz, $CDCl_3$) of **3j**

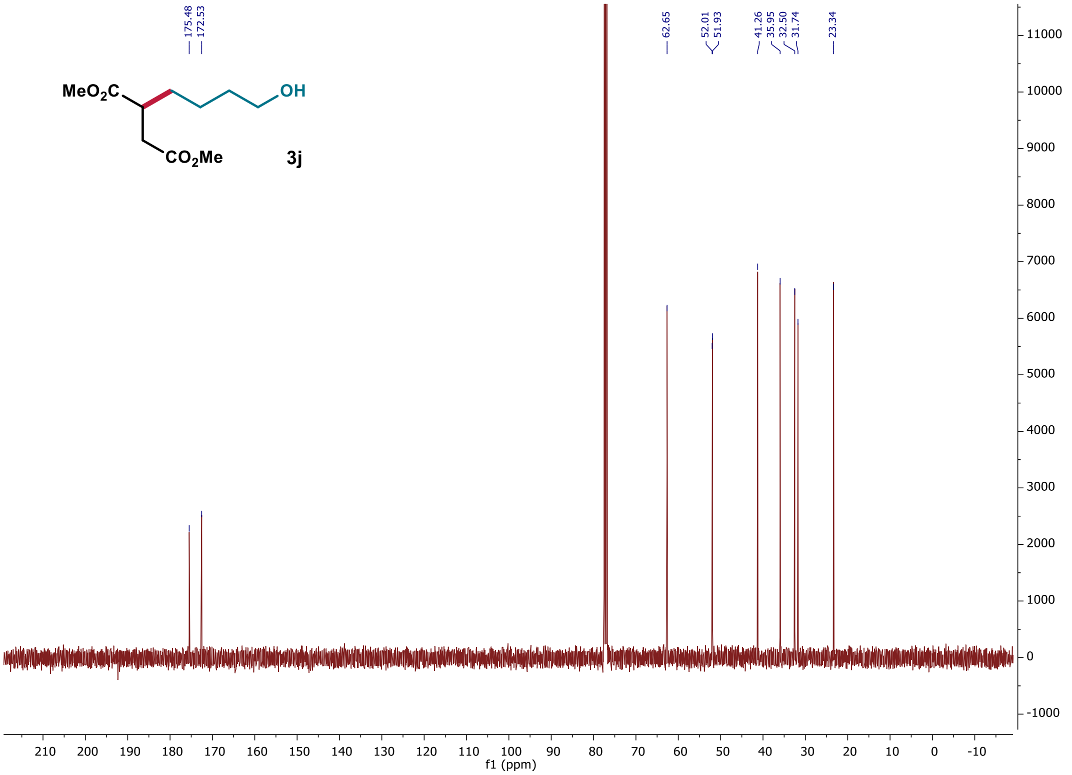

^{1}H NMR (500 MHz, $CDCl_3$) of **3k**

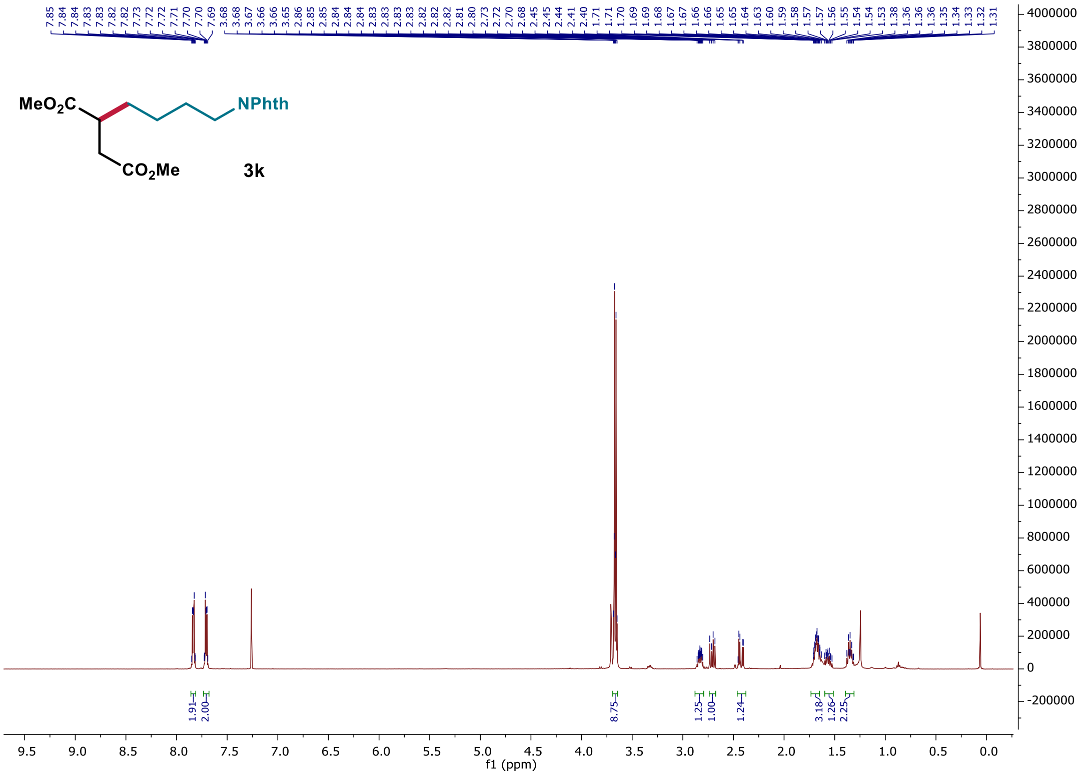

^{13}C NMR (101 MHz, $CDCl_3$) of **3k**

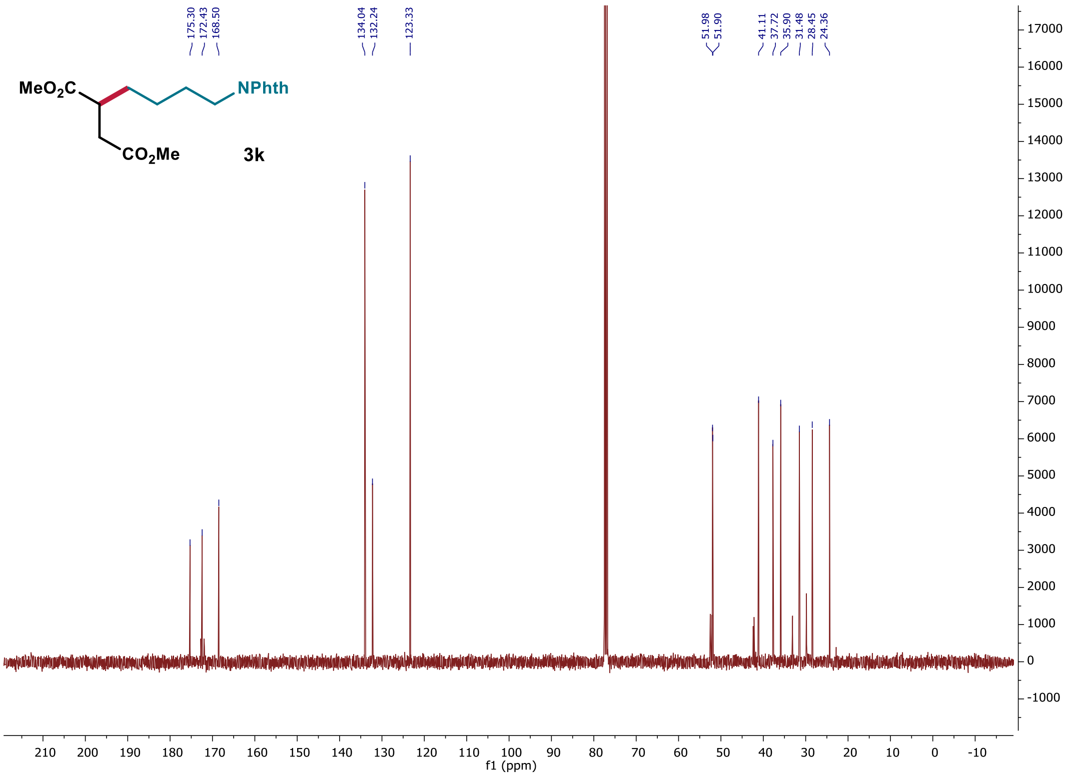

^{1}H NMR (500 MHz, $CDCl_3$) of **3l**

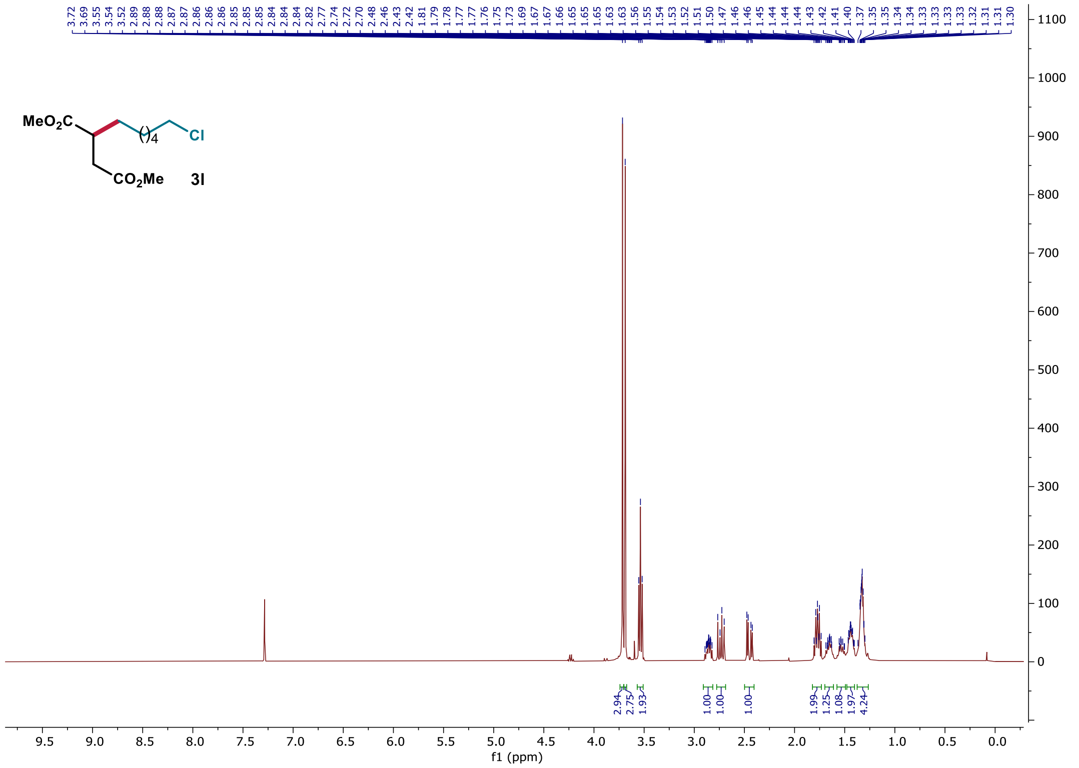

^{13}C NMR (101 MHz, $CDCl_3$) of **3l**

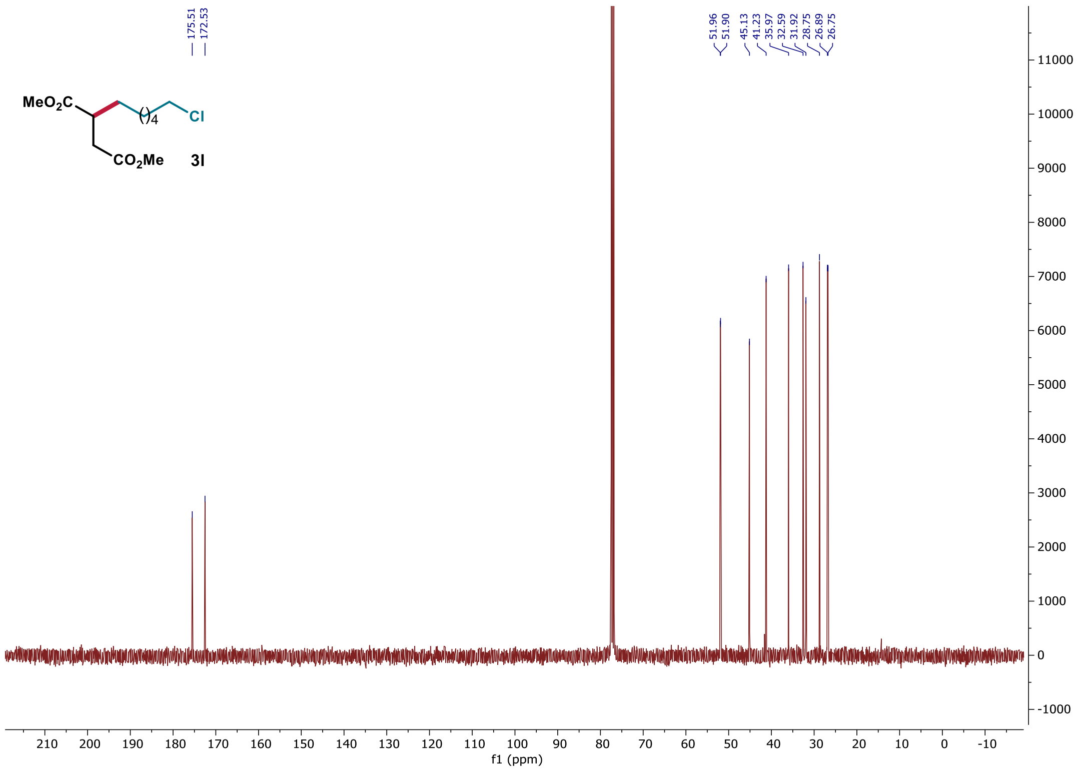

^{1}H NMR (500 MHz, $CDCl_3$) of **3m**

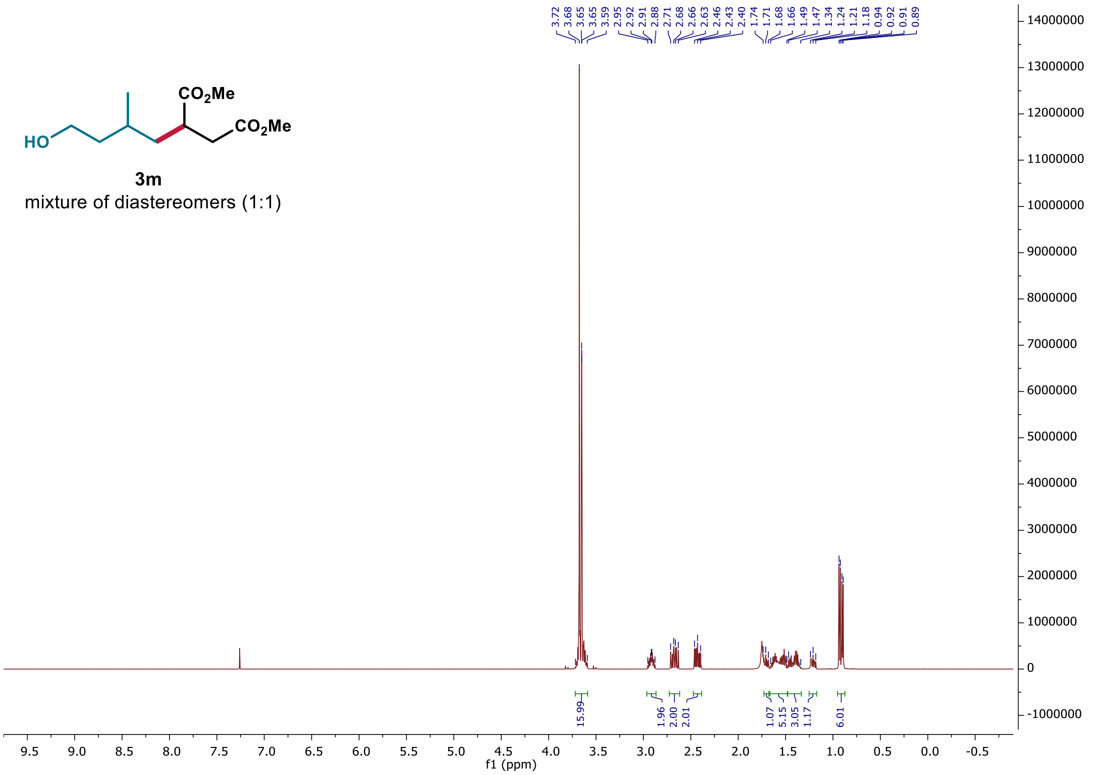

^{13}C NMR (126 MHz, $CDCl_3$) of **3m**

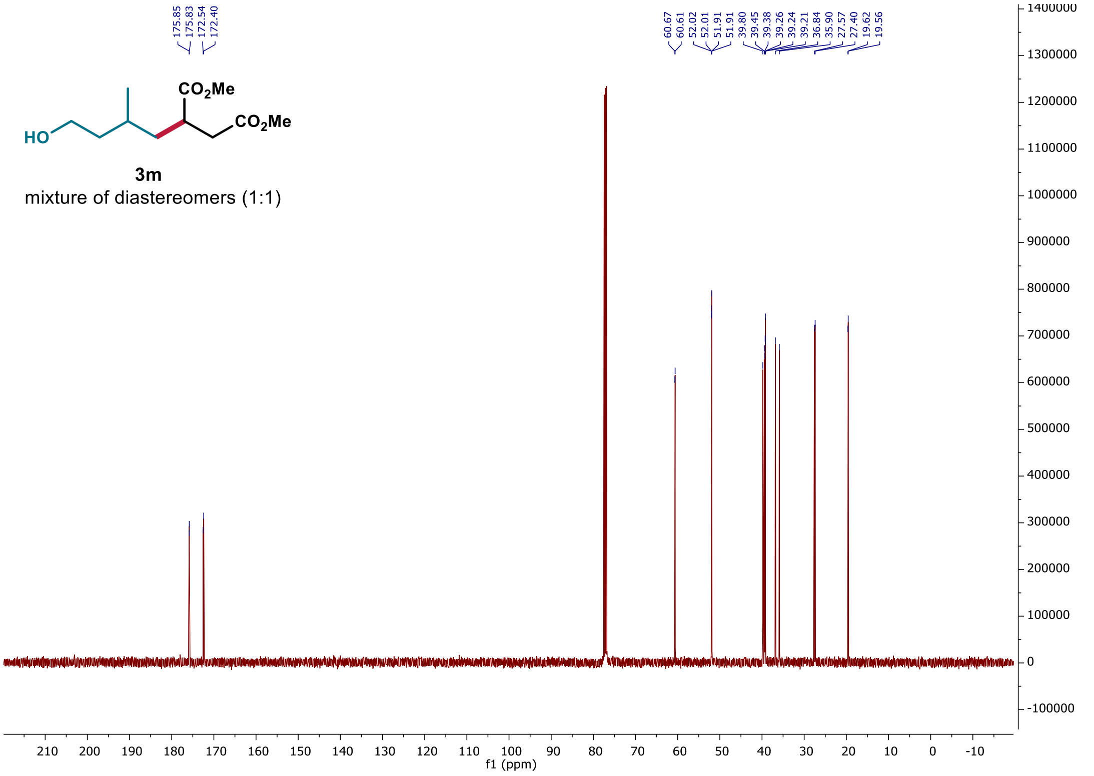

<u>^{1}H NMR</u> (500 MHz, $CDCl_3$) of **3n**

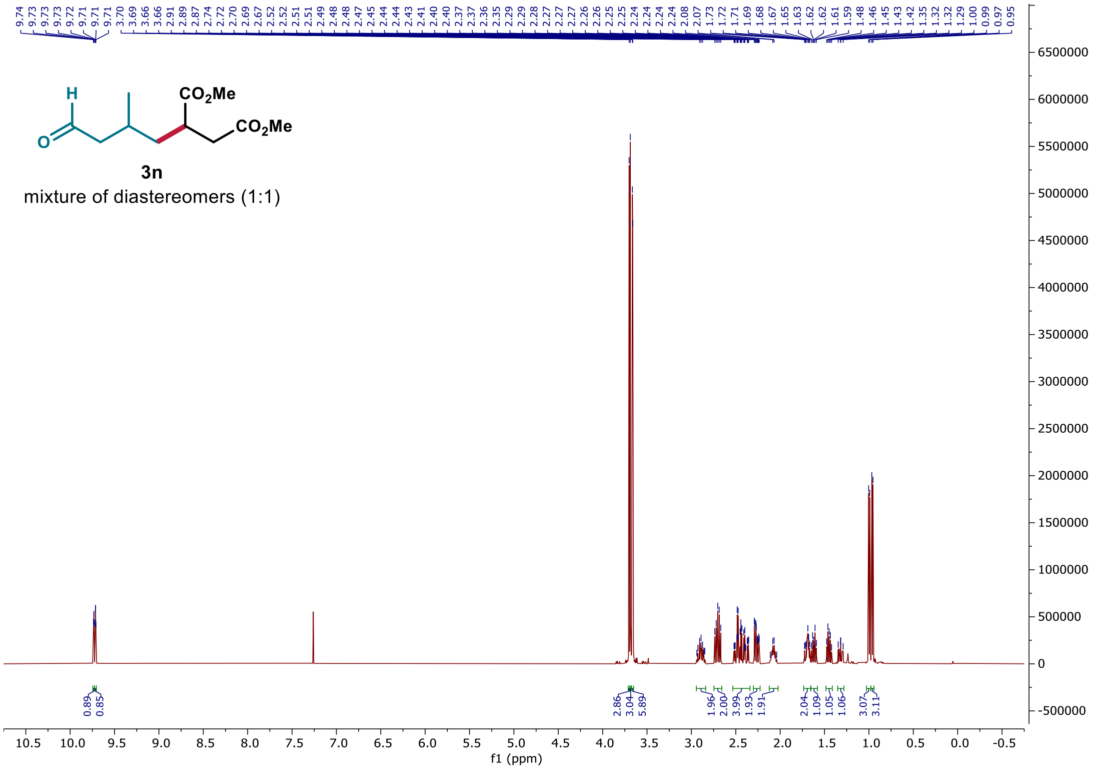

<u>^{13}C NMR</u> (126 MHz, $CDCl_3$) of **3n**

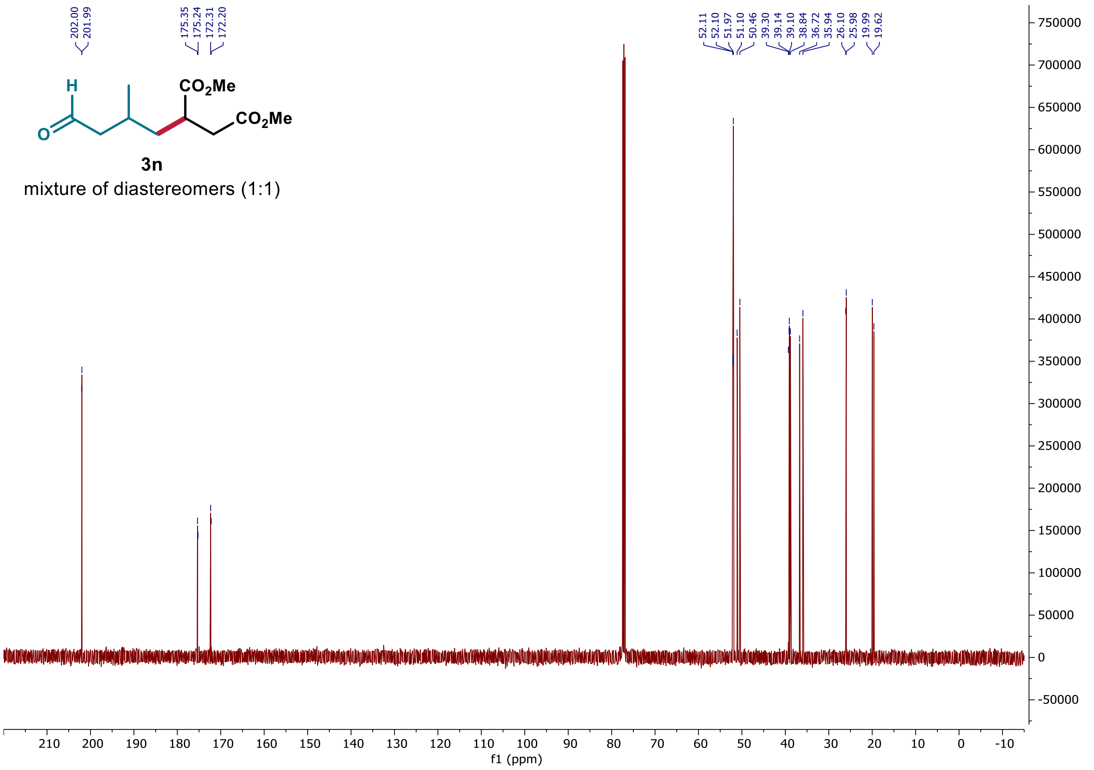

^{1}H NMR (400 MHz, $CDCl_3$) of **3o**

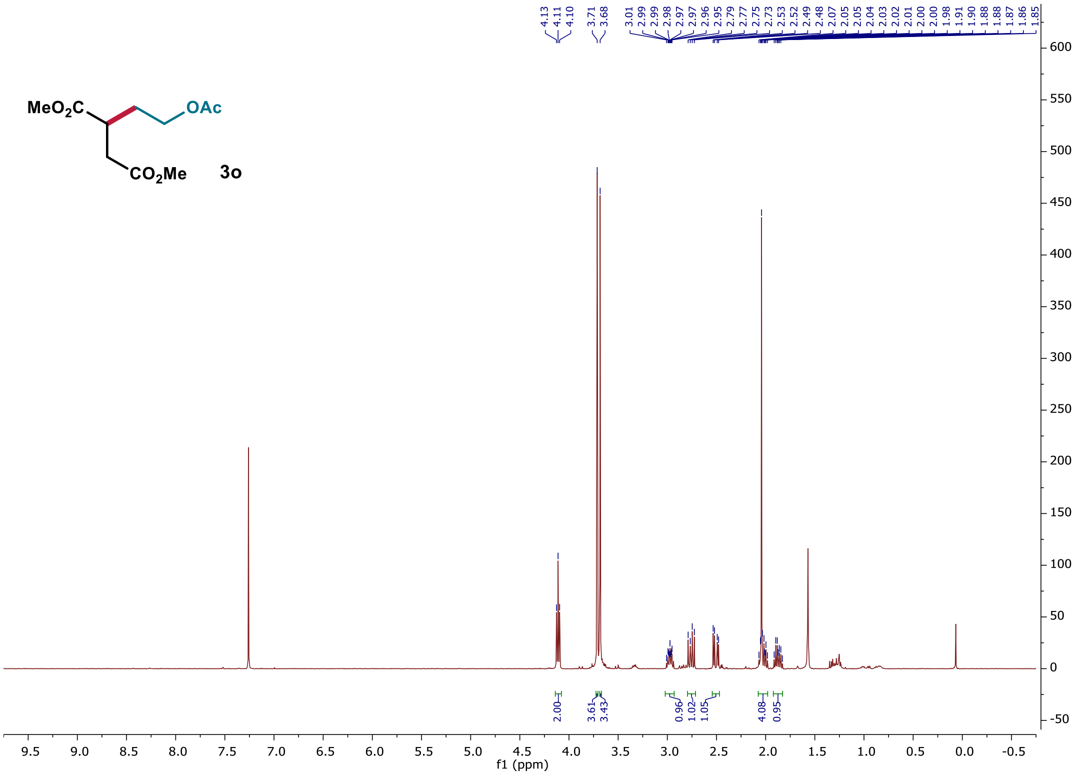

^{13}C NMR (101 MHz, $CDCl_3$) of **3o**

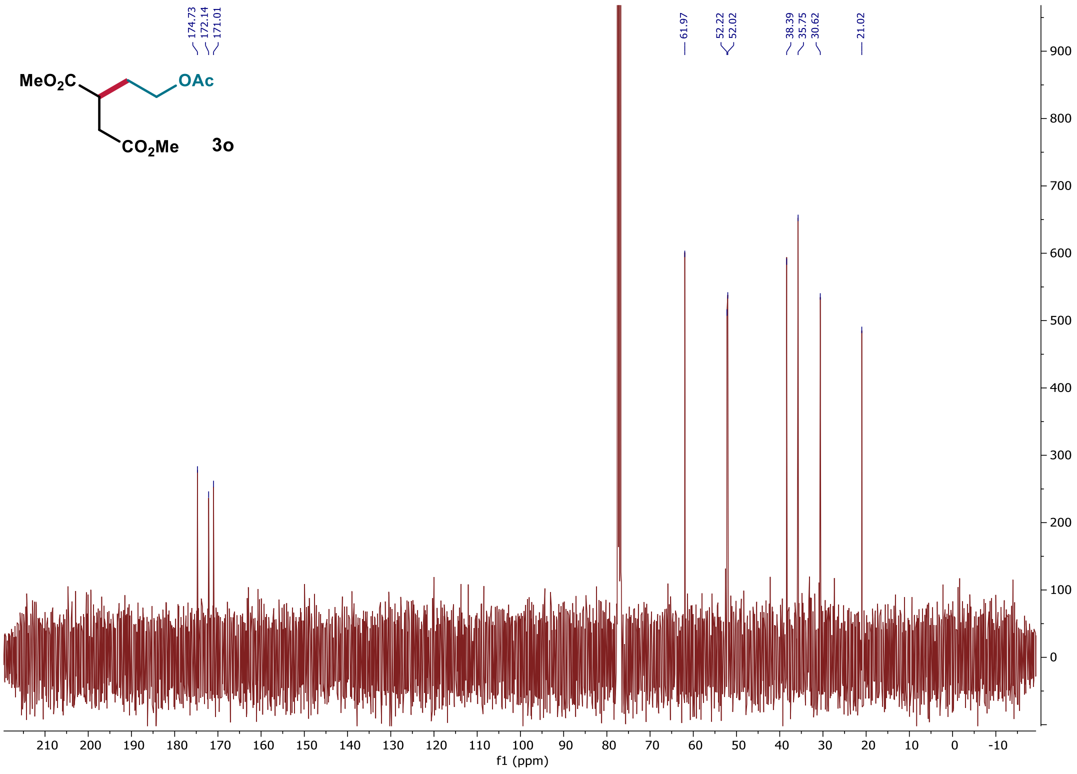

^{1}H NMR (500 MHz, $CDCl_3$) of **3p**

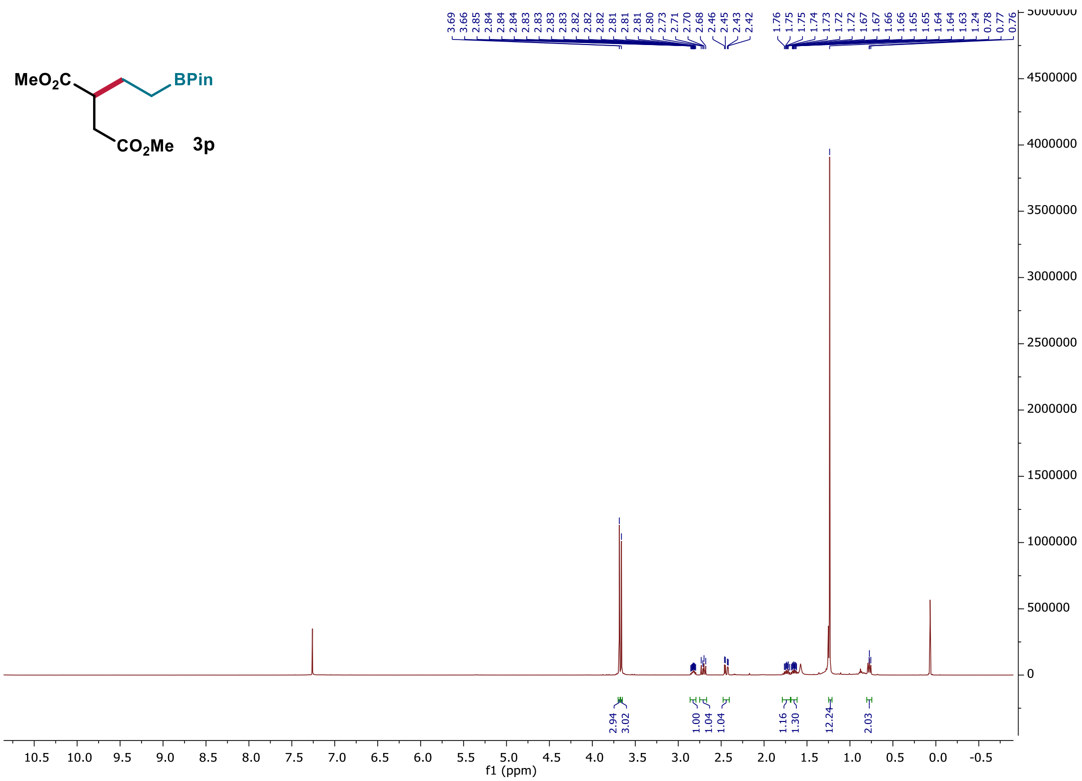

^{13}C NMR (126 MHz, $CDCl_3$) of **3p**

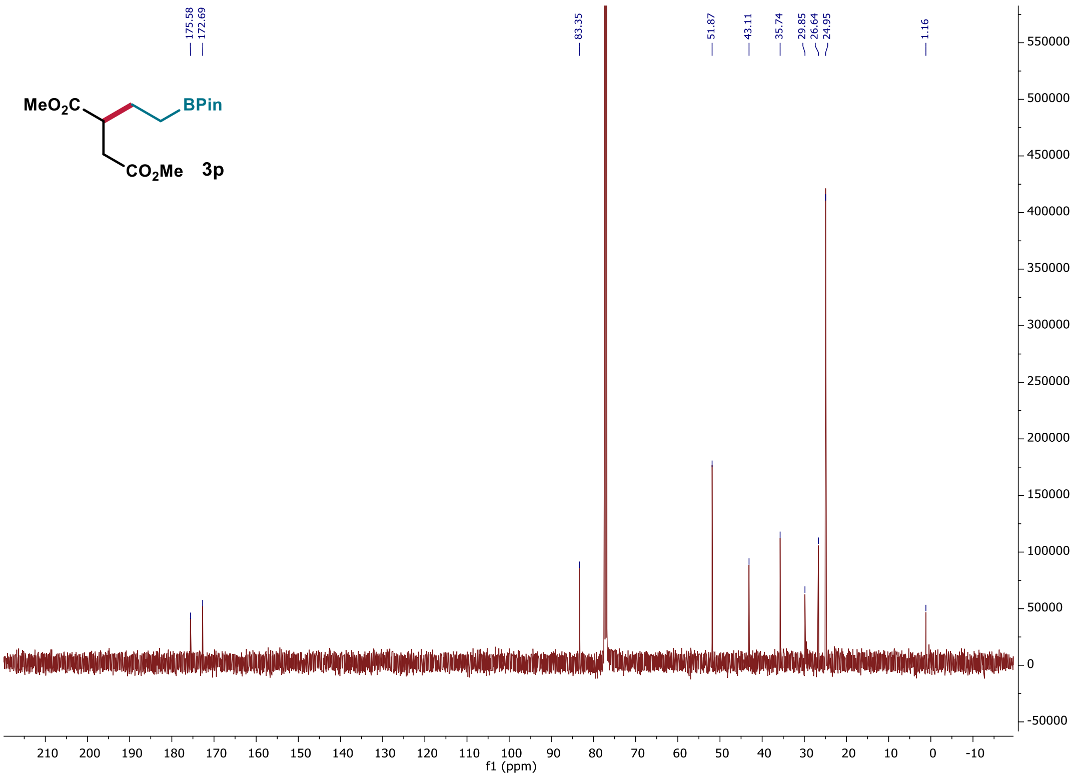

^{1}H NMR (400 MHz, $CDCl_3$) of **3q**

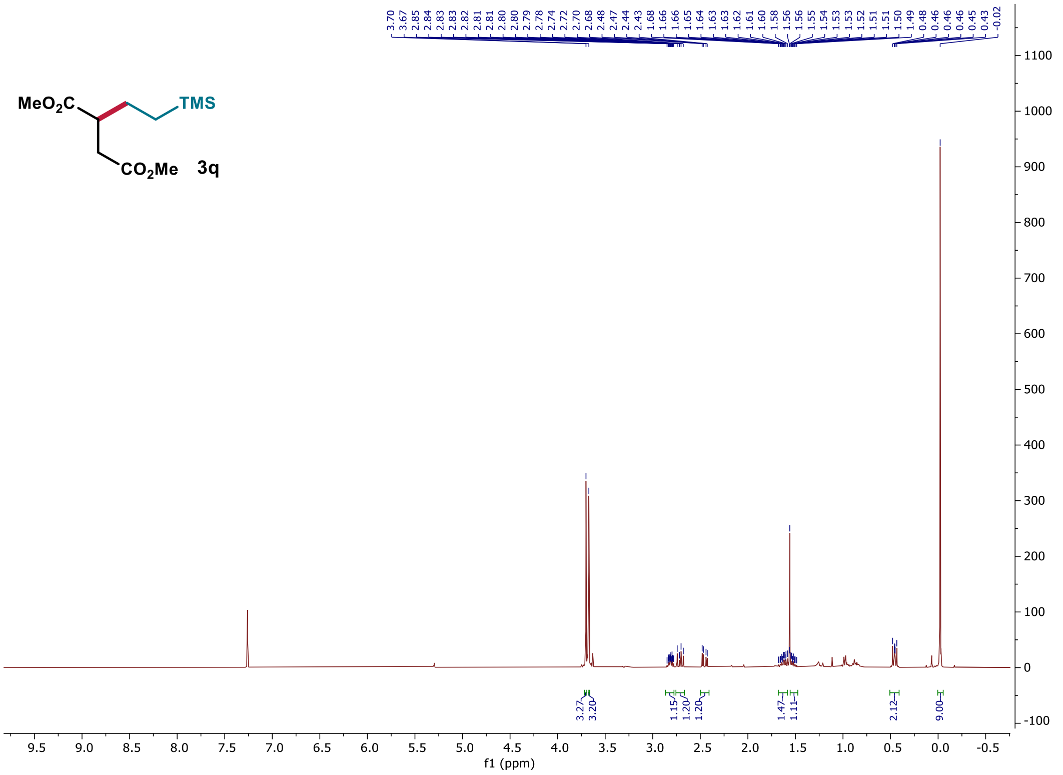

^{13}C NMR (126 MHz, $CDCl_3$) of **3q**

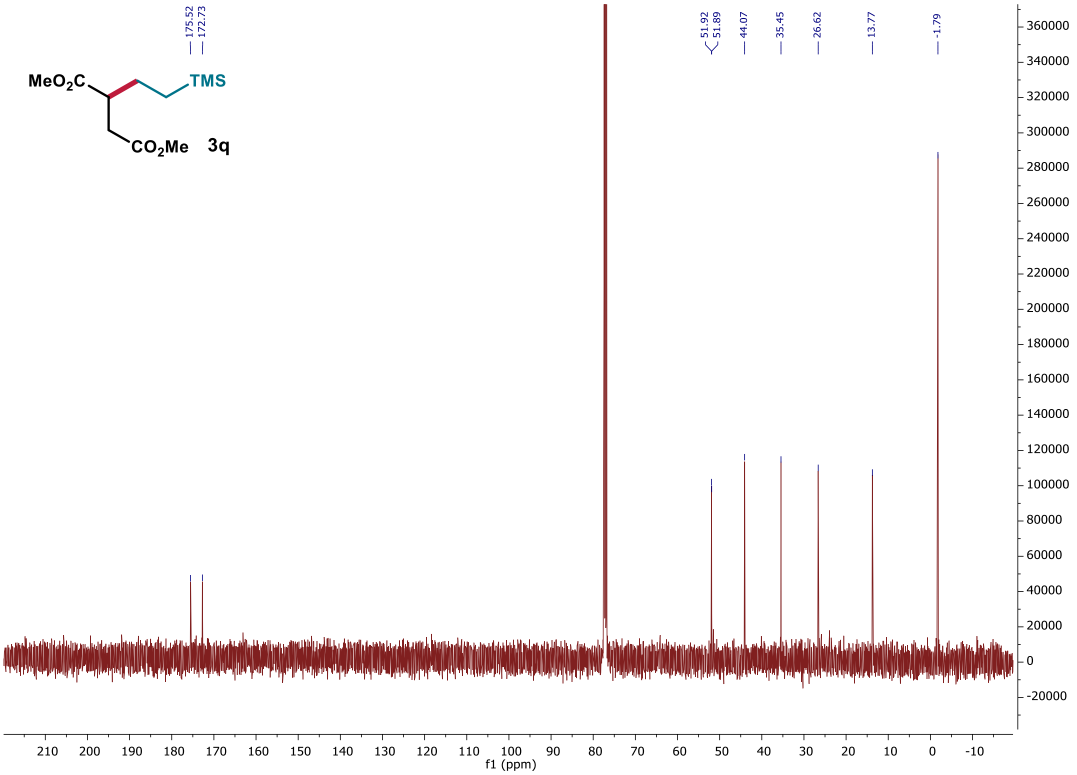

^{1}H NMR (500 MHz, $CDCl_3$) of **3r**

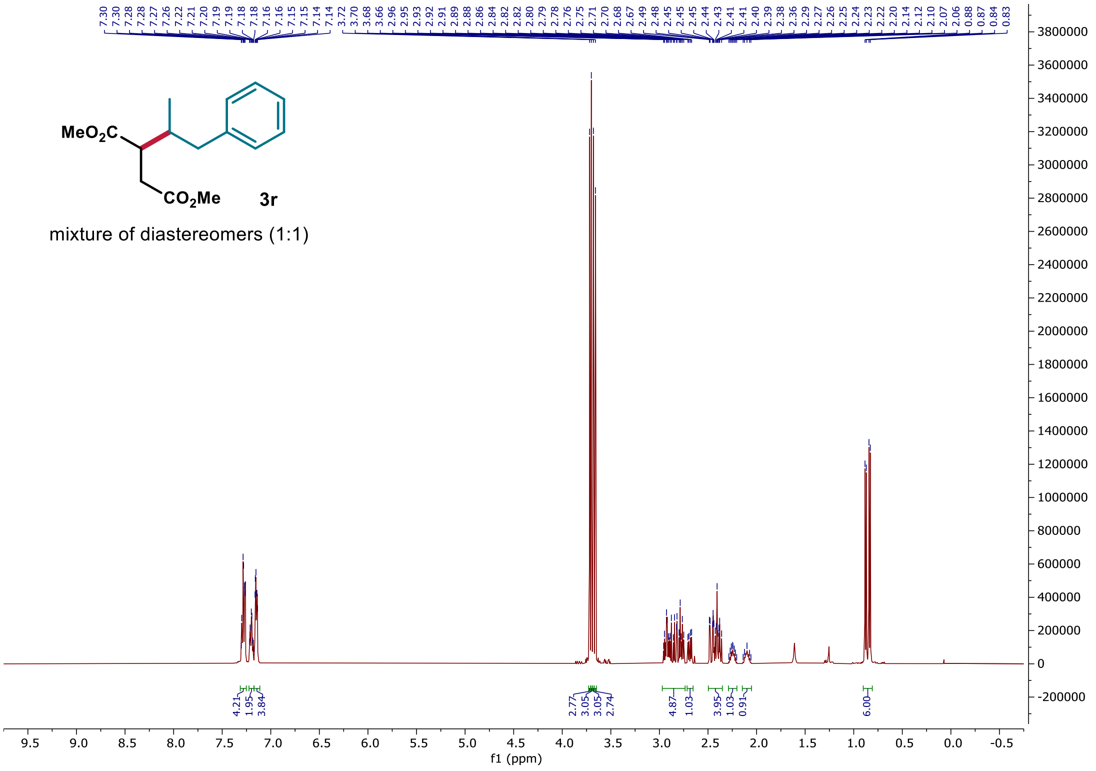

^{13}C NMR (126 MHz, $CDCl_3$) of **3r**

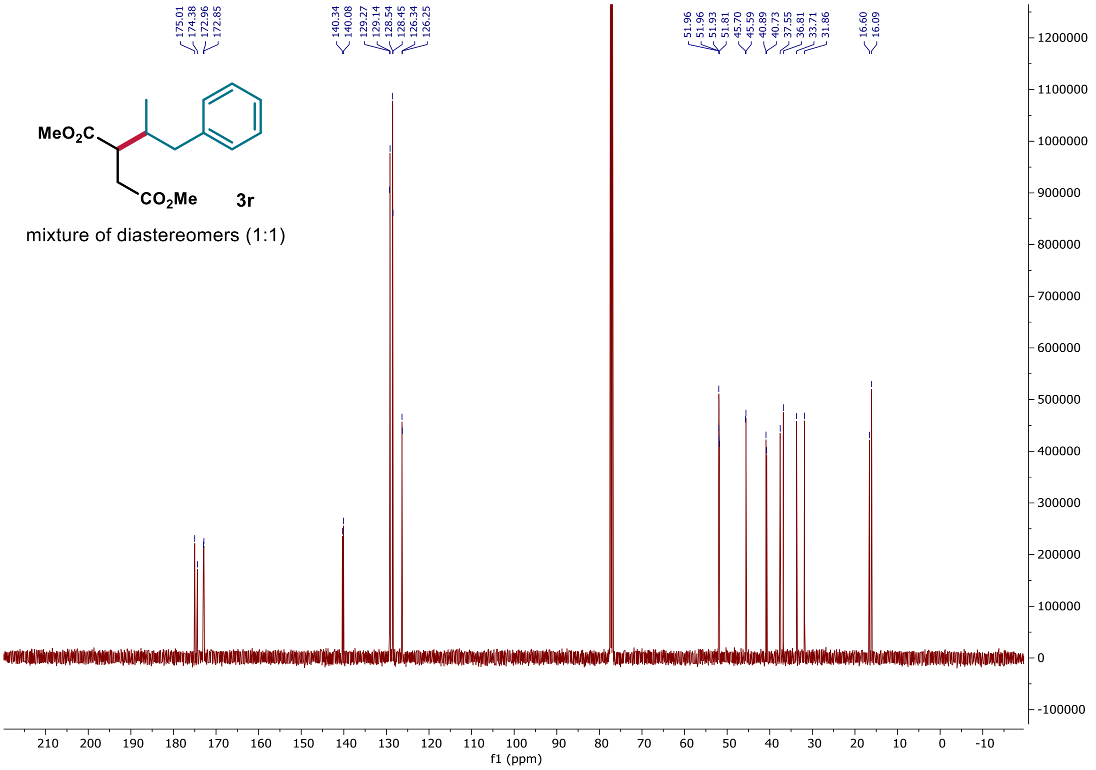

^{1}H NMR (500 MHz, $CDCl_3$) of **3s**

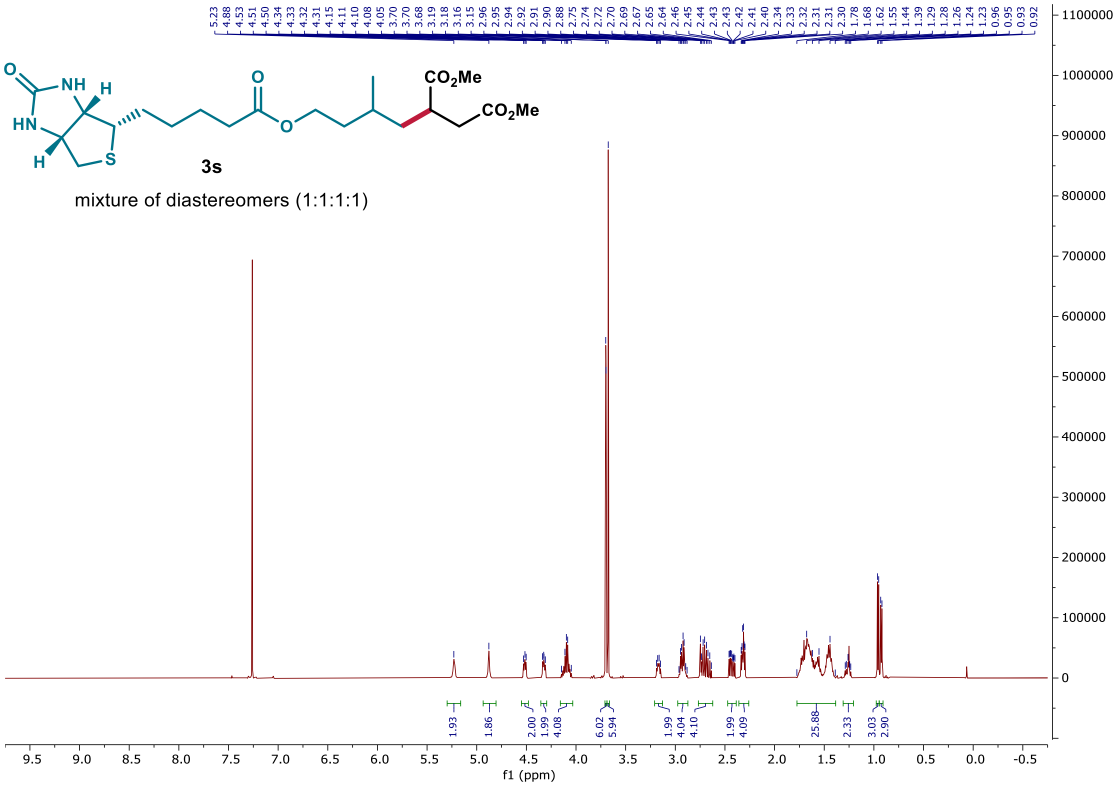

^{13}C NMR (126 MHz, $CDCl_3$) of **3s**

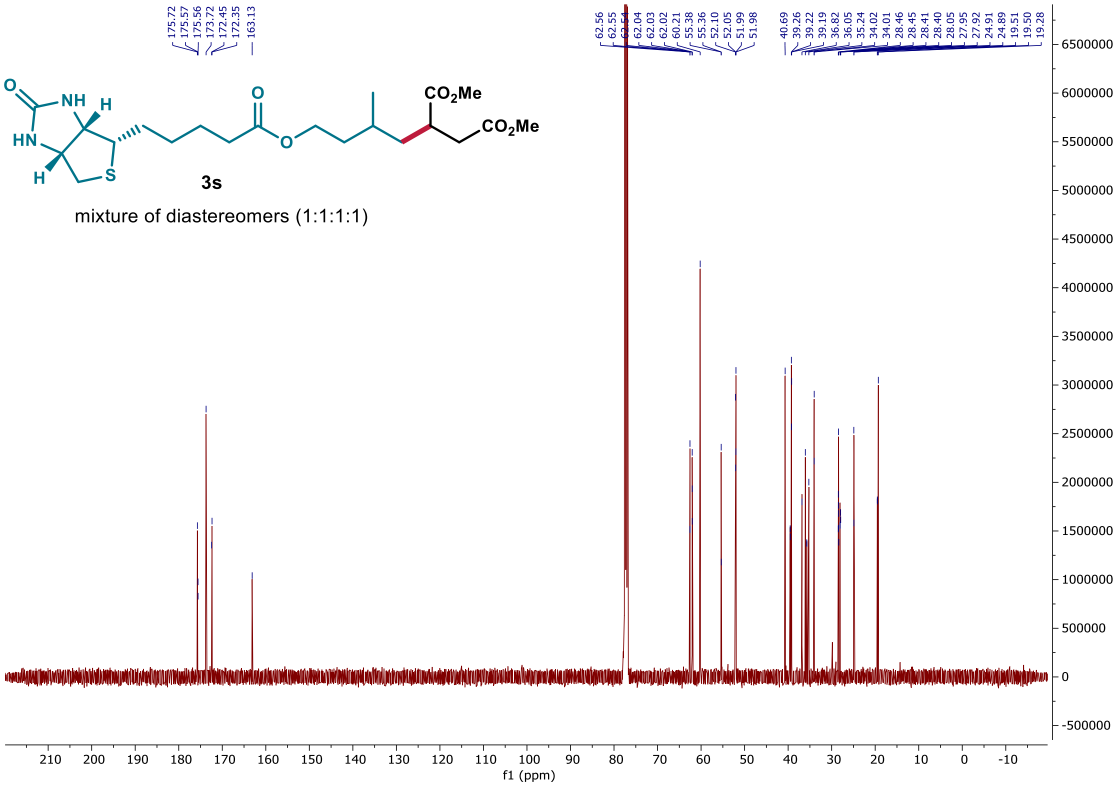

^{1}H NMR (400 MHz, $CDCl_3$) of **3t**

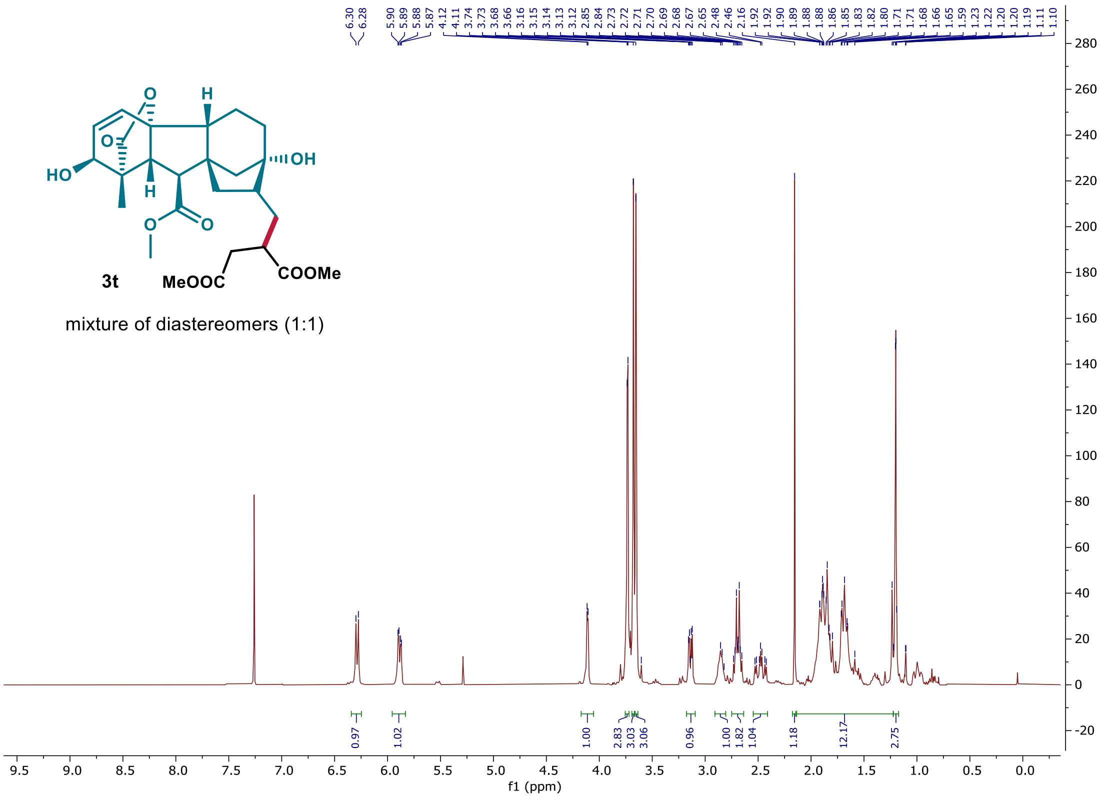

^{1}H NMR (500 MHz, $CDCl_3$) of **3t**

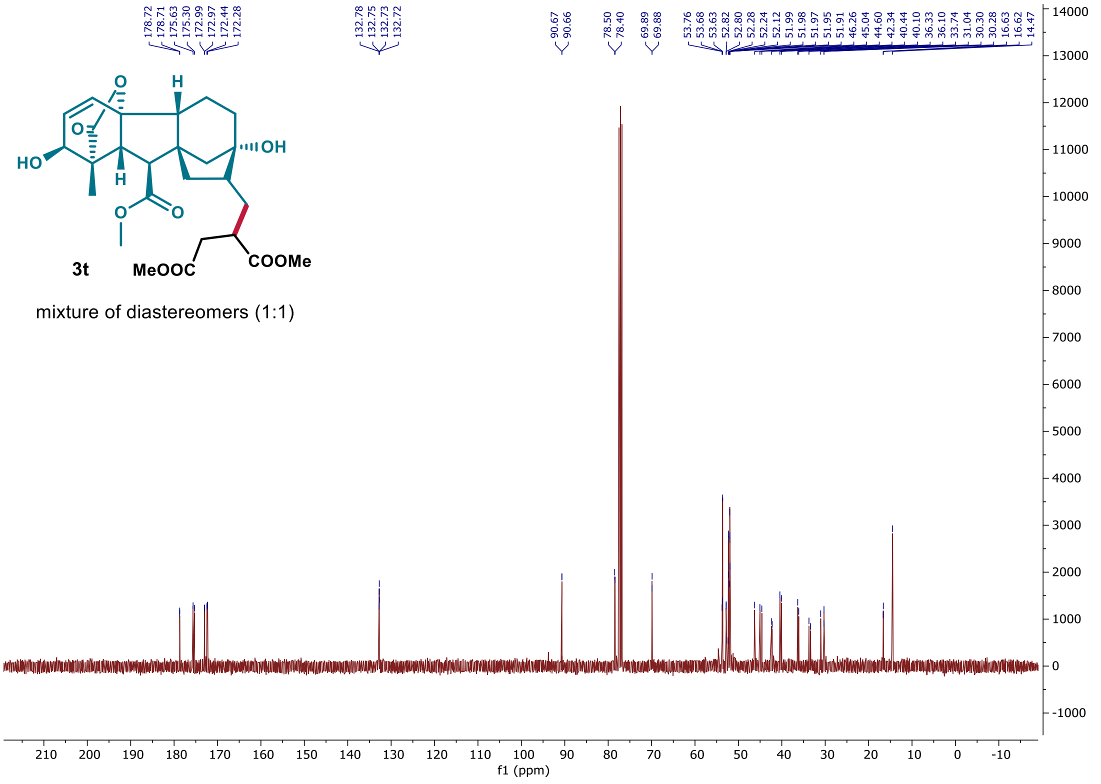

^{1}H NMR (500 MHz, $CDCl_3$) of **3u**

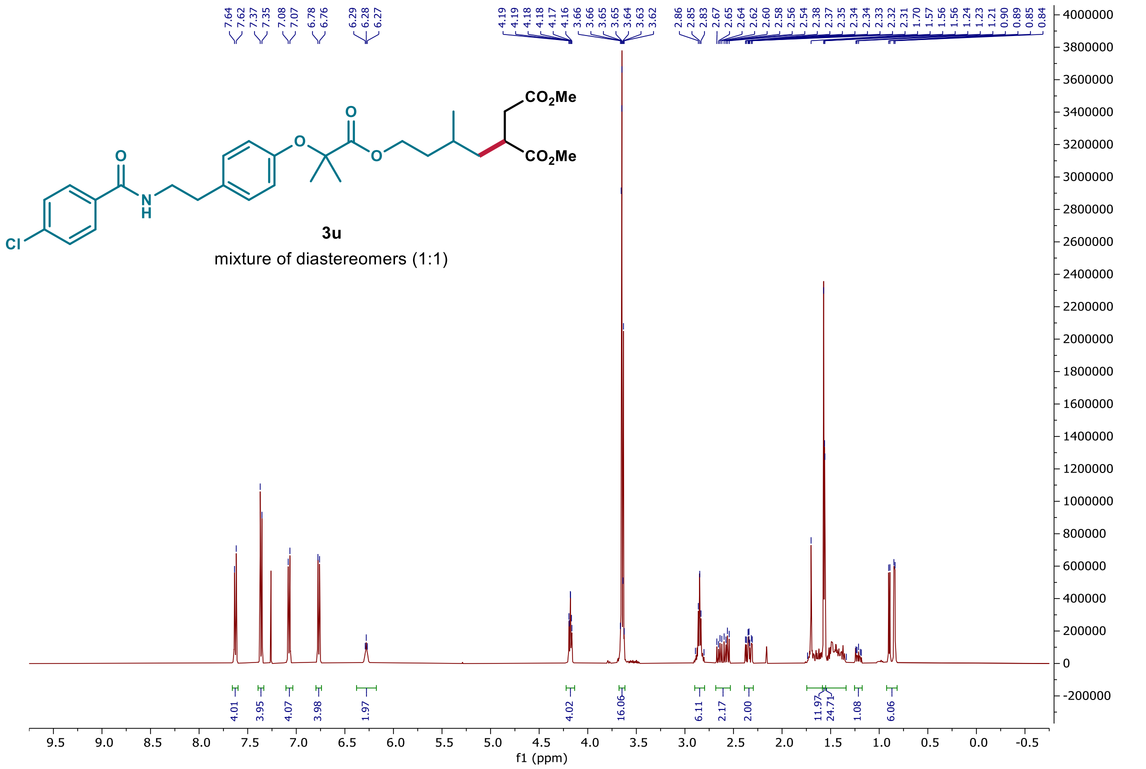

^{13}C NMR (126 MHz, $CDCl_3$) of **3u**

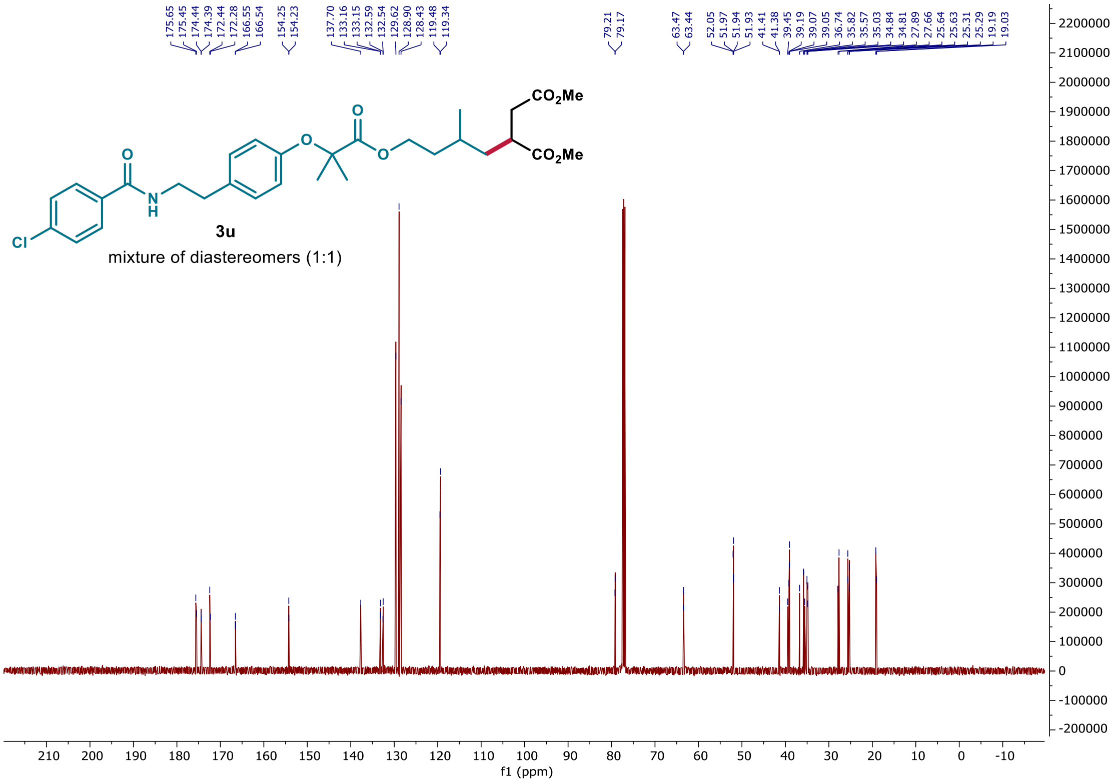

^{1}H NMR (500 MHz, $CDCl_3$) of **3v**

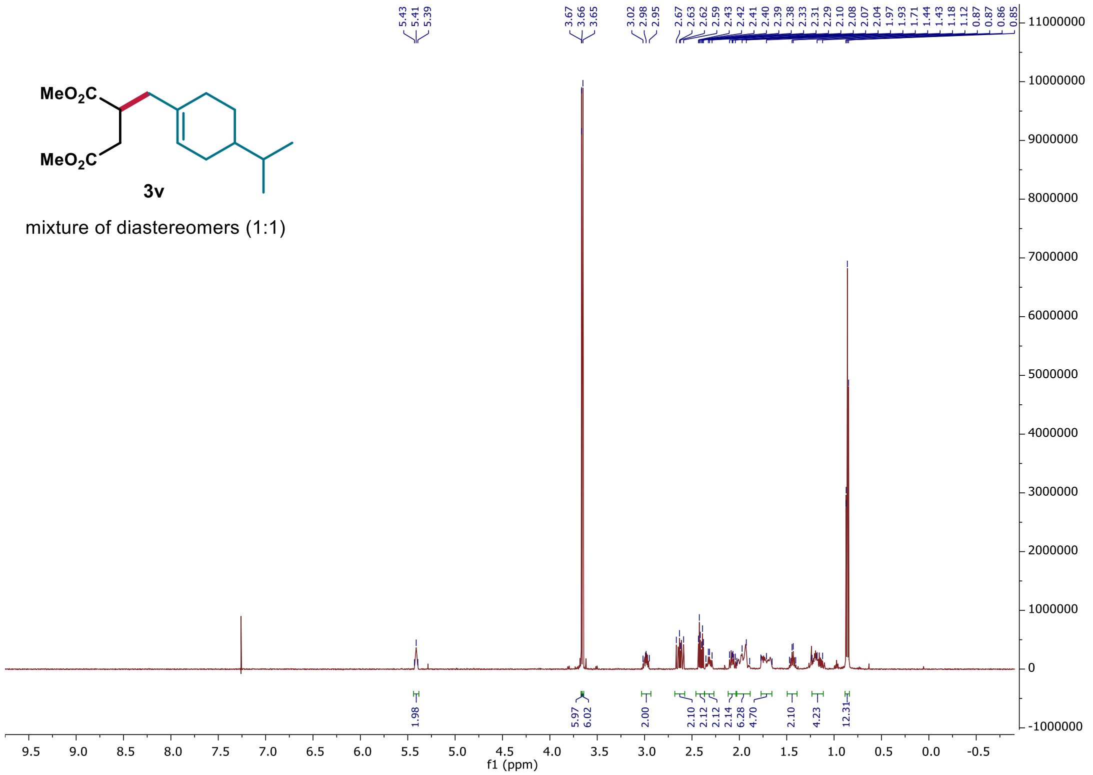

^{13}C NMR (126 MHz, $CDCl_3$) of **3v**

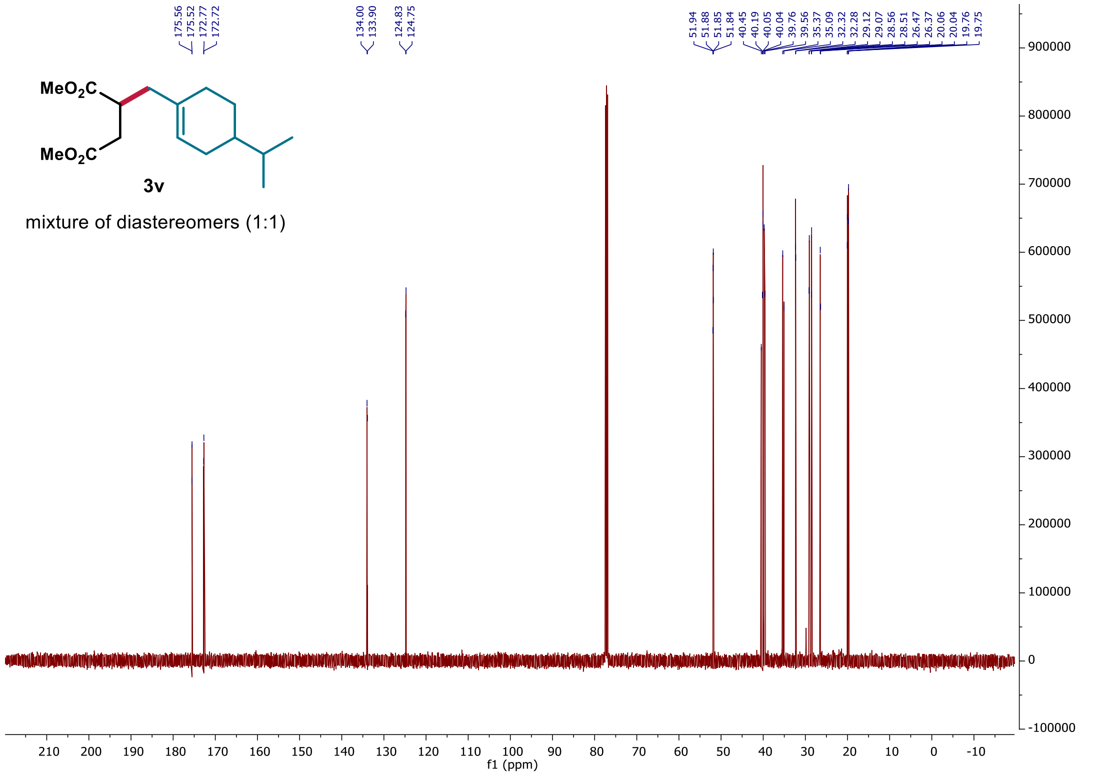

^{1}H NMR (500 MHz, $CDCl_3$) of **3w**

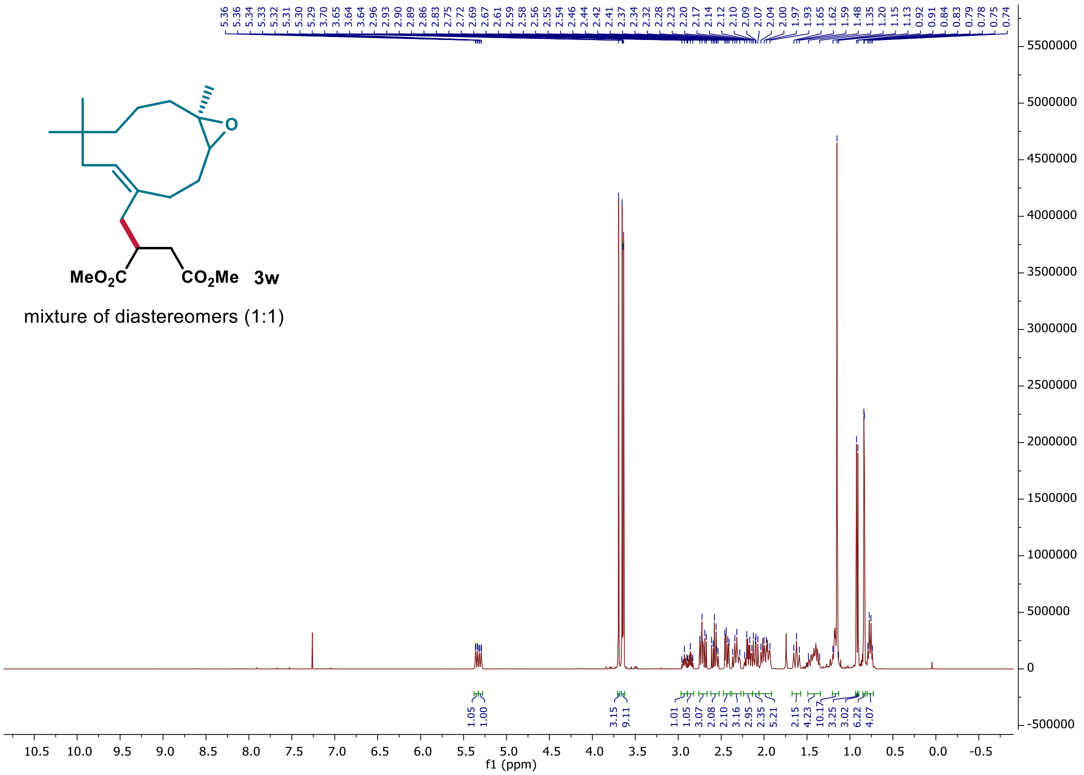

^{13}C NMR (126 MHz, $CDCl_3$) of **3w**

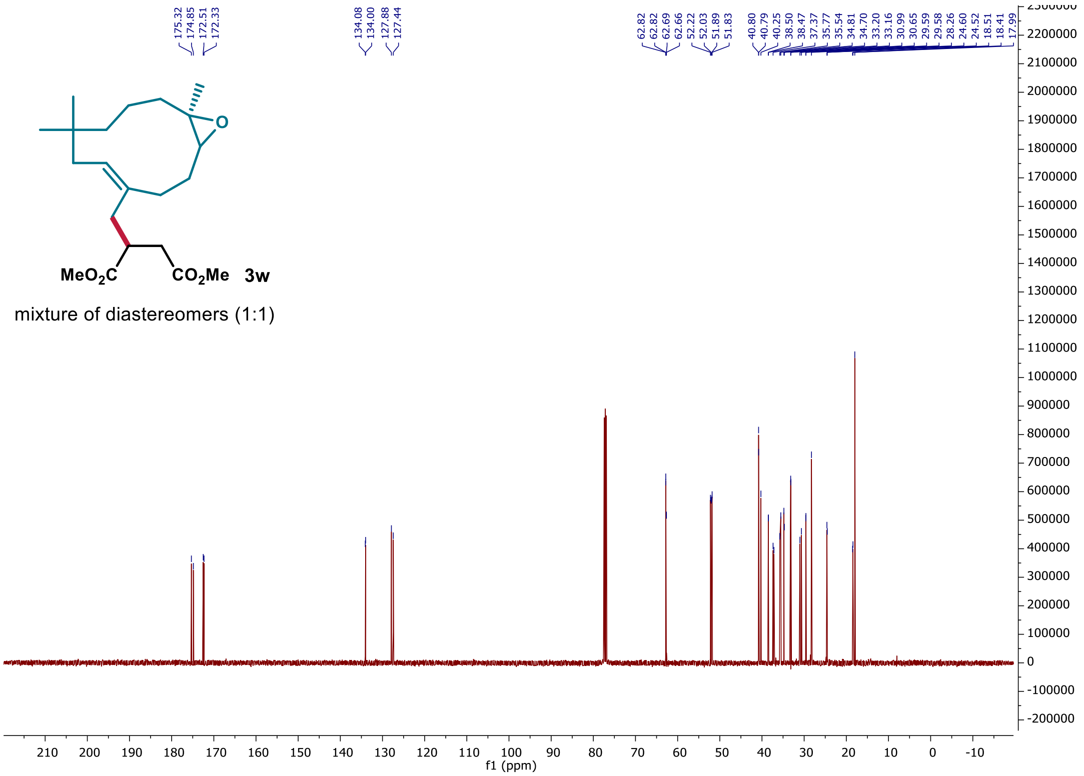

^{1}H NMR (500 MHz, $CDCl_3$) of **6a**

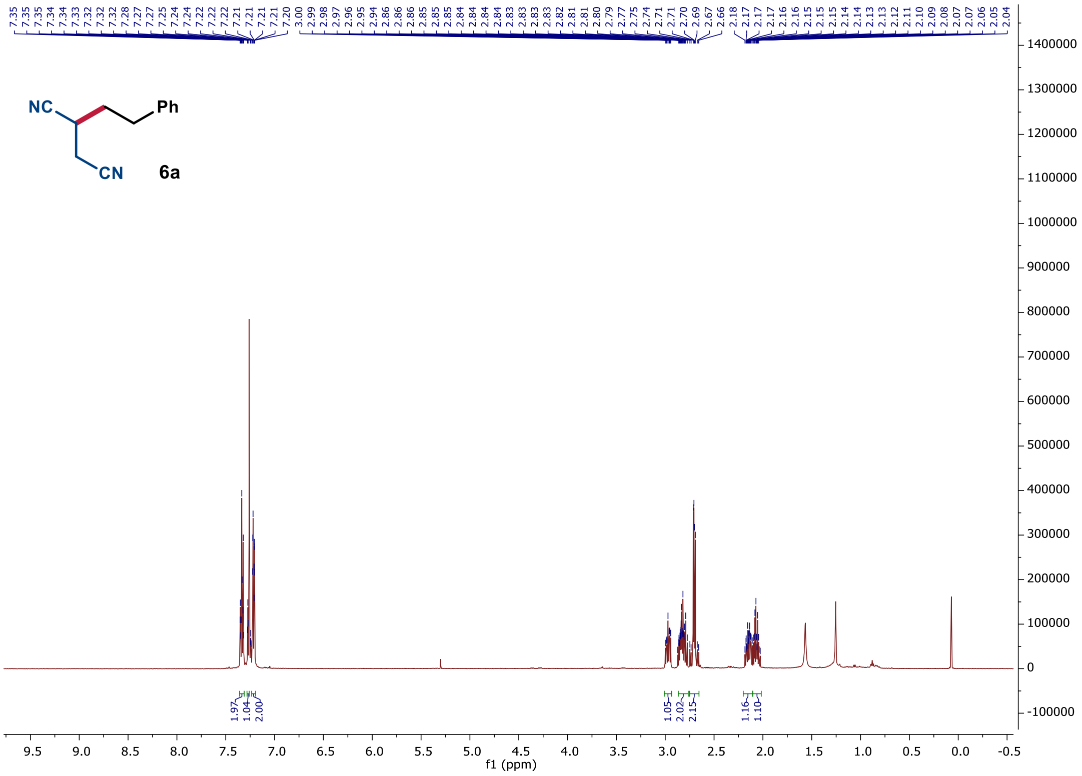

^{13}C NMR (126 MHz, $CDCl_3$) of **6a**

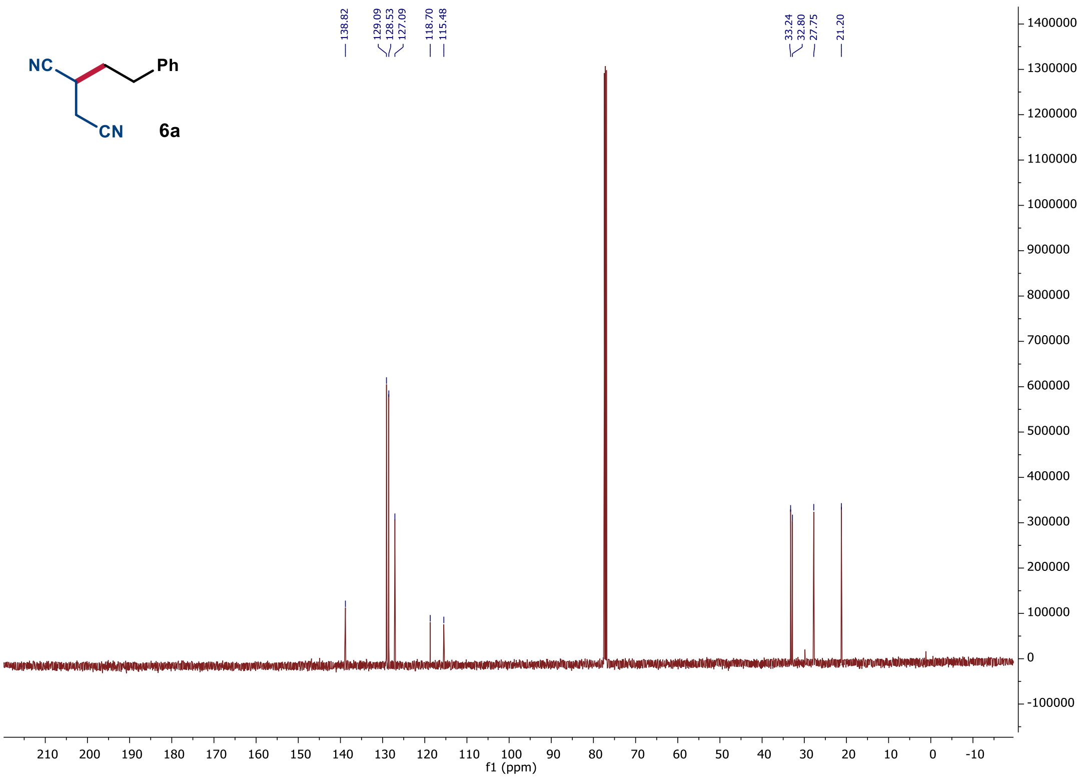

^{1}H NMR (500 MHz, $CDCl_3$) of **6b**

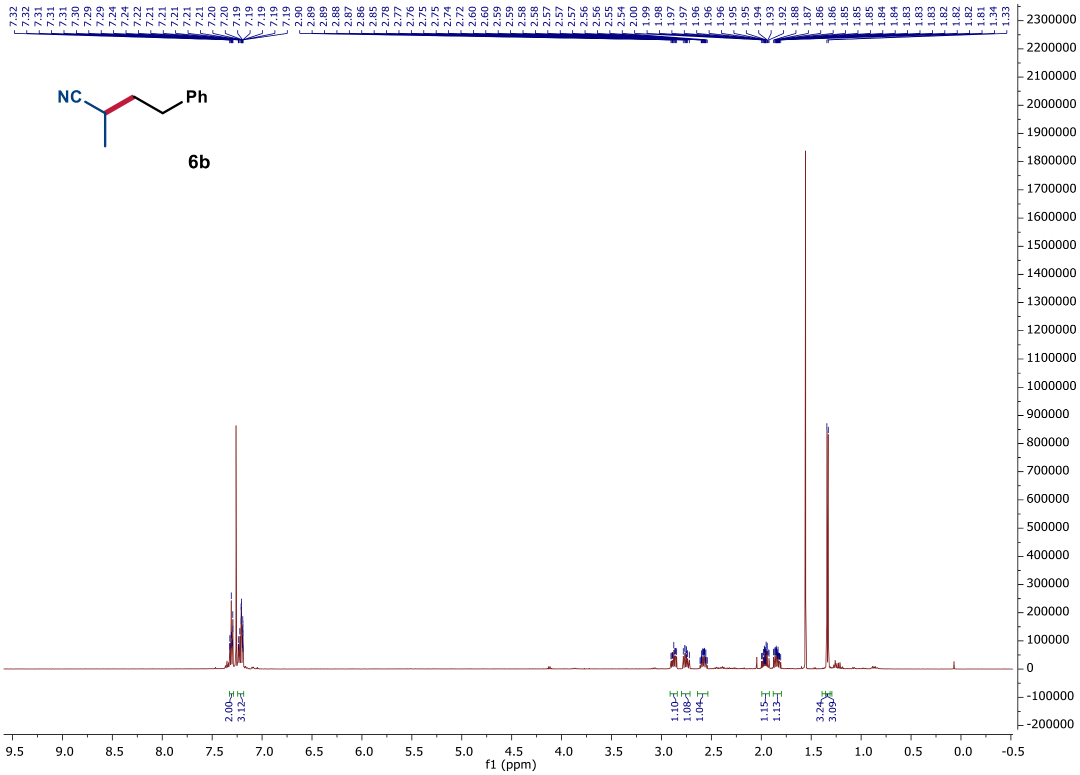

^{13}C NMR (126 MHz, $CDCl_3$) of **6b**

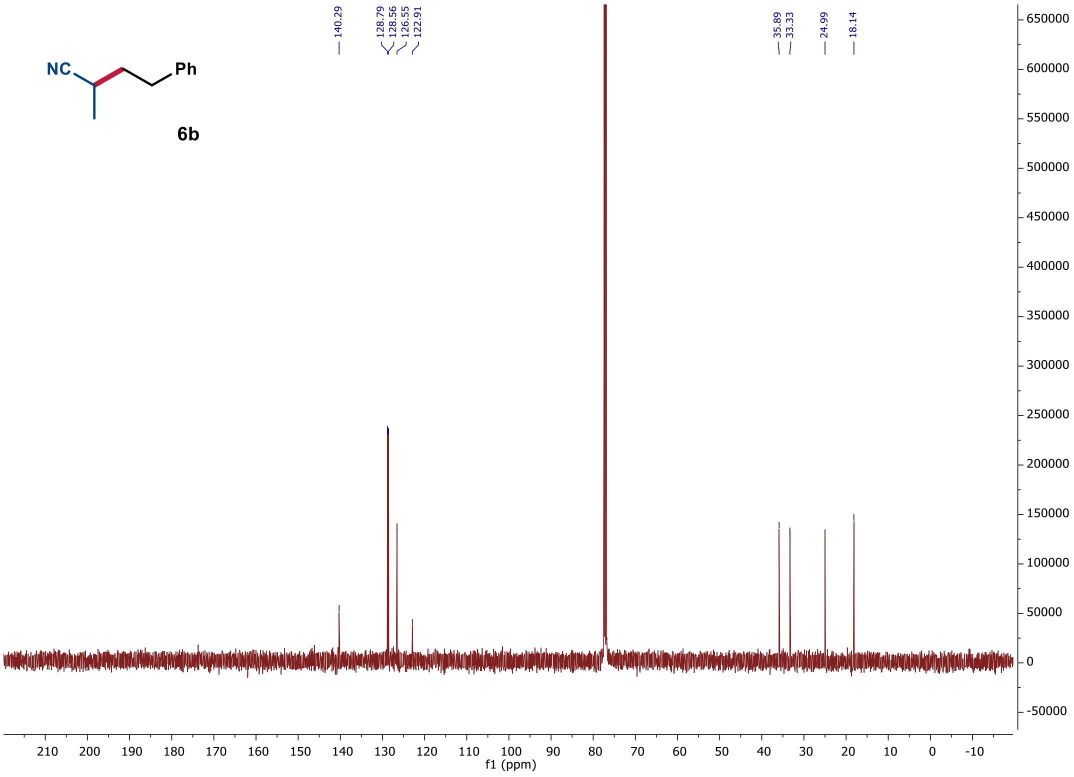

^{1}H NMR (400 MHz, $CDCl_3$) of **6c**

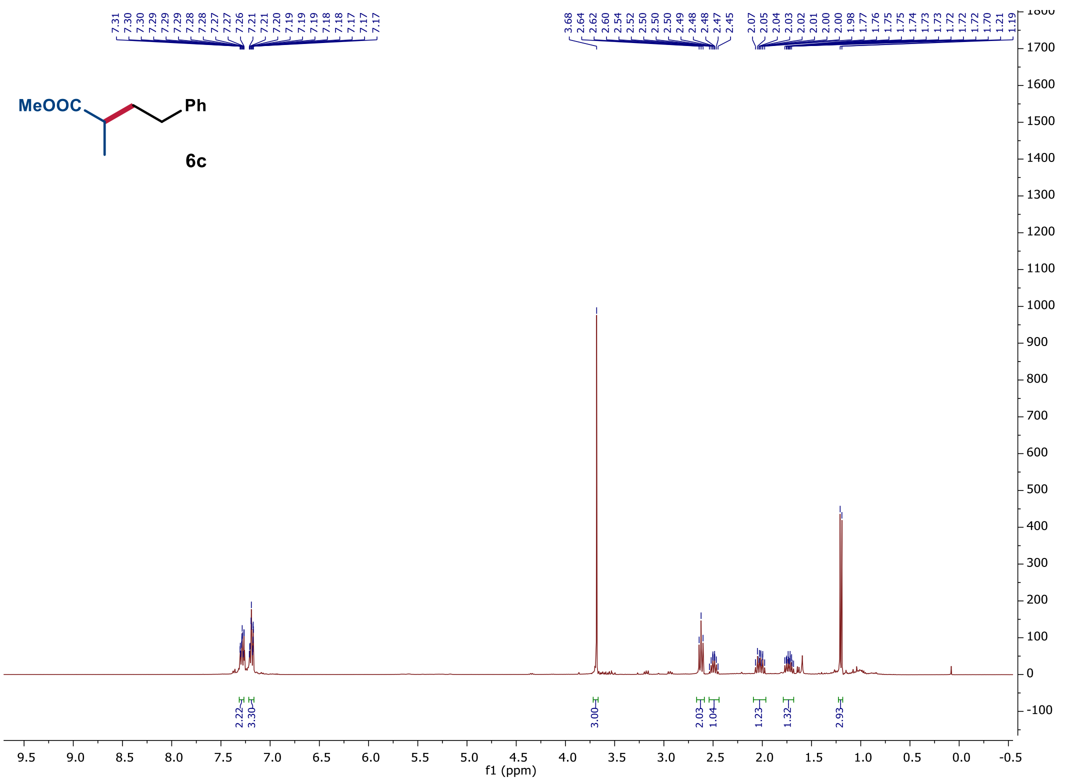

^{13}C NMR (101 MHz, $CDCl_3$) of **6c**

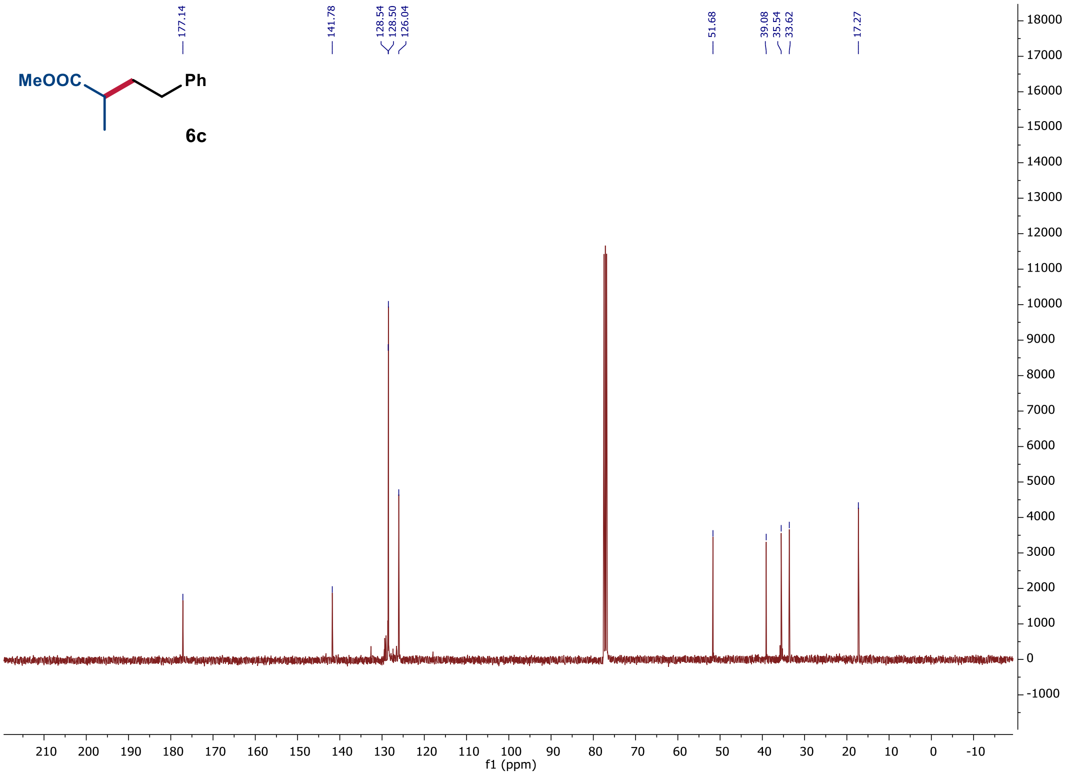

^{1}H NMR (500 MHz, $CDCl_3$) of **6d**

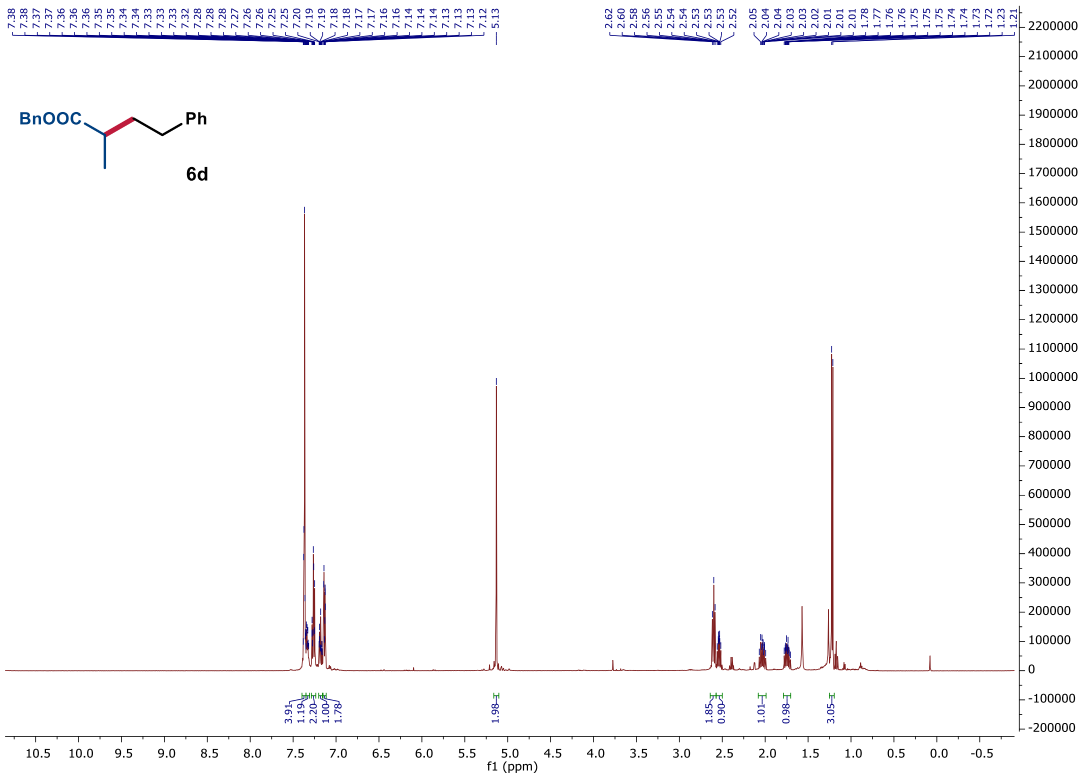

^{13}C NMR (126 MHz, $CDCl_3$) of **6d**

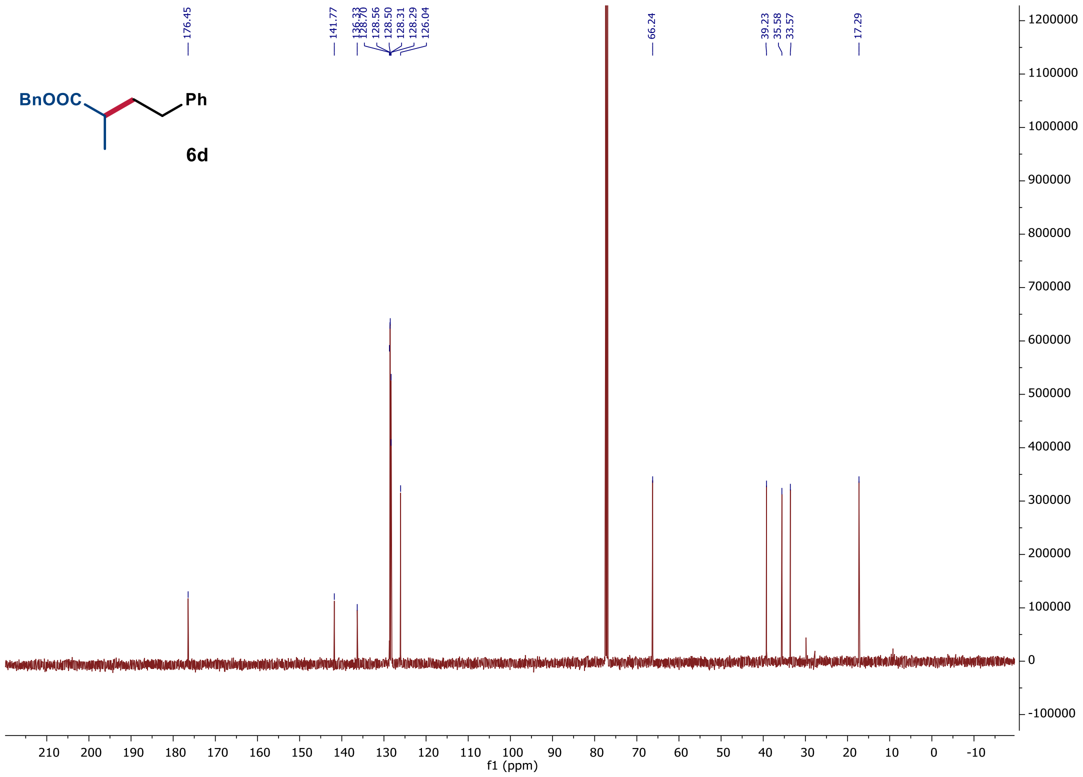

^{1}H NMR (400 MHz, $CDCl_3$) of **6e**

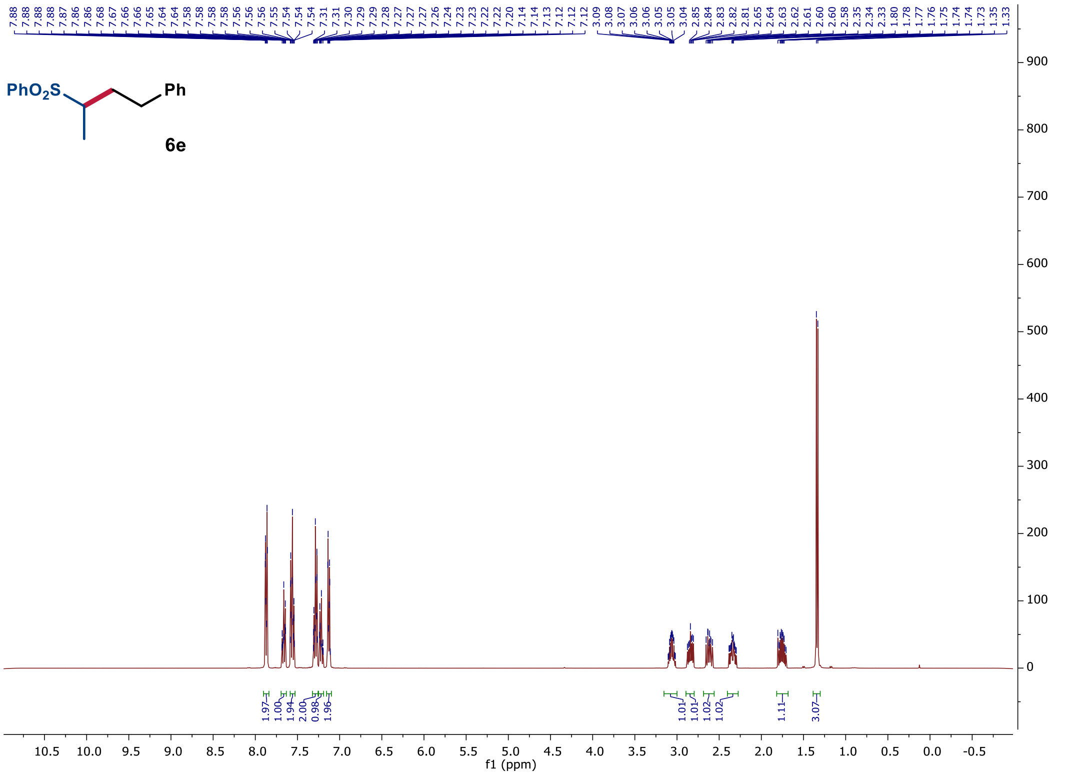

^{13}C NMR (101 MHz, $CDCl_3$) of **6e**

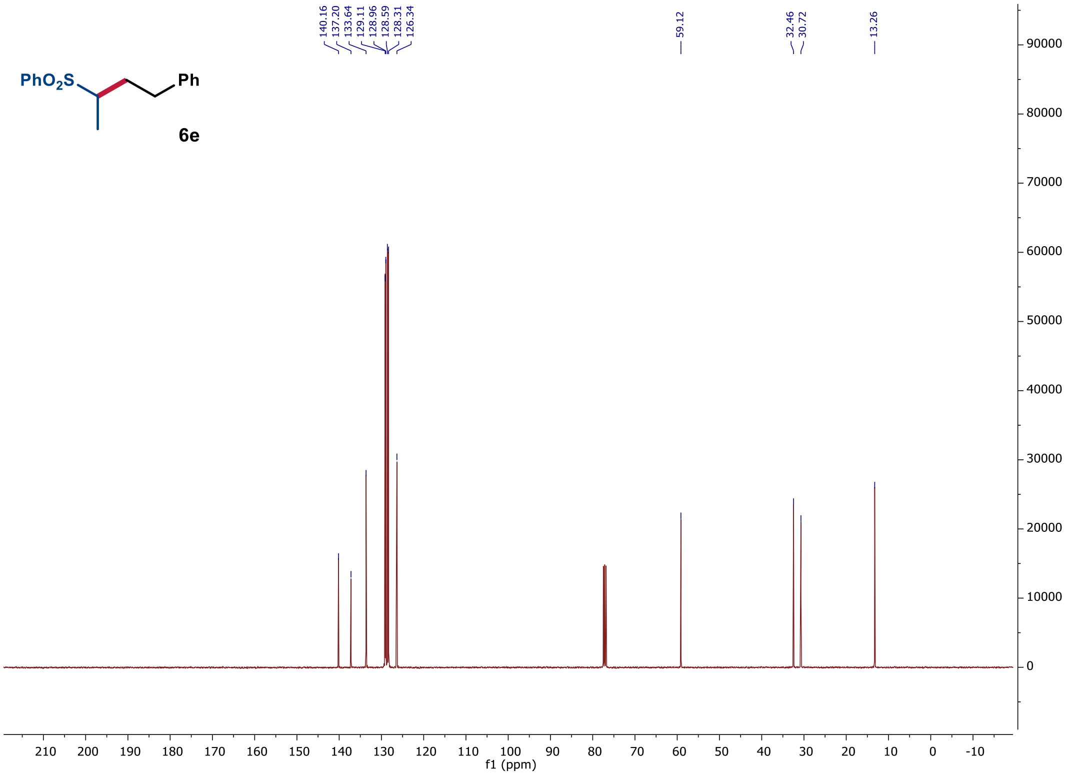

^{1}H NMR (300 MHz, $CDCl_3$) of **6f**

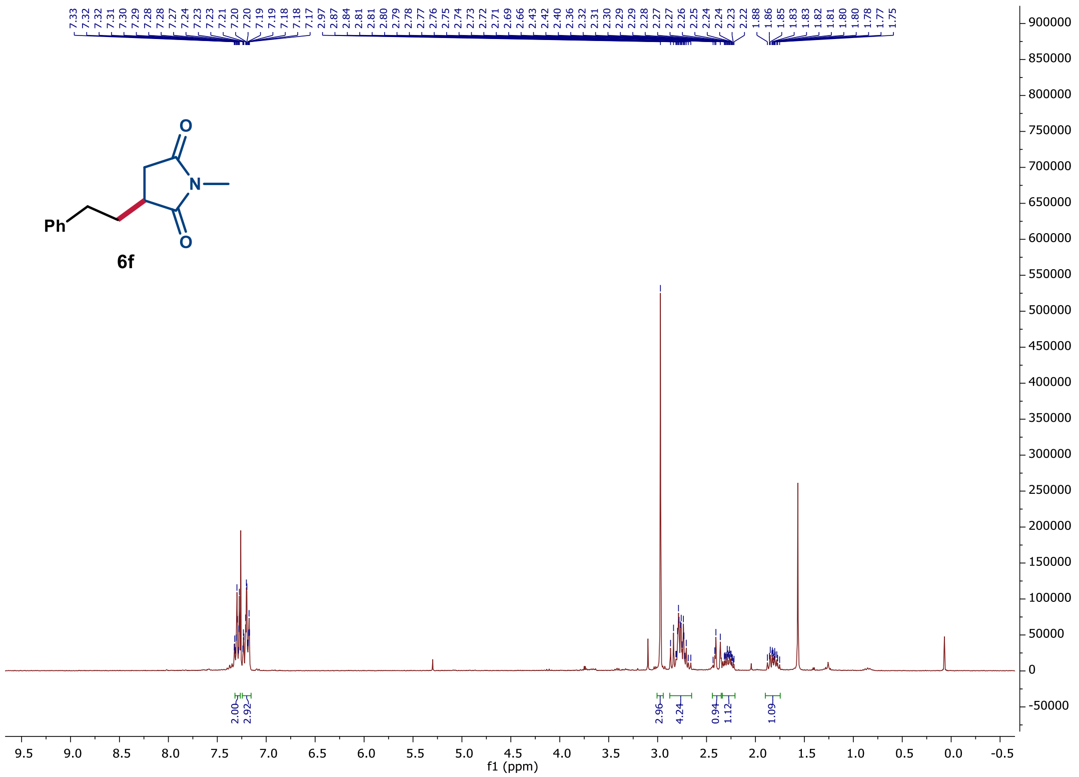

^{13}C NMR (101 MHz, $CDCl_3$) of **6f**

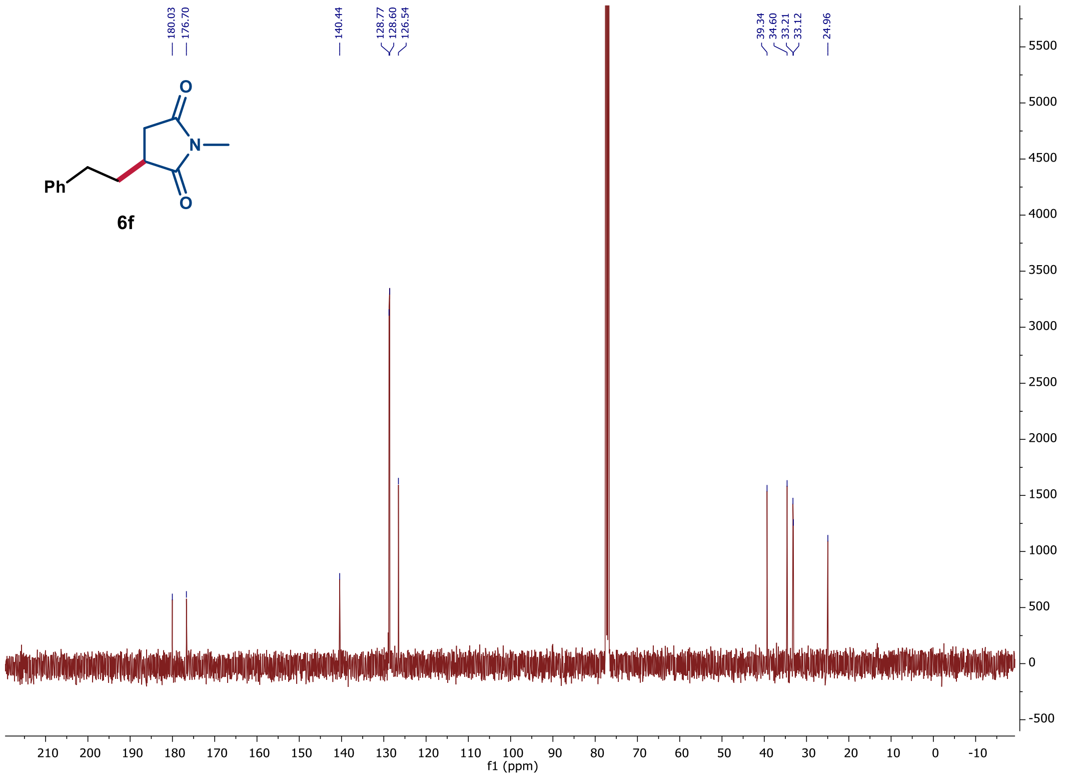

^{1}H NMR (500 MHz, $CDCl_3$) of **6g**

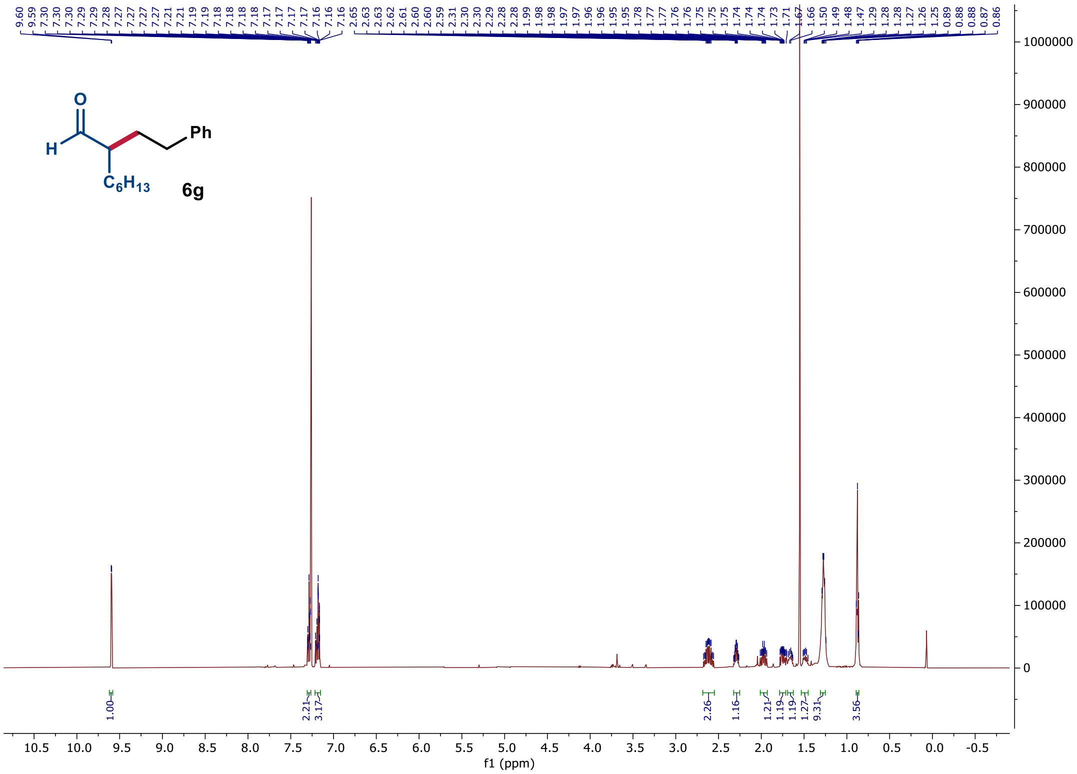

^{13}C NMR (126 MHz, $CDCl_3$) of **6g**

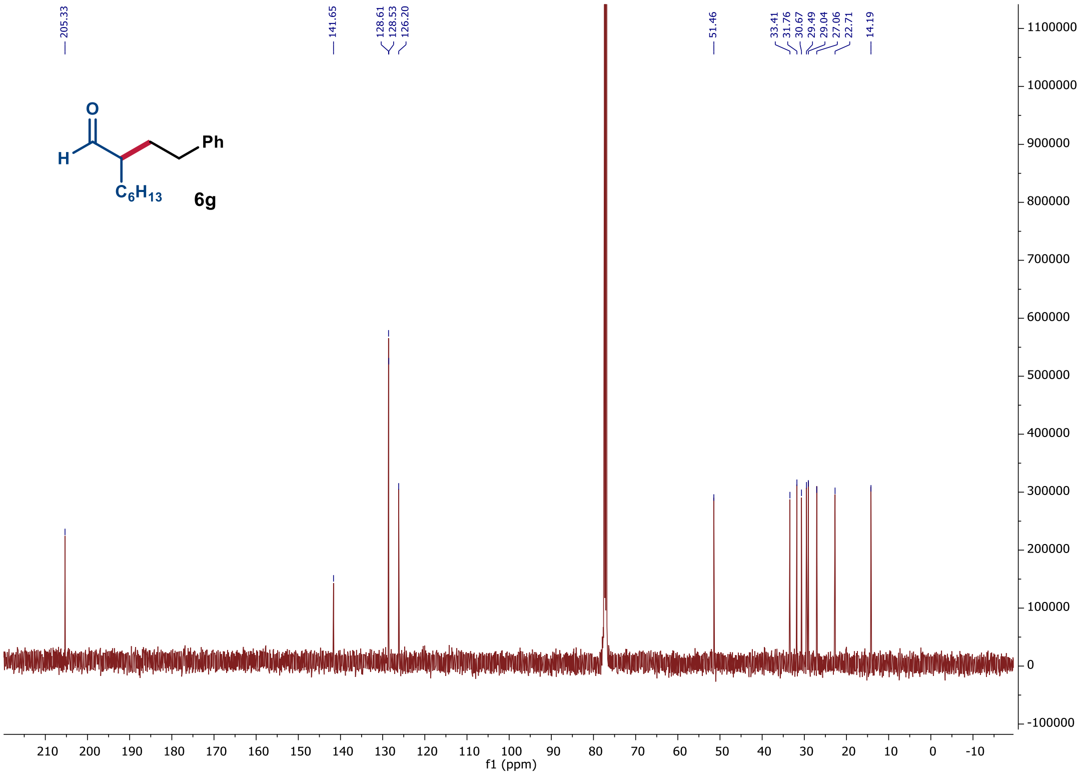

^{1}H NMR (500 MHz, $CDCl_3$) of **6h**

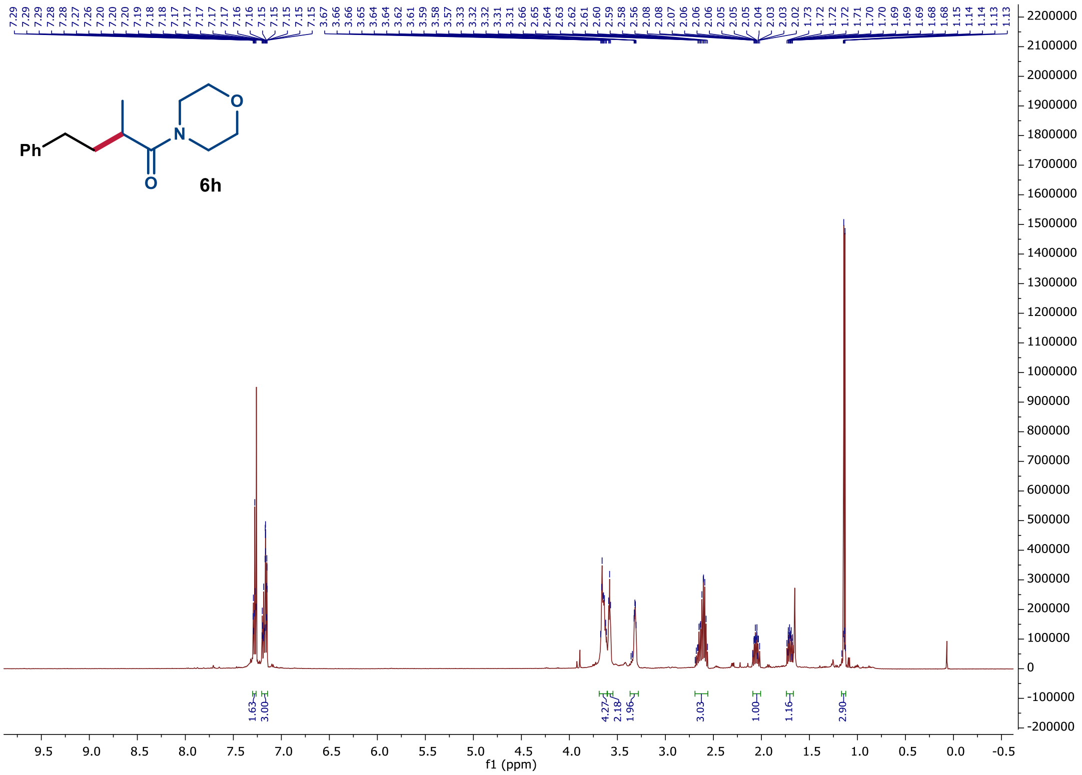

^{13}C NMR (126 MHz, $CDCl_3$) of **6h**

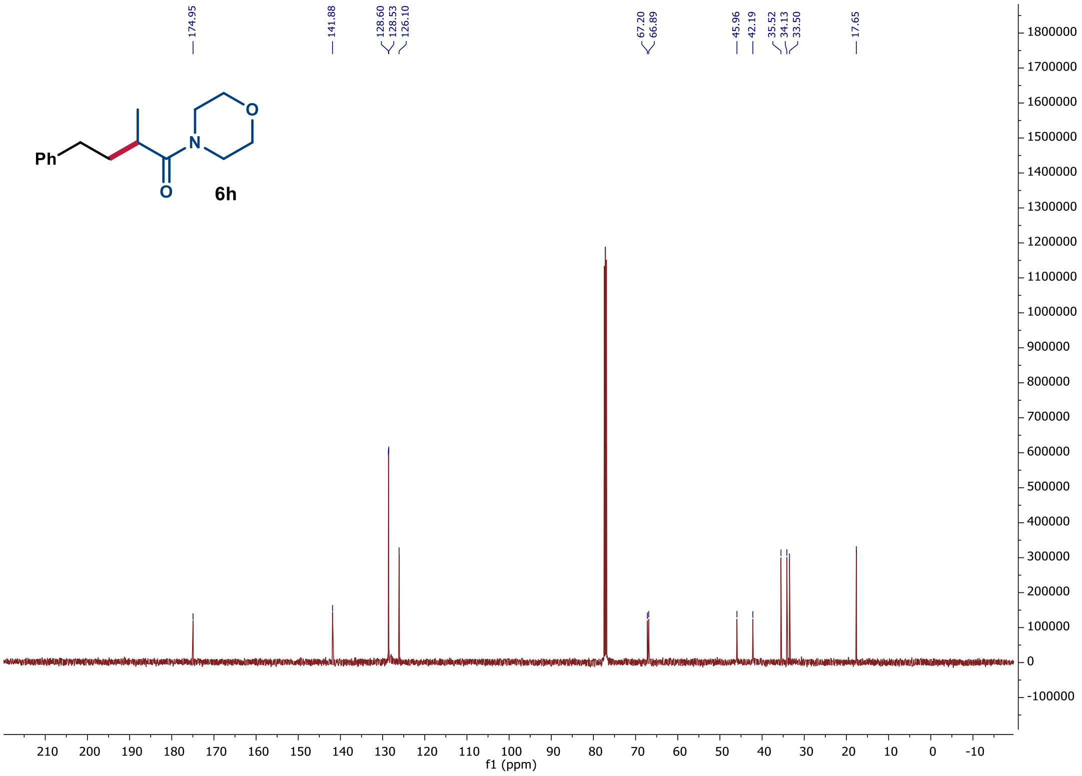

^{1}H NMR (400 MHz, $CDCl_3$) of **6i**

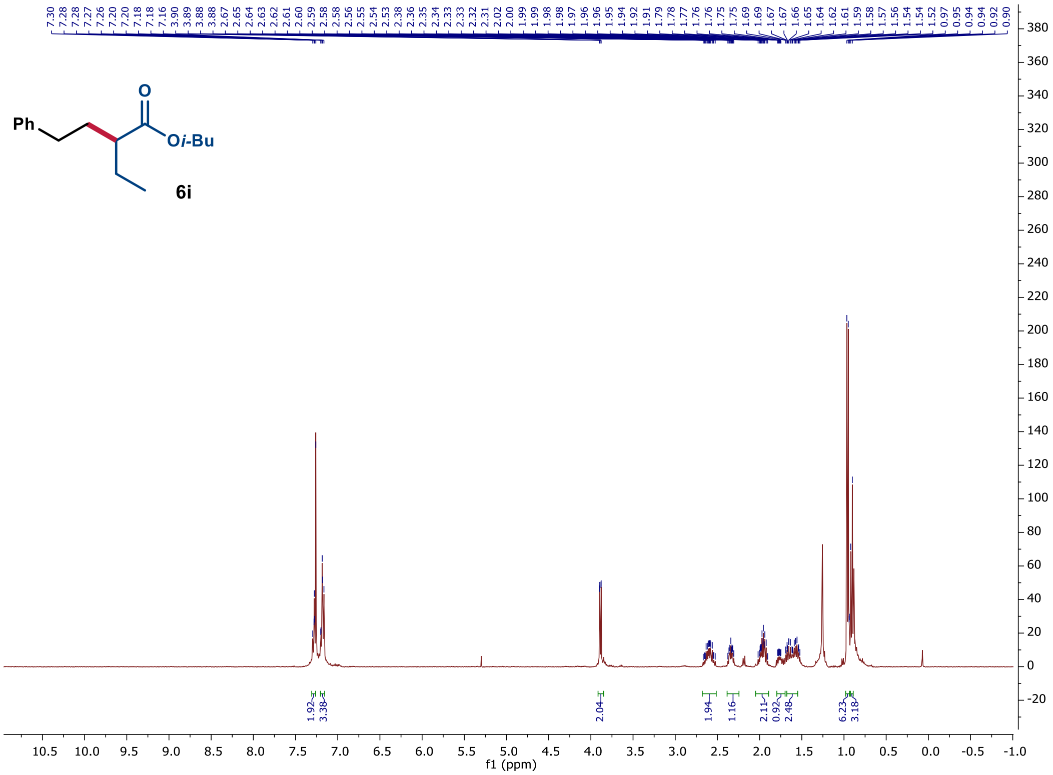

^{13}C NMR (101 MHz, $CDCl_3$) of **6i**

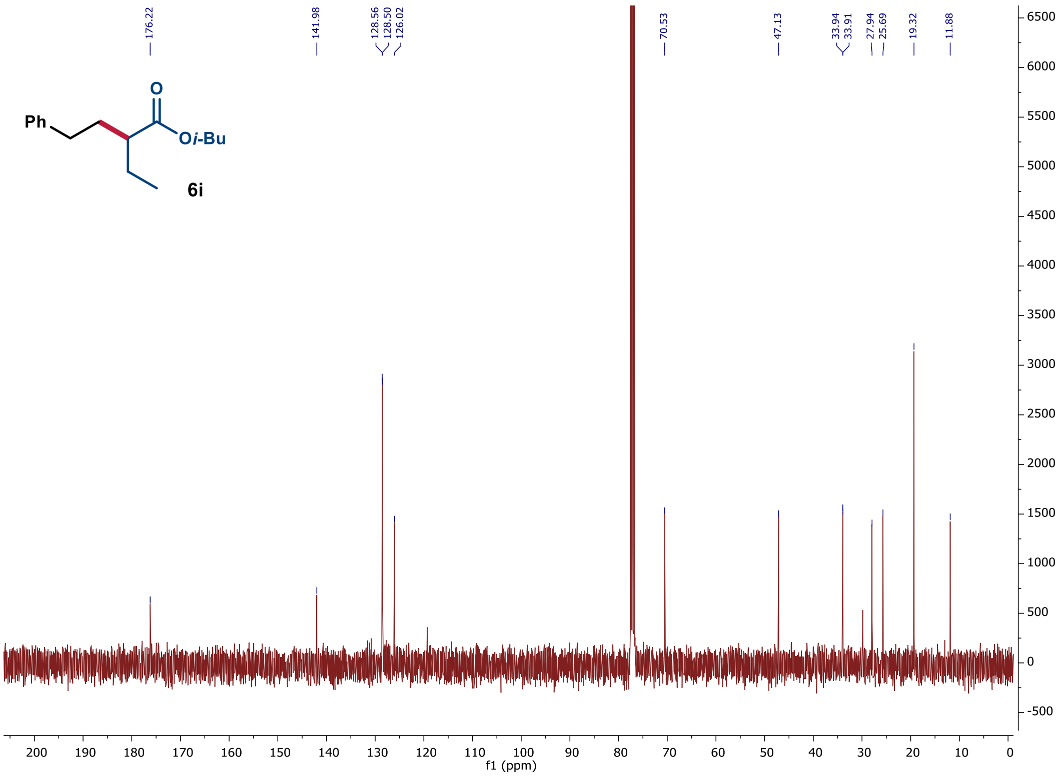

^{1}H NMR (500 MHz, $CDCl_3$) of **6j**

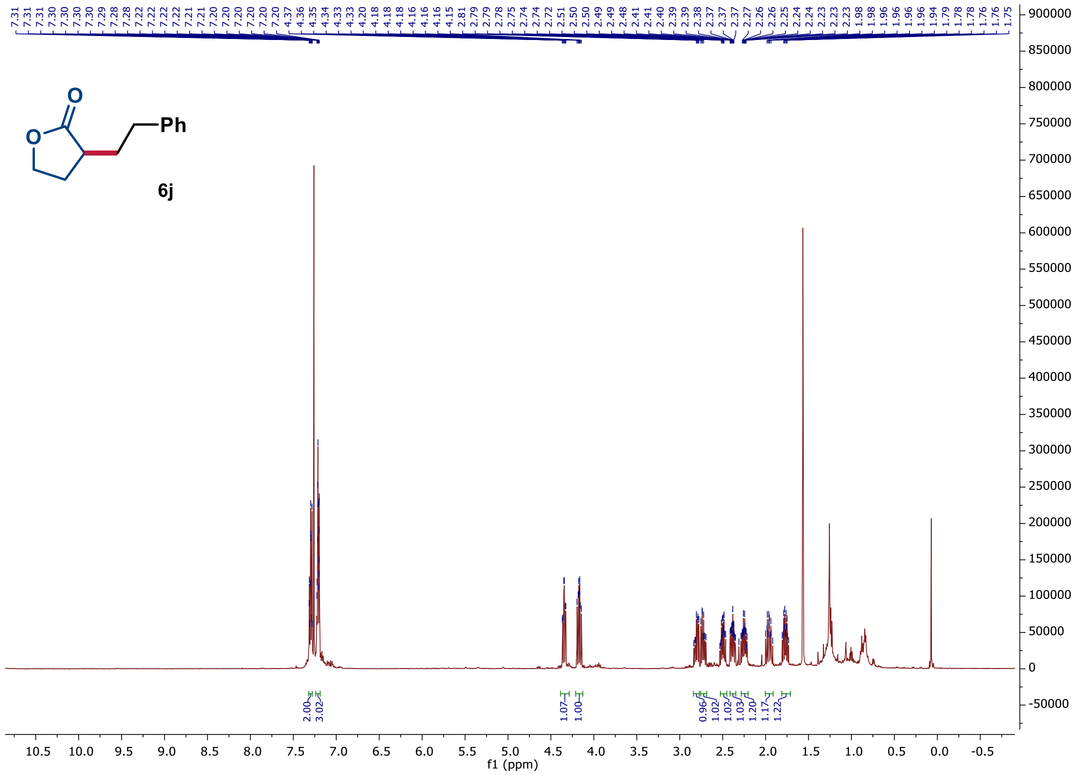

^{13}C NMR (126 MHz, $CDCl_3$) of **6j**

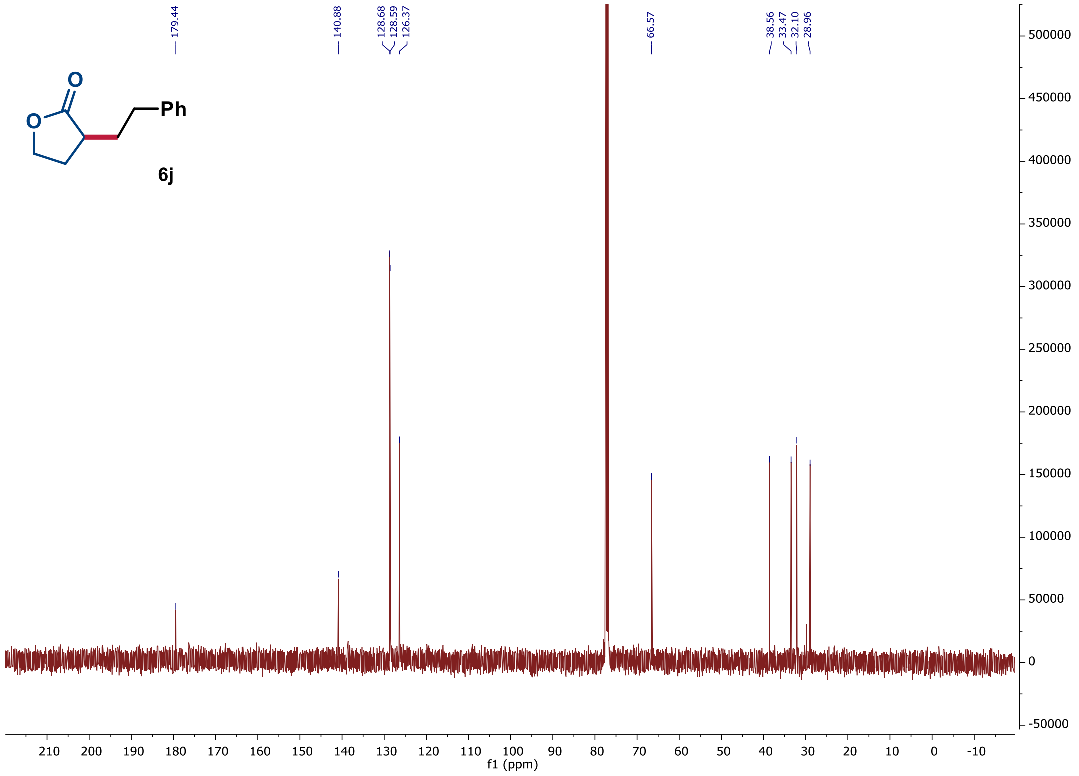

^{1}H NMR (500 MHz, $CDCl_3$) of **6k**

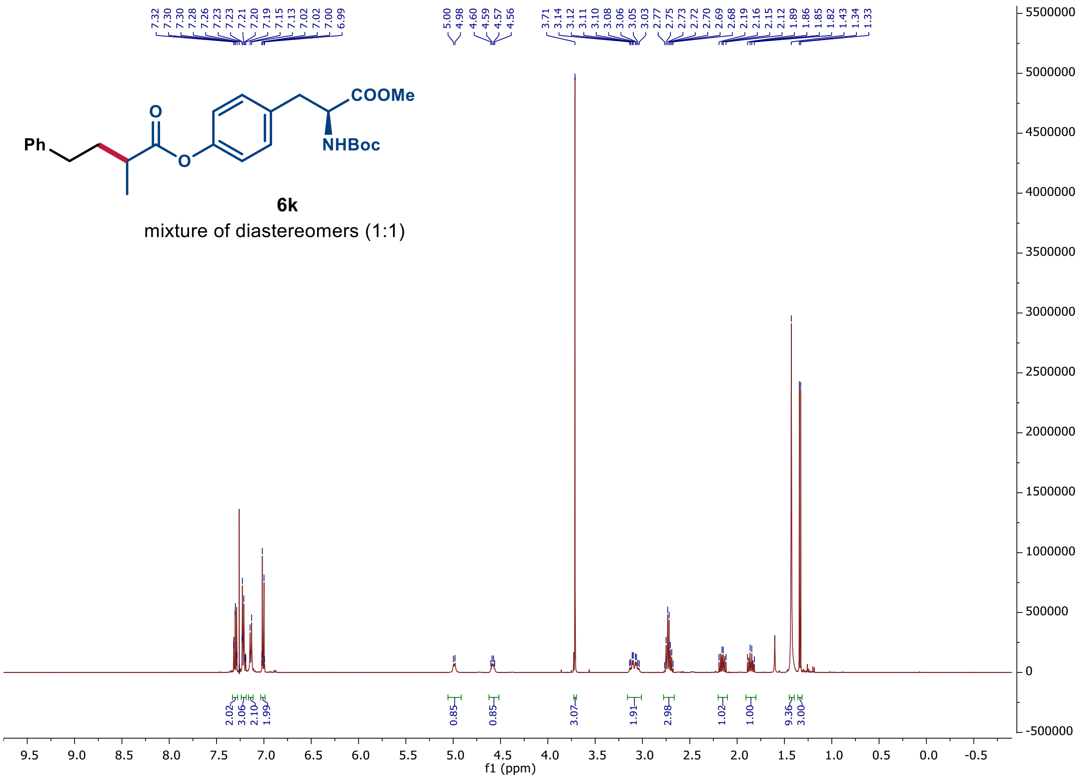

^{13}C NMR (126 MHz, $CDCl_3$) of **6k**

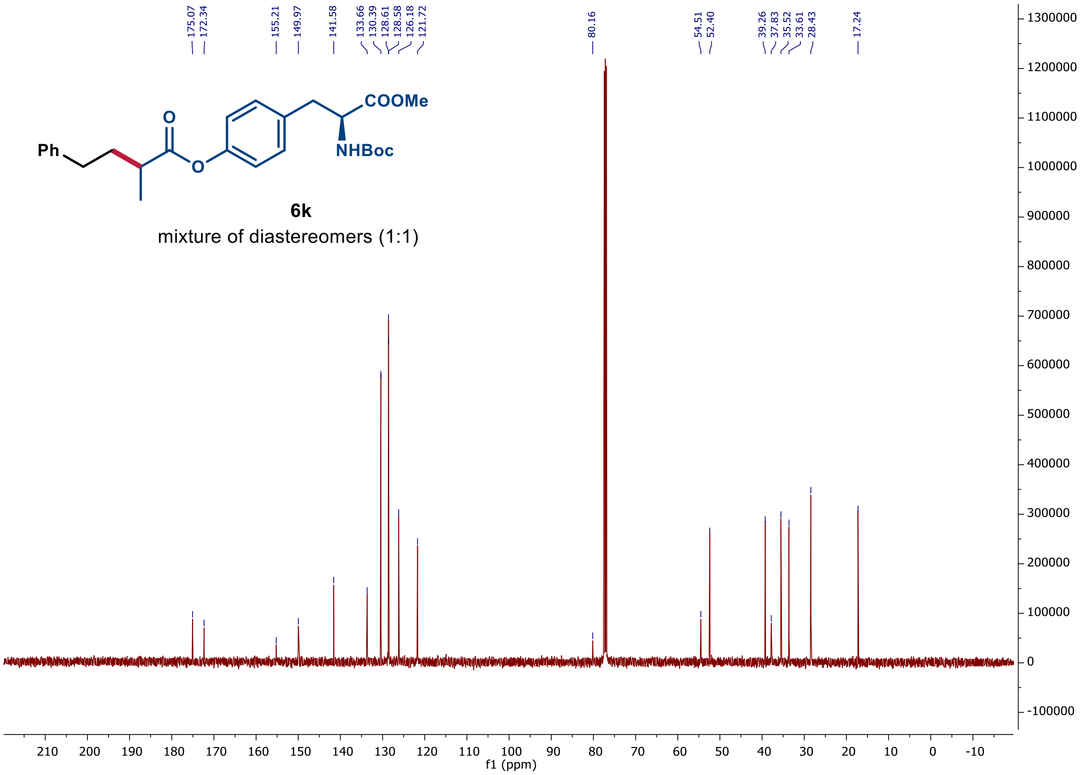

^{1}H NMR (500 MHz, $CDCl_3$) of **6l**

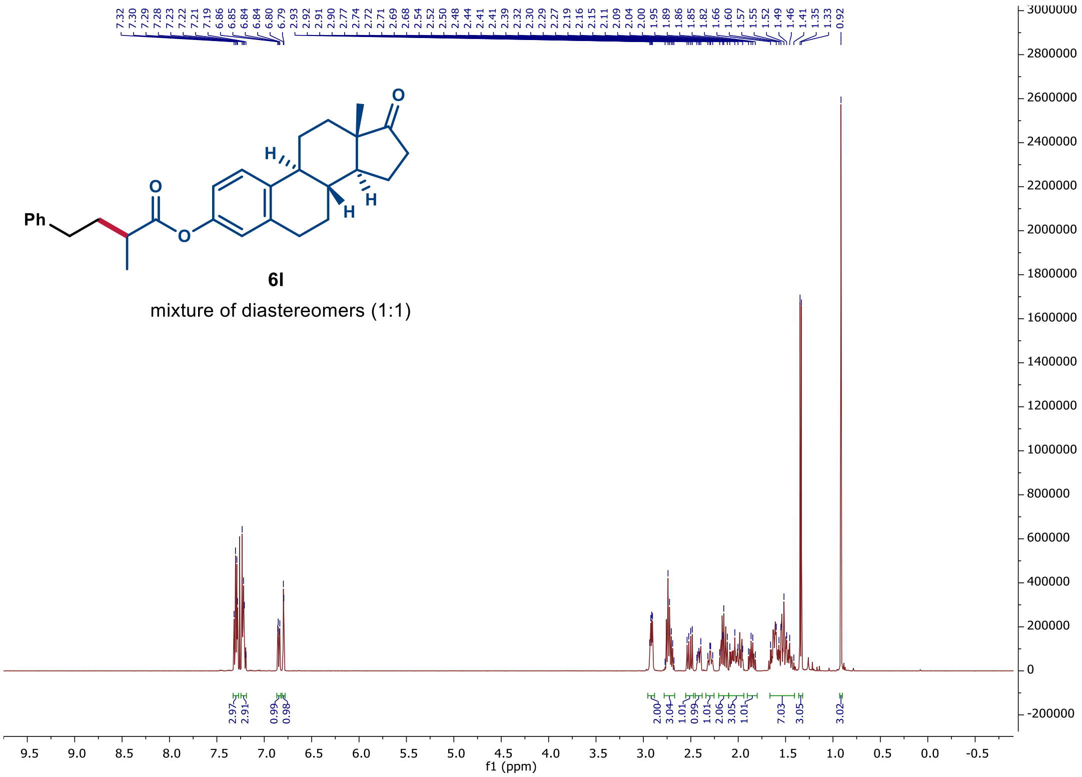

^{13}C NMR (126 MHz, $CDCl_3$) of **6l**

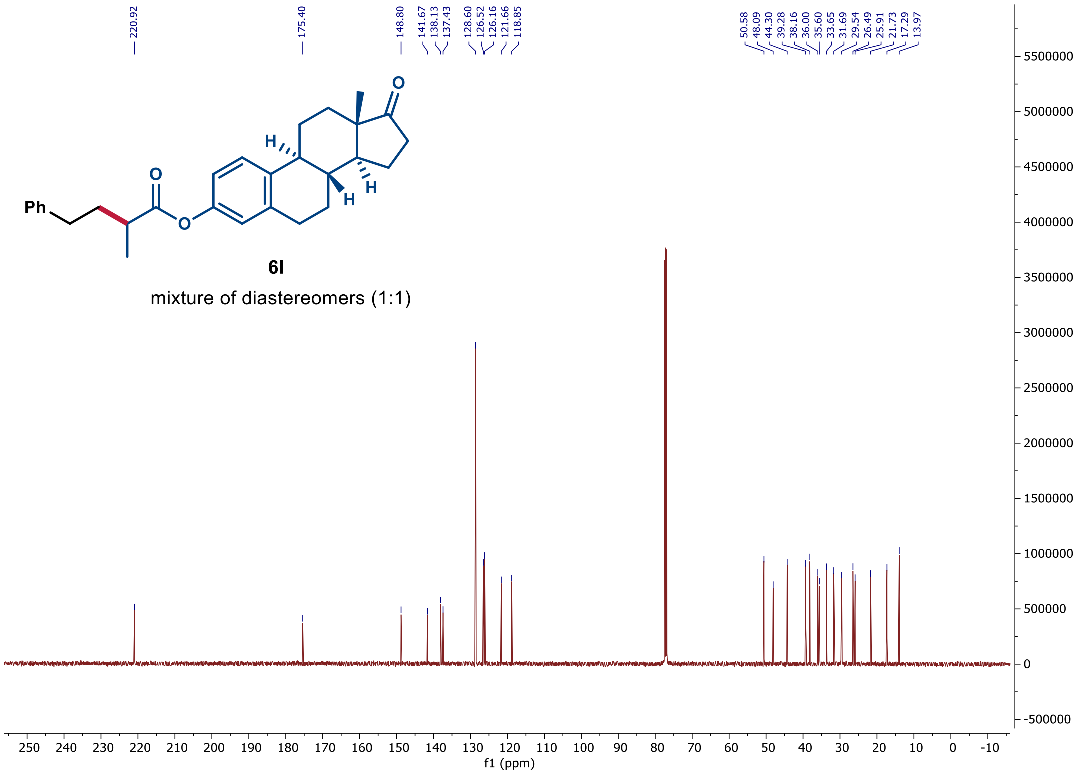

^{1}H NMR (500 MHz, $CDCl_3$) of **6m**

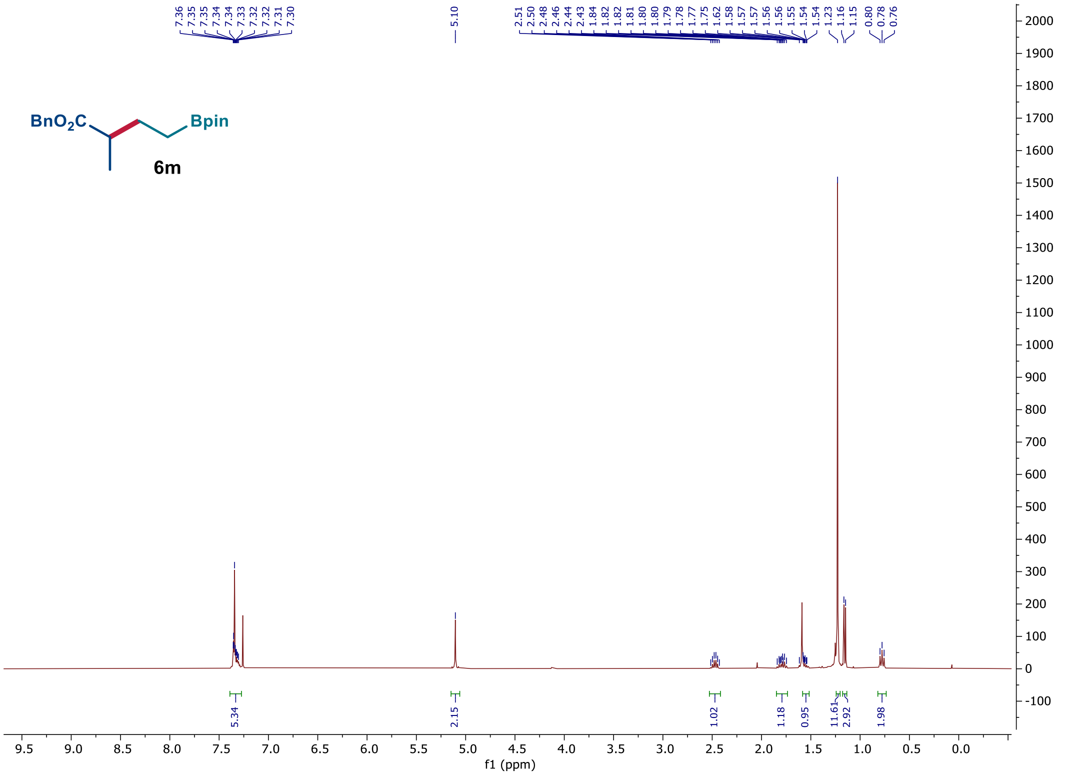

^{13}C NMR (101 MHz, $CDCl_3$) of **6m**

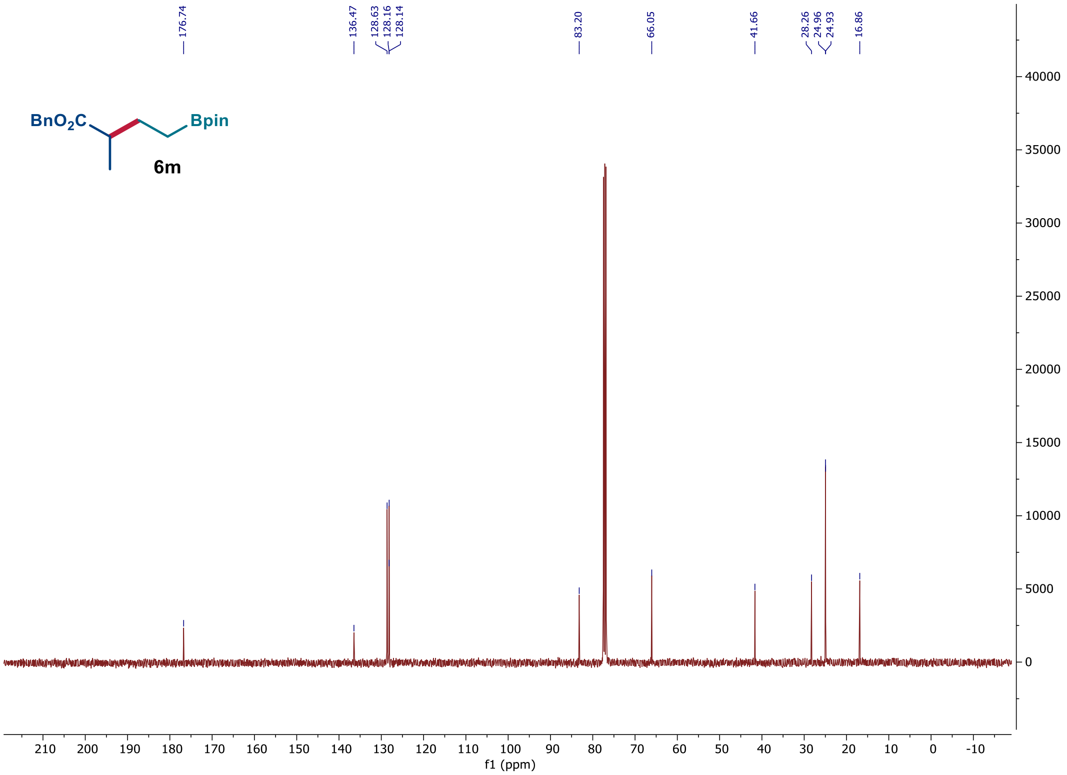

^{1}H NMR (400 MHz, $CDCl_3$) of **6n**

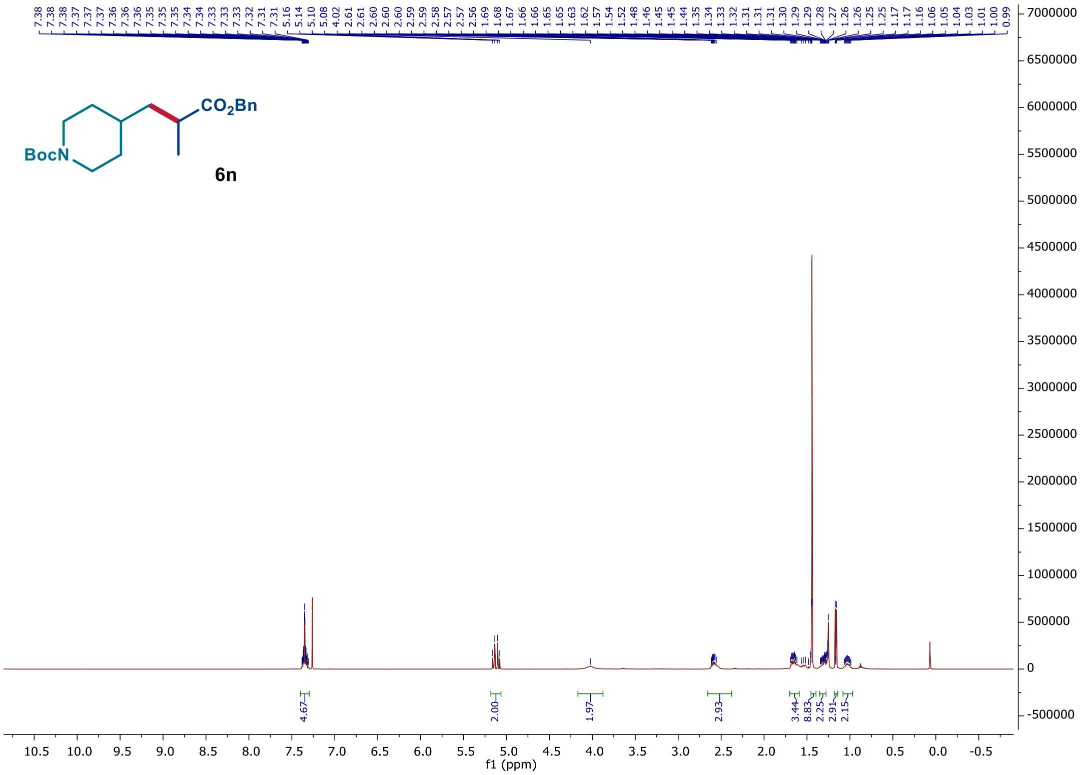

^{13}C NMR (101 MHz, $CDCl_3$) of **6n**

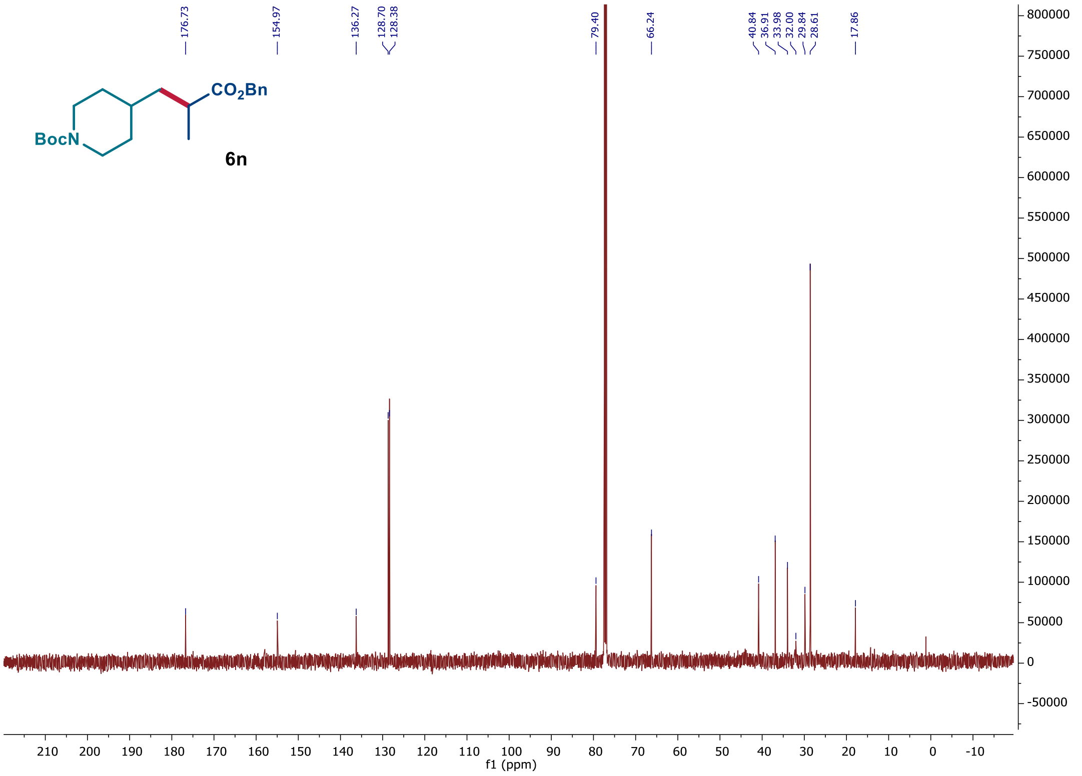

^{1}H NMR (500 MHz, $CDCl_3$) of **8**

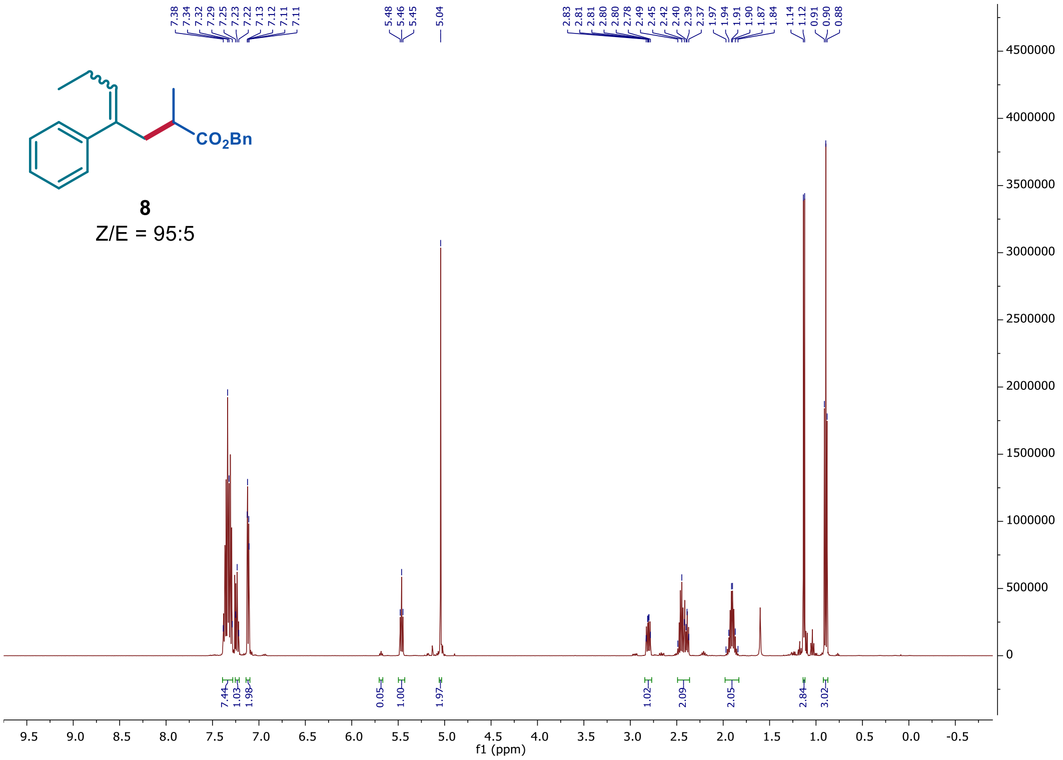

^{13}C NMR (126 MHz, $CDCl_3$) of **8**

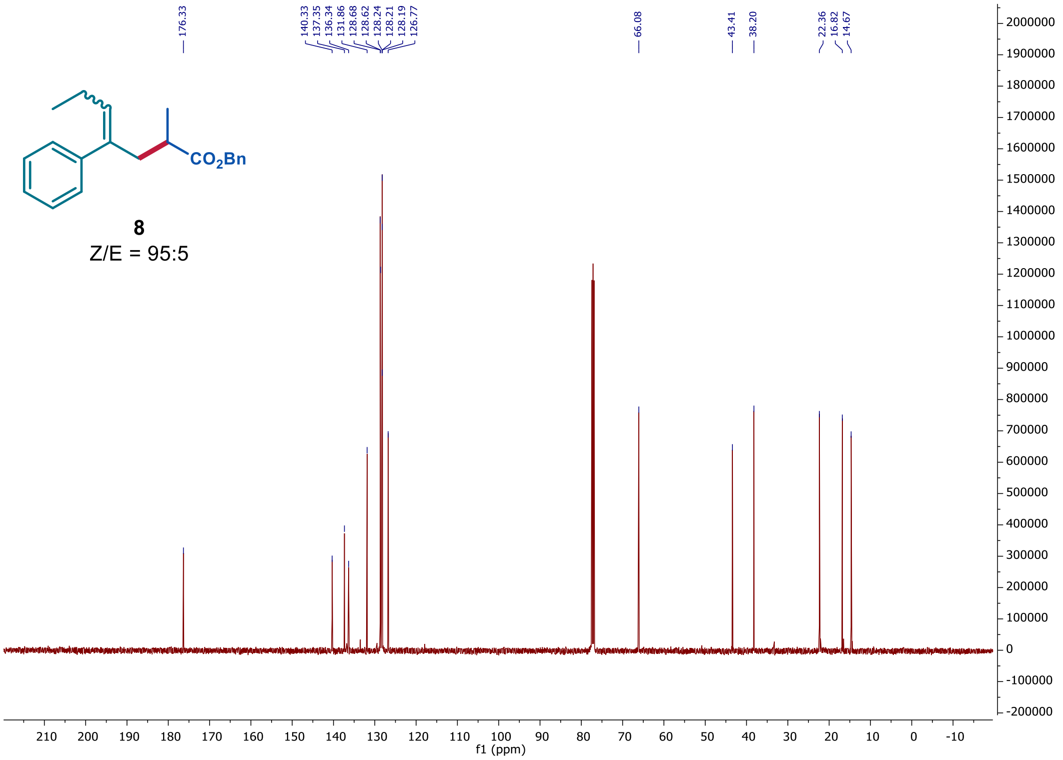

Supplement: Supplementary file 1 — ja3c11285_si_001.pdf [file ja3c11285_si_001.pdf]
